# Globin E is a myoglobin-related, respiratory protein highly expressed in lungfish oocytes

**DOI:** 10.1038/s41598-018-36592-w

**Published:** 2019-01-22

**Authors:** Julia Lüdemann, Kellen Matos Verissimo, Kimberley Dreger, Angela Fago, Igor Schneider, Thorsten Burmester

**Affiliations:** 1 Institute of Zoology, University of Hamburg, D-20146 Hamburg, Germany; 2 Instituto de Ciências Biológicas, Universidade Federal do Pará, Belém, PA Brazil; 3 Department of Bioscience, Aarhus University, DK-8000 Aarhus C, Denmark

## Abstract

Globins are a classical model system for the studies of protein evolution and function. Recent studies have shown that – besides the well-known haemoglobin and myoglobin – additional globin-types occur in vertebrates that serve different functions. Globin E (GbE) was originally identified as an eye-specific protein of birds that is distantly related to myoglobin. *GbE* is also present in turtles and the coelacanth but appeared to have been lost in other vertebrates. Here, we show that GbE additionally occurs in lungfish, the closest living relatives of the tetrapods. Each lungfish species harbours multiple (≥5) *GbE* gene copies. Surprisingly, GbE is exclusively and highly expressed in oocytes, with mRNA levels that exceed that of myoglobin in the heart. Thus, GbE is the first known oocyte-specific globin in vertebrates. No *GbE* transcripts were found in the ovary or egg transcriptomes of other vertebrates, suggesting a lungfish-specific function. Spectroscopic analysis and kinetic studies of recombinant GbE1 of the South American lungfish *Lepidosiren paradoxa* revealed a typical pentacoordinate globin with myoglobin-like O_2_-binding kinetics, indicating similar functions. Our findings suggest that the multiple copies of *GbE* evolved to enhance O_2_-supply in the developing embryo of lungfish, analogous to the embryonic and fetal haemoglobins of other vertebrates. In evolution, GbE must have changed its expression site from oocytes to eyes, or vice versa.

## Introduction

A constant supply of oxygen (O_2_) is essential for aerobic organisms. The transport and storage of O_2_ in vertebrates are mediated by proteins that are members of the globin superfamily^1^. Some globins may also have other functions and are, for example, involved in the detoxification of reactive O_2_ species (ROS), NO metabolism, or signaling^1,2^. The best-known vertebrate globins are haemoglobin (Hb), which is a heterotetramer that transports O_2_ in the blood^3^, and myoglobin (Mb), which is a monomer in the heart and the skeletal muscles, where it facilitates the diffusion of O_2_ and enhances O_2_ storage^4^. Within recent years, six additional globins have been identified in vertebrates^1^. The function of neuroglobin (Ngb), which resides mainly in the nervous system^5^, is still uncertain^6,7^. There is evidence that Ngb plays a role in oxidative metabolism^8,9^. Cytoglobin (Cygb) is expressed in fibroblast-related cell types and some populations of neurons^10–12^. Cygb may supply O_2_ to specific enzymes and may detoxify ROS^7^. Androglobin (Adgb) expression is restricted to the testis^13^. While Hb, Mb, Ngb, Cygb, and Adgb occur in most vertebrates, the occurrence of the globins E, X, and Y (GbE, GbX, and GbY) is restricted to certain taxa. GbX emerged very early in the evolution of Metazoa but is – in vertebrates – only present in non-tetrapods, amphibians and some reptiles^14,15^. The GbX protein is bound to the cell membrane via N-terminal acylation^16,17^, where it may protect the cells from ROS^18^. GbY has an unknown function in some “basal” ray-finned fishes, amphibians, reptiles, and platypus, where it is broadly expressed at low levels^14,19^.

GbE was initially found in the eye of chicken^20^ and was additionally identified in the genomes of other birds^21–23^, the coelacanth^24^ and turtles^25^. Gene synteny and phylogenetic analyses suggest that Mb is the closest related globin type of GbE, although the divergence of these genes must have occurred before the radiation of the gnathostome classes^21–23,26^. Immunohistochemistry and quantitative realtime RT-PCR (qRT-PCR) studies showed that GbE is highly and almost exclusively expressed in the eye (thus its name) of chicken and turtles^25^. Estimates of total protein levels were ~10 µM GbE in the chicken retina, which is in the range of Mb in striated muscle cells^21^. Together, the available evidence is consistent with GbE having a Mb-like function in O_2_ supply to the metabolically highly active avian retina^21^.

Lungfish (Dipnoi) have received much scientific interest because of their ability to breathe air, their conserved morphology that remained largely unchanged since the Devoian, and their phylogenetically position as closest living relatives of the tetrapods^27–30^. There are six extant lungfish species that dwell in rivers and (seasonal) freshwater lakes in the tropics^31^. Four species of the genus *Protopterus* live in Africa, *Lepidosiren paradoxa* in South America and *Neoceratodus forsteri* in Australia. Nearly all vertebrates have only a single *Mb* gene, which is expressed in the skeletal and heart muscles. In striking contrast, the West African lungfish *P. annectens* harbours at least seven distinct *Mb* genes with tissue-specific expression patterns^32^. For example, distinct Mb paralogs occur in the heart and skeletal muscle, and highest levels of Mb mRNA were found in the brain. Recombinant paralogous Mb proteins of *P. annectens* display different O_2_ binding affinities and enzymatic activities (J. Lüdemann, A. Fago, T. Burmester, unpublished data). The data suggest that the lungfish Mb paralogs carry out distinct functions and that the Mb genes evolved by neofunctionalisation and/or subfunctionalisation after multiple gene duplications.

In the present study, we demonstrate the occurrence of multiple *GbE* genes in three lungfish species, which are expressed almost exclusively in the ovary. Together with the biochemical analyses of recombinant lungfish GbE, we propose that GbE may have an Mb-like role in O_2_ supply in the ovary of lungfish during development. Our findings add a novel level of complexity to the studies of globin evolution and function.

## Results

### Identification of GbE genes in lungfish species

**Figure 1:**
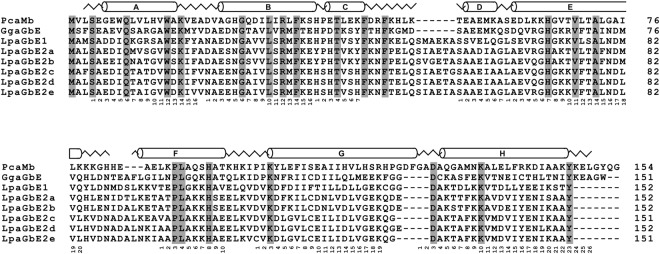
Comparison of the GbE and Mb amino acid sequences. The GbE sequences of the South American lungfish *L. paradoxa* (LpaGbE1–2e), the chicken (GgaGbE) and sperm whale Mb (PcaMb) were aligned. The secondary structure of sperm whale Mb is superimposed in the upper row, with α-helices designated A through H; the globin consensus numbering is given below the sequences. Strictly conserved residues are shaded in grey. The conserved globin residues, i.e. the proximal His F8 and the distal His E7, as well as the Phe CD1 are in boldface.

An assembly of transcriptomes of the South American lungfish *L. paradoxa* (Supplemental Information Table 1) revealed several globin genes. TBLASTN and BLASTN searches identified two *Hb* α, three *Hb* β, one *GbX*, one *GbY*, five *Mb* cDNA sequences, as well as multiple contigs that had resembled the sauropsid GbE (Supplemental Information Figs 1 and 2). No sequences that matched Ngb, Cygb, or Adgb were found in the available transcriptomes of *L. paradoxa*. The GbE cDNA sequences were verified by backmapping of the reads, reassembled if required, and finally revealed six distinct GbE transcripts, which were named *LpaGbE1* and LpaGbE2a to e. (Fig. 1). We should note that the nomenclature is provisional until a full representation of lungfish *GbE* genes is achieved. *LpaGbE1*, *2a*, and *2c* could be further verified by RT-PCR and sequencing. However, some sequences obtained by RT-PCR could not be assembled from the Illumina reads (which derived from a different specimen), indicating either technical issues such as hybrid sequences generated by the PCR step, or biological causes such as different alleles in the population, along with gene conversion or crossing over among the closely related *GbE* genes. Within the coding region of 456 or 459 bp, respectively, the six *LpaGbE* sequences differ between 2.6 and 34.8%. After translation, the differences were 4.7 to 41.4% on the amino acid level. The largest difference was found between *LpaGbE1* and *LpaGbE2b*.

We assembled the publically available transcriptomes of the West African lungfish *P. annectens*^33^ and searched them for GbE genes. We found five distinct GbE cDNA sequences, which differ between 1.8 and 27.2% on the nucleotide and between 1.3 and 36.2% on the amino acids level. Because the sequences form two clades in phylogenetic analyses (see below), they were named *PanGbE1a* and *b*, and *PanGbE2a*-*c*, respectively.

Additionally, we generated 60,785,122 Illumina reads (150 nt, paired-end) from total RNA extracted from the ovary of the marbled lungfish *P. aethiopicus*. The reads were assembled and searched for GbE sequences, of which seven distinct cDNA sequences were identified. The sequences differ 2.4 to 27.7% on the nucleotide and 2.0 to 41.1% on the amino acid level. The sequences were named *PaeGbE1a*-*c*, and *PaeGbE2a*-*d*, respectively, based on the position in the phylogenetic tree (see below).

### Conservation and lungfish-specific amplification of *GbE* genes

Our studies resulted in a total of 18 novel GbE genes from three lungfish species with 151 or 152 amino acids. An alignment with the known GbE amino acid sequences of birds, turtles and coelacanth showed that all lungfish GbEs carry an insertion of six amino acids in the region between helices C and D, a deletion of four amino acids in the GH interhelical region, and are five amino acids shorter (Fig. 1). The maximum divergence within lungfish GbE amino acid sequences was 42.1%. The divergence of lungfish and coelacanth GbE was between 42.1 and 52.8% of the amino acid. Sauropsid and lungfish GbE amino acid sequences differed by up to 56.4%. No other globin is more closely related to the lungfish GbE sequences.

**Figure 2:**
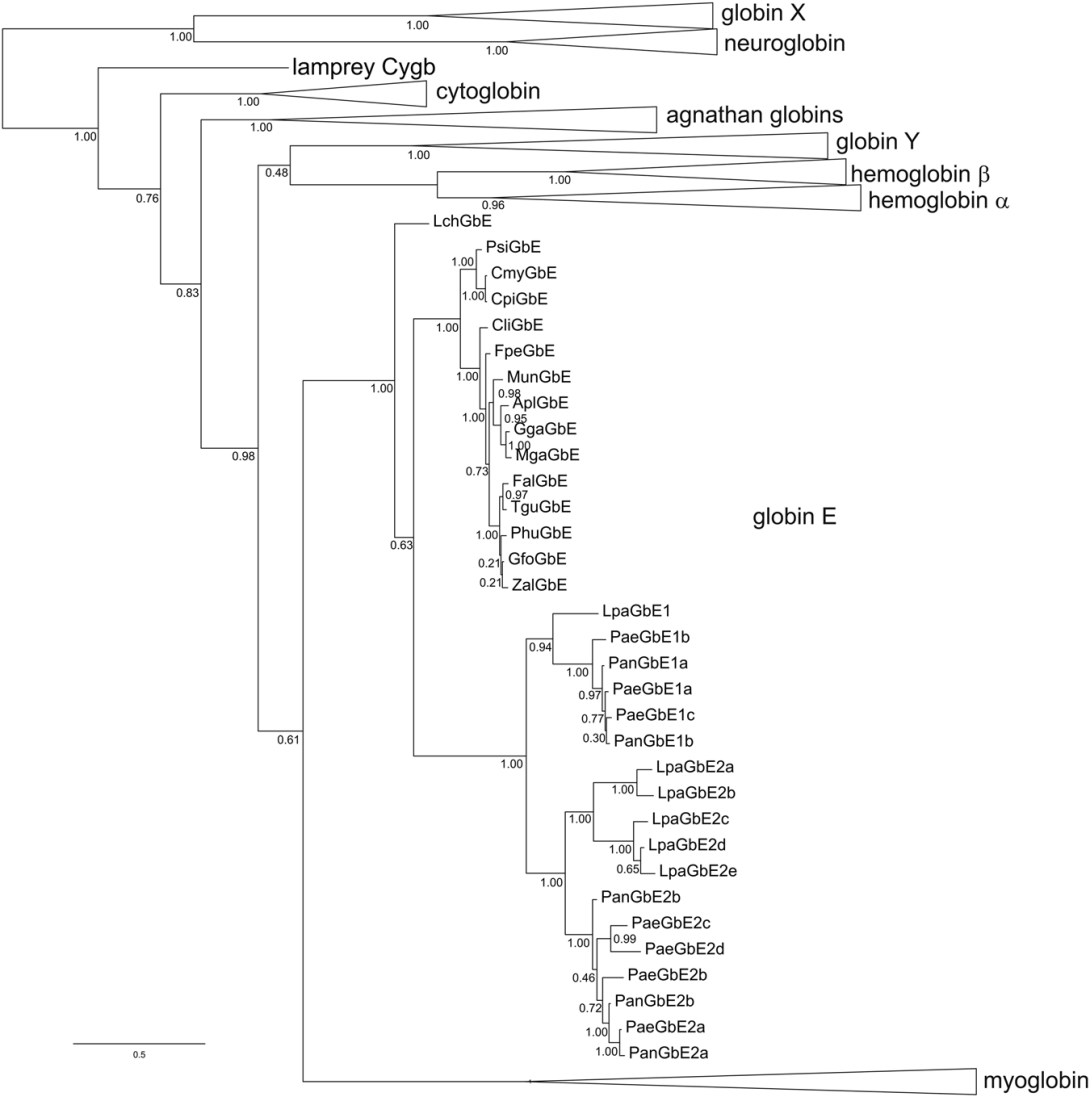
Bayesian phylogenetic tree of vertebrate globins. Tree reconstruction was carried out with the amino acid sequences assuming the LG model. The bar represents 0.1 substitutions per site. The numbers at the nodes are posterior probabilities. The different globin clades – except GbE – have been collapsed; the full tree is given in Supplemental Information Fig. 4. The species abbreviations are: Apl, *Anas platyrhynchos*; Cli, *Columba livia*; Cmy, *Chelonia mydas*; Cpi, *Chrysemys picta bellii*; Fal, *Ficedula albicollis*; Fpe, *Falco peregrinus*; Gfo, *Geospiza fortis*; Gga, *Gallus gallus*; Lch, *Latimeria chalumnae*; Lpa, *Lepidosiren paradoxa*; Mga, *Meleagris gallopavo*; Mun, *Melopsittacus undulatus*; Pae, *Protopterus aethiopicus*; Pan, *Protopterus annectens*; Phu, *Pseudopodoces humilis*; Psi, *Pelodiscus sinensis*; Tgu, *Taeniopygia guttata*; Zal, *Zonotrichia albicollis*.

Phylogenetic analyses using GbE and other globin amino acid sequences (Supplemental Information Fig. 3) confirmed previous studies, which found a relationship of GbE and Mb, although the support was not particularly high (0.61 posterior probability) (Fig. 2; Supplemental Information Fig. 4). Within the GbE clade, the overall topology of the tree followed the accepted relationships among gnathostome taxa. The coelacanth GbE formed the sister group of all other GbE sequences. Sauropsid and lungfish GbEs were two separate clades. The lungfish GbEs fell into two clades, of which one was formed by the GbE1 sequences, the other one by the *L. paradoxa* GbE2a-e along with the GbE2 proteins of the genus *Protopterus*.

### Expression of GbE in the lungfish ovary

**Figure 3:**
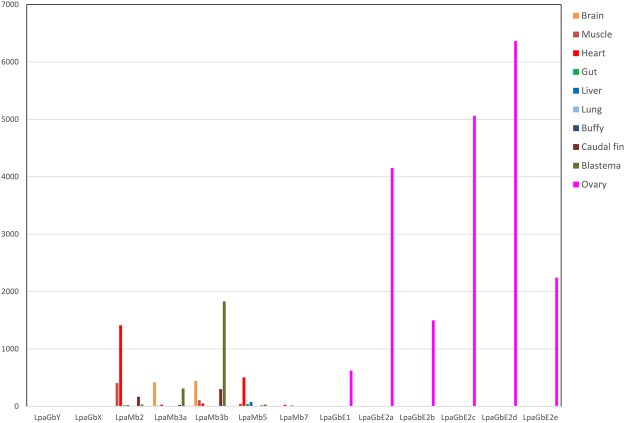
Expression of the *Mb* and *GbE* genes in selected *L. paradoxa* tissues. mRNA levels were estimated by RNA-Seq and are displayed as RPKM values. Transcriptome accession numbers are given in Supplemental Information Table 1. Note the dominant expression of LpaGbE1-2e in the ovary. The copy numbers are given in Supplemental Information Table 3. Log-scale data are presented in Supplemental Information Fig. 5. The tissue-specific expression per gene is given in Supplemental Information Figs 6 and 7.

**Figure 4:**
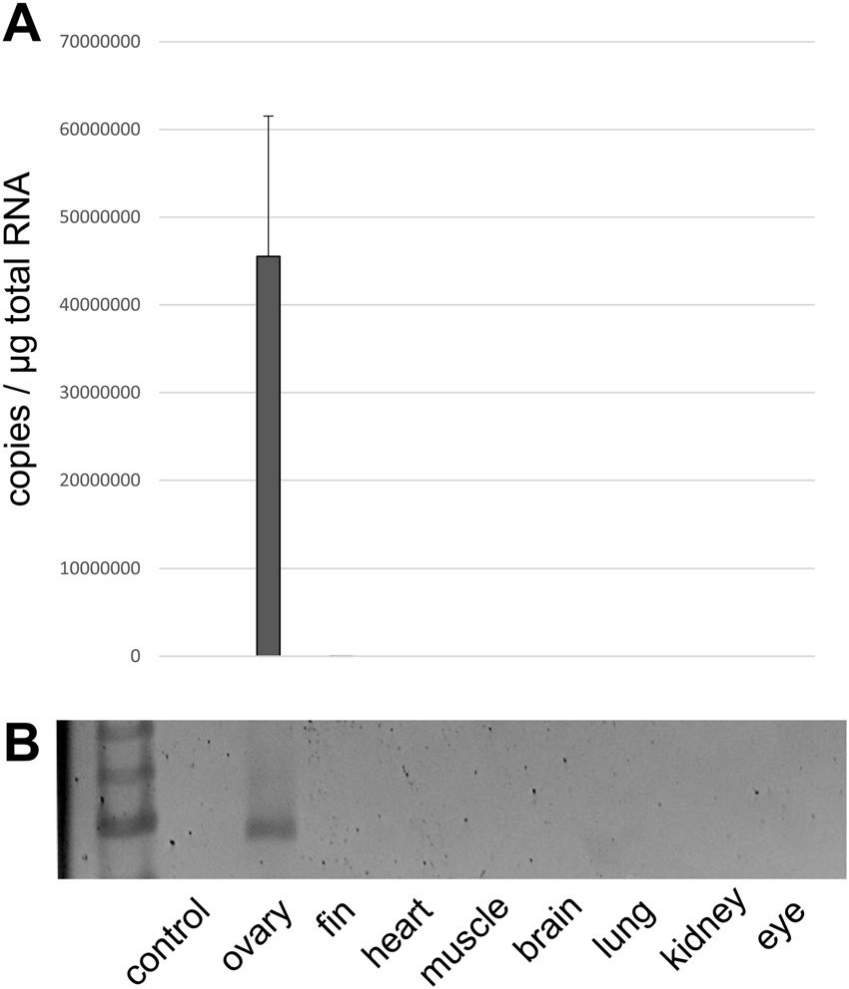
Expression of *GbE1* in selected *L. paradoxa* tissues. mRNA levels were determined by qRT-PCR (**A**) and RT-PCR (**B**). The standard deviations in (**A**) derive from three replicates. GbE1 was almost exclusively detected in the ovary.

We checked the expression levels of the six *GbE* by RNA-seq employing the transcriptomes of *L. paradoxa* (Fig. 3; Supplemental Information Figs 5–7). The five *Mb* genes, as well as *GbX* and *GbY*, were included for comparison. All six GbE genes were essentially restricted to the ovary transcriptome. The RPKM (Reads Per Kilobase exon model per Million reads) values were very high and reached 6,364.7 RPKM. In other tissues, the RPKM values were between 0 and 5 (Supplemental Information Table 3). The cumulative RPKM value of all six *GbE* genes in the ovaries was 19,942. For comparison: The cumulative RPKM of all five *Mb* genes was 562 in skeletal muscle and 2,017 in the heart (Supplemental Information Table 3). The total levels of Mb mRNA in ovaries amounted to 5.92 RPKM. To validate the ovary-specific expression of *GbE*, quantitative real-time RT-PCR was carried out with *L. paradoxa* ovaries and other selected tissues (Fig. 4). Again, *GbE1* mRNA was exclusively detected in the ovaries, but not in other tissues, including the eye.

We further analysed the transcriptomes of the West African lungfish *P. annectens*^33^. These transcriptomes included male and female gonads, along with brain and liver transcriptomes. The five *GbE* genes were found highly expressed in the female gonads, with cumulative RPKM of 31,916 to 37,275 (Supplemental Information Fig. 8; Supplemental Information Table 4). In the male gonads, expression of GbE was at least 1,800-fold lower with cumulative RPKM of 0.85 and 17.69. In other tissues, cumulative RPKMs of GbE were <4. Except in the gonads, there was no difference in GbE levels between other male and female organs. In *P. annectens*, we also found an Adgb cDNA, which is expressed at low levels mainly in the mature male gonads (Supplemental Information Fig. 8). RNA-Seq analyses of the three available transcriptomes of the marbled lungfish *P. aethiopicus* showed the same picture: High expression of *GbE* in the ovary (cumulative RPKM of the seven *GbEs*: 53,661), but only traces of *GbE* in the transcriptomes of the developing jaw/mandible or mixed visceral tissues (Supplemental Information Fig. 9; Supplemental Information Table 5).

We further identified GbE proteins in the ovaries of *L. paradoxa* and *P. aethiopicus* by mass spectrometry. After separation of ovary proteins by SDS-PAGE, prominent bands with the expected GbE mass of ~15 kDa were detected (Supplemental Information Fig. 10). Mass spectrometry identified in these bands the proteins LpaGbE1-2e in the *L. paradoxa* samples, and PaeGbE1a, b, c, and PaeGbE2 a, b in the *P. aethiopicus* samples, respectively (Supplemental Information Fig. 2).

To check whether the oocyte-specific expression was overlooked in previous studies with birds^20,21^, we evaluated the ovary- and egg-specific transcriptomes of chicken (*Gallus gallus*) (Supplemental Information Table 1). In none of these transcriptomes, *GbE* sequences were found (Supplemental Information Fig. 11; Supplemental Information Table 6). We randomly checked further ovary transcriptomes of other vertebrate species, as available at SRA, for putative GbE sequences via BLAST. We included all vertebrate classes, but in none of these datasets, GbE sequences were detected. A comprehensive BLAST search of the available transcriptomes (including the transcriptome shotgun assemblies; TSA) or genomes at Genbank or ENSEMBL identified GbE only of birds, turtles, and the coelacanth.

### Localisation of *GbE* mRNA in previtellogenic oocytes

**Figure 5:**
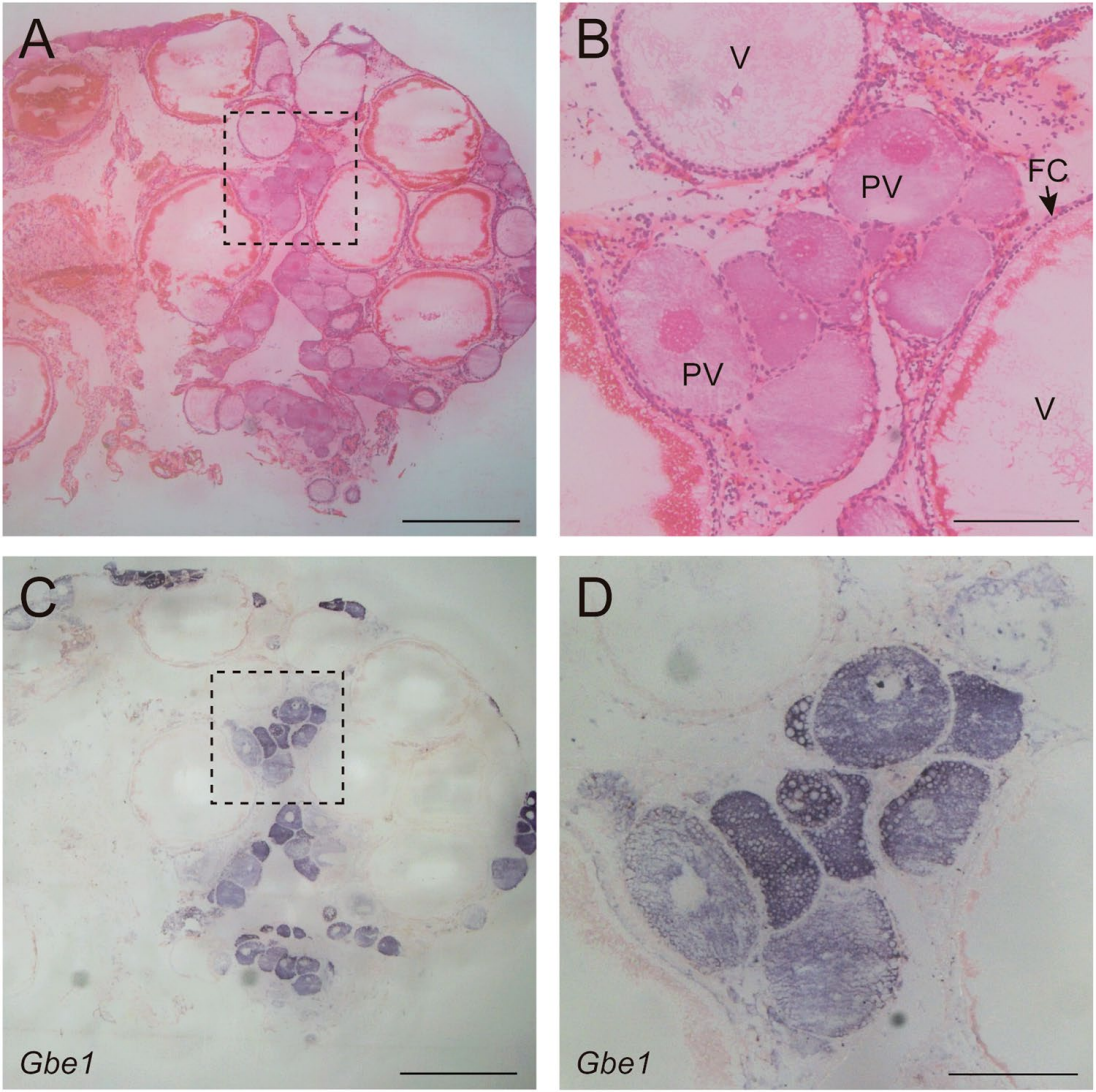
*GbE1* expression in the ovary of *L. paradoxa*. (**A**,**B**) Haematoxylin and eosin stained in ovary sections, oocytes at various stages of maturation can be seen. (**C**,**D**) Antisense *GbE1* riboprobe signal was detected in previtellogenic oocytes. (**B**,**D**) Represent zoom in of a dashed line box in (**A**,**C**) respectively. (**B**) Arrow indicates follicular cells. Previtellogenic oocytes (PV); Vitellogenic oocyte (V); follicular cell (FC). Scale bar: 2 mm (**A**,**C**) and 0.5 mm (**B**,**D**). The sense controls are given in Supplemental Information Fig. 12.

To determine the spatial expression on *GbE1* of *L. paradoxa* (*LpaGbE1*) we performed *in situ* hybridisations in adult gonads of a female South American lungfish. Hematoxylin-eosin stained sections of the ovary show oocytes in various stages of maturation (Fig. 5A,B). Oocyte development in *L. paradoxa* consists of an initial stage of previtellogenic oocytes, characterised by a basophilic cytoplasm. Next continuous yolk deposition results in a rapid increase in cellular volume in vitellogenic oocytes. Flattened follicular cells surround the vitellogenic oocyte, and acidophilus yolk granules cortically localized gradually expand into the cytoplasm. Mature oocytes measure about 2 mm^34^. *LpaGbE1* antisense probe signal was observed specifically in the cytoplasm of previtellogenic basophilic oocytes (Fig. 5C). *LpaGbE1* expression was not detected in vitellogenic oocytes, follicular cells or other ovarian cells (Fig. 5C,D). No signal was detected by the sense control probe (Supplemental Information Fig. 12).

### Spectroscopic studies and O_2_ binding equilibria to GbE1 of *L. paradoxa*

**Figure 6:**
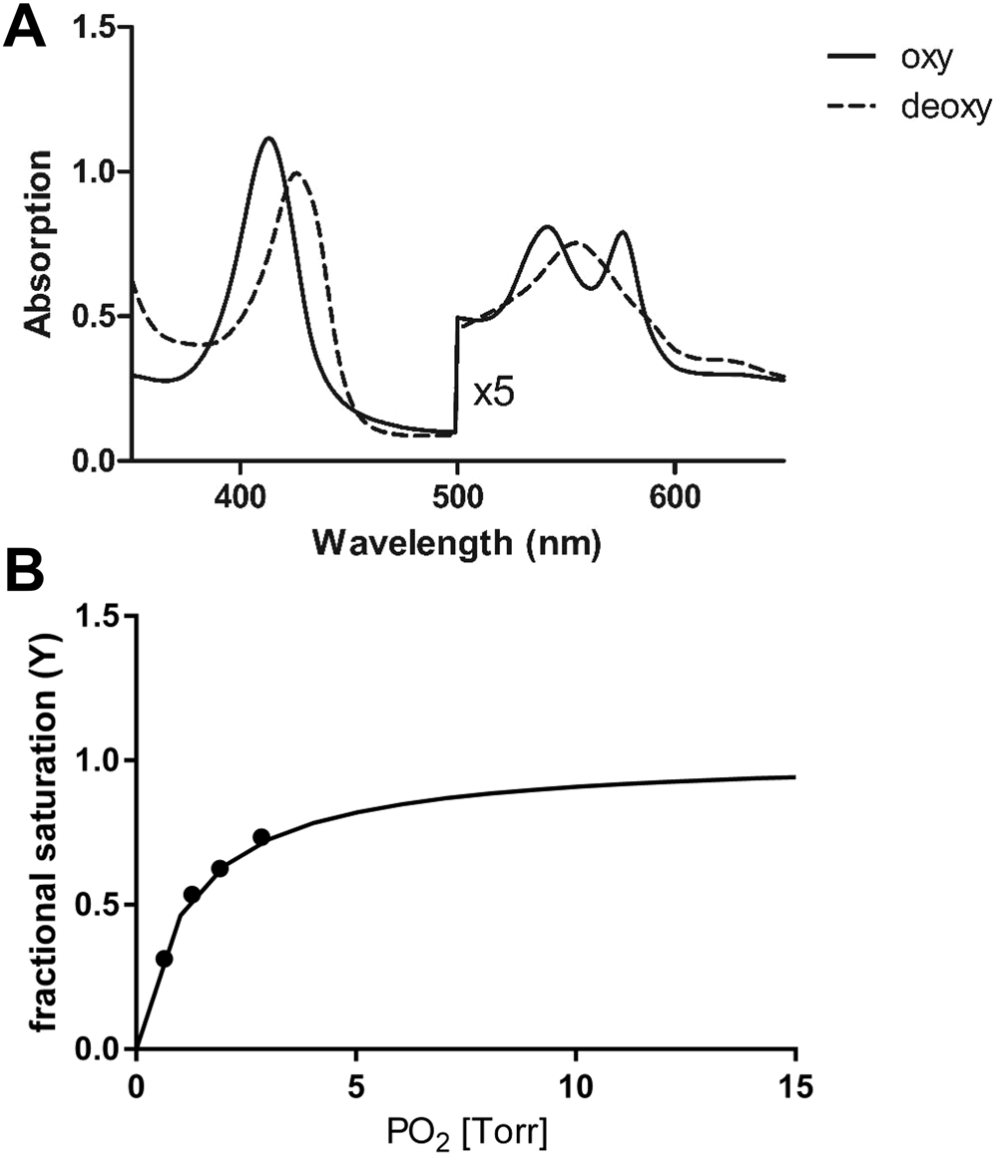
Heme coordination and O_2_ equilibria of recombinant GbE1 of *L. paradoxa*. (**A**) Absorbance spectra of purified (solid line) and deoxygenated (dotted line) recombinant GbE1 after addition of dithionite, indicating penta coordinate heme. (**B**) Representative O_2_ equilibrium curve, measured at pH 7.2, 20 °C. Fitting of saturation data is indicated (continuous line).

In size-exclusion chromatography, recombinant LpaGbE1 elutes largely as a monomer (data not shown). The absorbance spectrum of purified LpaGbE1 displayed a Soret band at 406 nm, an α band at 533 nm and a β-band at 579 nm (Fig. 6A), indicating a mixture between ferric and ferrous oxy forms. For comparison, a pure Mb oxy spectrum displays peaks at 418, 543 and 581 nm^35^. After reduction with Na-dithionite under nitrogen, the ferrous deoxy-form was obtained, with a large amplitude of the Soret band (427 nm) and a single peak in the visible region (555 nm). The absorption spectrum of deoxy-LpaGbE1 resembled that of Mb and Hb, indicating a pentacoordinate heme. O_2_ equilibrium curves (pH 7.2, 20 °C) showed that LpaGbE1 reversibly binds O_2_ (Fig. 6B), with a P_50_ of 1.2 ± 0.02 torr (0.16 kPa; 1 torr = 0.133 kPa). The O_2_ binding curve showed some degree of cooperativity (n = 1.19 ± 0.19), suggesting a dimeric assembly. The autoxidation rate of LpaGbE1 (pH 7.13, 20 °C), measured after removing the met reductase enzymatic system by gel filtration, was low (0.004 ± 0.0003 s^−1^). The nitrate reductase activity (pH 7.13, 20 °C) of deoxy LpaGbE1 was 18.72 ± 0.0022 s^−1^M^−1^.

## Discussion

RNA-seq and qRT-PCR studies showed that GbE is almost exclusively expressed in the lungfish ovary (Figs 3 and 4). More detailed studies by *in situ* hybridisation found that *LpaGbE1* mRNA is restricted to non-mature previtellogenic oocytes, but was not found in other cells of the ovary, such as follicular cells, and was also not detected in vitellogenic oocytes (Fig. 5). The previtellogenic oocytes have not yet commenced accumulating yolk and other material. Thus, *GbE* mRNA is massively deposited in the oocyte and, as suggested by the mass spectrometric data, translated into GbE protein (Supplemental Information Fig. 10). Here, GbE may carry out its respiratory function either in the oocyte or may be used to support embryonic development.

After external fertilisation, males provide parental care to the fertilised eggs in underground burrows^36^. Stagnant waters where *Lepidosiren* nests have been found are characterised by low oxygen levels, ranging from 0.2 to 1 cm^3^ per litre of dissolved O_2_^37^. Fertilized *Lepidosiren* eggs can reach 7 mm in diameter and, given the low oxygen conditions of the nests, it was unclear how *Lepidosiren* eggs obtained sufficient oxygen supply to sustain development. *Lepidosiren* males develop pelvic fin filaments during the breeding season, which break off and degenerate after larvae hatch and leave the nest^36^. Initially, it was suggested that these filaments could contribute to copulation as spawning brushes to spread seminal fluid. However, given the overall similarity between pelvic fin filaments and external gills, it was also proposed that pelvic fin filaments were used to aerate the eggs by releasing oxygen from the male’s blood into the water^38^. Recently, however, it was shown that *Lepidosiren* pelvic fin filaments neither have the morphology compatible for oxygen diffusion nor the gene expression profile typical of gill filaments^39^. Therefore, it is conceivable that GbE may help to extract O_2_ from the water to support the development of the embryo, which may be considered analogous to the function of the embryonic Hb in tetrapods^3^.

In contrast to the lungfish Mb genes, which functionally diversified after duplication^32^, the multiple *GbE* genes most likely encode proteins with similar functions. The presence of multiple palogous *GbE* genes thus enhance *GbE* mRNA levels, as evident by the very high RPKM values between 20,000 and 63,000 (depending on the species). These values exceed the RPKM of Mb in the muscle or heart (Fig. 3; Supplemental Information Figs 5–7; Supplemental Information Table 3) and are even higher than those of *Hb* in blood. Although we do not know the protein levels, the data suggest that GbE has an Mb-like role and contributes to the O_2_ supply of the oocytes. This hypothesis is supported by the notable GbE protein bands in the Coomassie-stained gels (Supplemental Information Fig. 10), and by the O_2_-binding equilibrium curve, with a P_50_ similar to that of a typical vertebrate Mb^40^, as well as by the low autoxidation rate of GbE. In addition, deoxy LpaGbE1 shows nitrite reductase activity similar to that of vertebrate Mbs^40^, indicating that GbE may also contribute to nitrite-dependent NO generation and signalling pathways in the ovary during periods of hypoxia. Other functions of GbE, for example as a vitellogenin-like storage protein, cannot be excluded but are less likely.

The globin family is a classic example of subfunctionalisation and neofunctionalisation of genes after duplication^41^. This notion is supported by the existence of eight distinct globin genes in vertebrates, which emerged early in evolution^1^. More recent and lineage-specific amplification events of members of the globin family are mirrored by the multiple *Mb*^32^ and *GbE* genes (this study) of lungfish, which is unusual among vertebrates. The phylogenetic tree (Fig. 2) showed that amplification of GbE genes commenced already in the lungfish stem lineage (*i.e*., separation of the GbE1 and GbE2 clades), but then occurred independently within the orders Lepidosiren and Protopterus. Lungfish are well-known for their gigantic genomes, which may, in theory, also result in the amplification of gene numbers. This explanation is unlikely for the globin genes. We only observed multiple copies of *Mb* and *GbE*, but of no other globin gene. In fact, lungfish have lost *Ngb* and *Cygb* genes. It is possible that their functions have been taken over by *Mb* copies^32^. The *GbE* genes seem to have an oocyte-specific function in lungfish, which is – to the best of our knowledge – not mirrored by any other globin-type in another vertebrate.

Although additive effects of the expression of multiple *Mb* genes in lungfish may be important to increase O_2_ supply, it is more likely that the different Mbs carry out distinct functions. This hypothesis is supported by the distinct expression patterns of the *P. annectens* Mbs, as well as by their different abilities to transfer stress tolerance^32^ and O_2_ binding properties (J. Lüdemann, A. Fago, T. Burmester, unpublished). By contrast, we propose that the multiple *GbE* genes have additive functions. This is evident by their expression in a single tissue, the ovary, as well as by the high similarity of the sequences. While Mb amino acid sequences may differ up to 70%, the maximum divergence of the lungfish GbEs is 40%. We further propose that lungfish GbE has a similar function in the ovary as Mb in the heart and skeletal muscle and may thus provide additional O_2_ either by enhancing O_2_ storage or by facilitating intracellular O_2_ diffusion.

**Figure 7:**
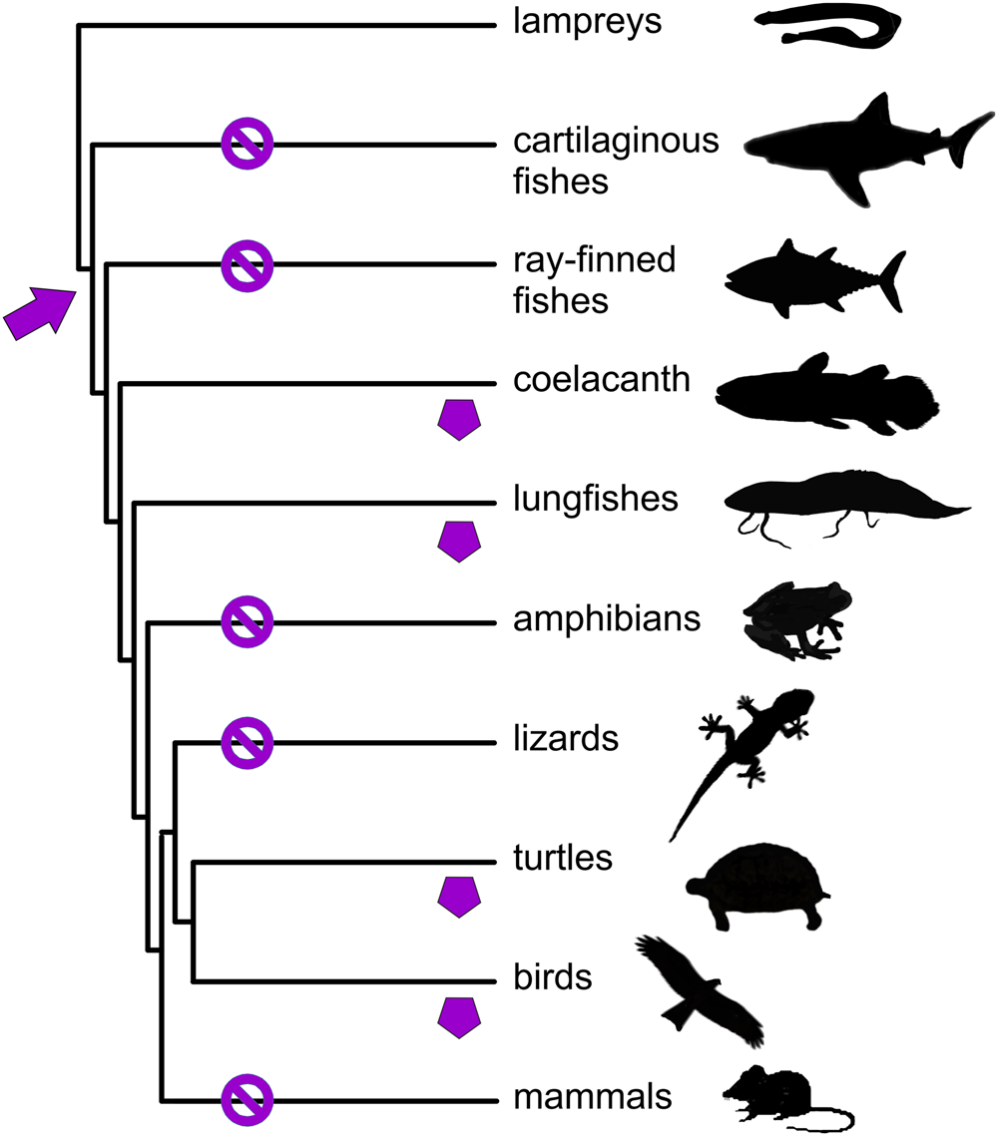
Occurrence of GbE in vertebrates. GbE (purple pentagon) genes are present in the genomes of the coelacanth, lungfishes, turtles, and birds, but has been lost in other vertebrate taxa (indicated by ∅). The arrow indicates the origin of GbE, *i.e*., its divergence from Mb. Genomes were searched by BLAST at ENSEMBL (https://www.ensembl.org/) and NCBI (https://www.ncbi.nlm.nih.gov/genome/).

There is little doubt that the globins originated from a single ancestor, and multiple gene duplications have led to their functional diversification^1,22,26^. Phylogenetic analyses suggest that all eight globin types were present in the gnathostome ancestor and that subsequent losses in certain clades have shaped the present globin repertoire of the vertebrate taxa. Globin E has been lost in all vertebrate clades except birds, turtles, lungfish, and coelacanth (Fig. 7). Although database searches did not reveal any other GbE sequences, we cannot rule out that in future additional GbE genes will be discovered that are expressed at unexpected sites.

The present data suggest that GbE has a similar role as Mb in cellular O_2_ supply. Mb is still considered to exert its function mainly in muscle, but at least in non-tetrapods it displays a more widespread expression pattern in many tissues^19,42–44^. GbE and Mb share an ancient common ancestry (Fig. 7). It is possible that GbE originated as a variant of Mb that meets the particular needs (for example, regarding O_2_ affinity) for a specific tissue. It remains unknown whether the original function was in the oocyte (like in lungfish) or the eye (like in sauropsids). In future, the coelacanth may be helpful to decide on the direction of evolutionary change but none the currently available transcriptomes of this taxon derives from eye or ovary. However, it is evident that either in the lungfish or the sauropsid clade, GbE must have changed its expression site. For unknown reasons, GbE was independently lost in at least five vertebrate clades^23^ (Fig. 7).

## Methods

### Lungfish material

A *L. paradoxa* specimen was collected near Breves, Brazil, and euthanised with a lethal dose of tricaine methanesulfonate. This study was approved by IBAMA/SISBIO under license number 47206-1, and experimental procedures and animal care were conducted in accordance to the Ethics Committee for Animal Research at the Universidade Federal do Pará, under the approved protocol number 037-2015.

West African lungfish (*P. annectens*) and marbled lungfish (*P. aethiopicus*) were obtained from a pet shop, euthanised in 1 g/l tricaine methanesulfonate and finally killed by decapitation. Tissues were removed and immediately stored frozen at −80 °C in RNAlater (Qiagen, Hilden, Germany). Animals were treated in accordance with the German Animal Welfare Act.

### RNA extraction, library preparation and Illumina sequencing

Total RNA was purified from lung, brain, buffy coat and ovary of South American Lungfish (*L. paradoxa*) and extracted for transcriptome using TRIzol^®^ Reagent (Life Technologies, Cat. 15596-026) according to the manufacturer’s protocol. RNA samples were further purified using RNeasy^®^ Mini Kit (Qiagen) and treated with DNaseI (Qiagen), according to the manufacturer’s protocol. Reference transcriptome and transcript abundance estimation were obtained from each library, sequenced on an Illumina Hiseq platform with 100 bp paired-end reads. Sequencing was carried out by a commercial service from Instituto Nacional do Câncer, Brazil.

RNA was purified from the ovary of the marbled lungfish (*P. aethiopicus*) using the CRYSTAL RNA Mini Kit (biolab products). A library for paired-end sequencing was generated from ~1 µg total RNA. Sequencing of 2 × 150 nt was performed by with the Illumina NextSeq500 technology (StarSEQ, Mainz, Germany).

Additional transcriptomes from various lungfish and other species were retrieved from the public SRA database at GenBank (for accession numbers, see Supplemental Information Table 1). The transcriptomes from each lungfish species were assembled using either the CLC Genomics Workbench (version 11.0.1) or Trinity v2.6.5. Globin cDNA sequences were identified in the assemblies employing TBLASTN using the coelacanth globins^24^ as queries. The final assignment of a globin to a specific clade was done by phylogenetic analyses (see below). The consensus sequences were verified using a backmapping approach using the CLC Genomics Workbench. Broken mate-pairs were employed to identify putative misassemblies. If required, the reads were re-assembled with different parameters, and the procedure was repeated until unambiguous *GbE* cDNA sequences were obtained. Selected GbE sequences were verified by RT-PCR and Sanger sequencing of the cDNA (GATC, Konstanz, Germany). RNA-Seq analyses were performed with the CLC Genomics Workbench. The mRNA levels of the globins were calculated as RPKM.

### Sequence analyses and phylogenetic inference

The vertebrate genomes available at ENSEMBL (https://www.ensembl.org/) and NCBI (https://www.ncbi.nlm.nih.gov/genome/) were searched for the presence of *GbE* genes. The lungfish GbE amino sequences were included in an alignment that covers the whole range of vertebrate globins and a broad range of classes^25,32,45^ (Supplemental Information Table 2). Adgb was excluded from the phylogenetic analyses because of its permutated globin domain^13^. A multiple sequence alignment of the amino acid sequences was obtained with the MAFFT online tool with the L-INS-i method^46,47^. Phylogenetic analysis was done with MrBayes 3.2.3^48,49^ using the LG model of amino acid evolution^50^, which was selected using PROTTEST^51^, and which was implemented into MrBayes with the general time reversible model as fixed prior and by specifying the aarevmatpr and statefreqpr options^24,25^. The program was run for 5,000,000 generations using the standard option (two independent runs with four simultaneous chains). Trees were sampled every 1000^th^ generation, and the posterior probabilities were estimated after discarding the initial 25% of the trees.

### Quantitative real-time reverse-transcription PCR

Reverse transcription was performed with 1 µg of total RNA, oligo-(dT)18 oligonucleotides (10 µM) and 200 U SuperScript^TM^ II RNase H^−^ Reverse Transcriptase (Invitrogen) according to the manufacturer’s protocol. Quantitative real-time RT-PCR (qRT-PCR) experiments were carried out on an ABI 7500 Real-Time PCR system (Applied Biosystems, Darmstadt, Germany) with the “ABI Power SYBR Green Master Mix”. The efficiency of the reaction was measured by the slope of a standard curve, deriving from tenfold dilutions of plasmids. Expression data were normalised according to the amount of total RNA. Further analyses were carried out employing the Microsoft Office Excel spreadsheet program.

### *In situ* hybridisation

Ovary tissue was extracted from *L. paradoxa* and embedded in Tissue Tek O.C.T compound (Sakura Finetek) in dry ice, and then stored in −80 °C freezer for subsequent cryosectioning. Then, 20 µm sections were produced on ColorFrost Plus microscope slides (Thermo Fisher Scientific) on a cryostat at −20 °C. Sections were fixed according to a previously established protocol^52^. After drying at room temperature, slides were stored in −80 °C ultrafreezer. Hematoxylin (Sigma-Aldrich) and eosin (Sigma-Aldrich) staining were performed according to standard protocol. A pGEM-T vector (Promega, Mannheim, Germany) containing *GbE1* of *L. paradoxa* served as a template in PCR amplification using forward and reverse M13 primers (reverse: 5′-CAGGAAACAGCTATGAC-3′; forward: 5′-GTAAAACGACGGCCAG-3′). PCR was performed in 50 µl reaction volumes containing 39.2 µl of RNase free water, 1.5 µL of 10 mM MgCL_2_, 5 µL of 10x buffer, 1 µL of each primer (0.5 M), 1 µL of dNTP mix (10 mM), 0.3 µL of Taq DNA Polymerase, and 1 µL of DNA template. The temperature profile consisted of preheating at 94 °C for 3 min, 32 cycles of denaturation at 94 °C for 45 s, annealing at 56 °C for 30 s, and extension at 72 °C for 90 s, followed by a final extension step at 72 °C for 10 min. Sense and antisense riboprobes were synthesised using T7 RNA and Sp6 RNA polymerases, respectively, and DIG-labelling mix (Roche). The riboprobe reaction (Life Technologies) was performed in 20 µL reaction volumes containing 0.5 µL of RNase inhibitor, 2 µL of DTT (0.01 M), 2 µL of DIG, 2 µL of 10x reaction buffer, 5 µL of template, 2 µL of Sp6/T7 enzyme mix, and 6.5 µL of nuclease-free water. *In situ* hybridisation was performed according to a previously established protocol^53^, using 300 ng/slide of DIG-labelled riboprobe. Slides were imaged using a Nikon SMZ1500 microscope (Nikon Digital Sight DS-Ri1).

### Identification of GbE proteins by mass spectrometry

Total proteins were isolated from the ovaries of *L. paradoxa* and *P. aethiopicus*. The tissues were homogenised in 10 mM Hepes-buffer with a Minilys Personal Homogeniser (Bertin Instruments, Bretonneux, France) 3 times for 30 s. Then, DNA and RNA were destroyed by sonification (Bandelin Sonopuls, Berlin, Germany). After centrifugation at maximum speed (13,000 × g, 10 min, 4 °C), the supernatant was collected. Total protein from each ovary was separated on a 15% SDS-polyacrylamide gel. The gel was stained with Coomassie brilliant blue. Putative GbE bands were excised and analysed by liquid chromatography-mass spectrometry using a commercial service (Core Facility Mass Spectrometric Proteomics, University Medical Center Hamburg-Eppendorf, Germany).

### Preparation of recombinant lungfish GbE protein

The coding sequence of *GbE1* of *L. paradoxa* was amplified by RT-PCR and then cloned into the pET16b expression vector (Novagen - Merck Biosciences, Darmstadt, Germany). Recombinant expression was done in *E. coli* BL21(DE3)pLysS cells (Promega, Mannheim, Germany), which were grown at 37 °C in 5 ml L-medium (1% bactotryptone, 0.5% yeast extract, 0.5% NaCl, pH 7.5) containing 10 µg/ml ampicillin, 34 µg/ml chloramphenicol over night. The culture was applied to 500 ml L-medium supplemented with 1 mM δ-aminolevulinic acid. The culture was induced at OD_600_ = 0.4 to 0.8 by the addition of isopropyl-1 thio-D-galactopyranoside (final concentration 0.4 mM), and expression was continued at 30 °C overnight. Cells were collected by 45 min centrifugation at 4,000 g and resuspended in 50 mM Tris-HCl, pH 8.0, 1 mM MgCl_2_, 1 mM dithiothreitol, 10 μg/ml DNase, 5 µg/ml RNase, Complete^TM^ proteinase inhibitor mix (Roche Applied Science) and Pefabloc (Roth). The cells were broken by three freeze-thaw cycles in liquid nitrogen followed by ultrasonication (10 × 30 s). DNA and RNA were digested for 2 h at 37 °C. The cell debris was removed by centrifugation for 1 h at 4 °C at 4,500 × g. The recombinant GbE1 was purified from the supernatant using His60-Ni columns (Qiagen) according to the manufacturer’s instructions. The His-tag was removed by incubation with the Factor Xa protease (20 µg/ml) for 6 h at 37 °C. After inactivation of the protease with 2 µM dansyl-glu-gly-arg-chloromethyl ketone, the recombinant GbE1 protein was brought to 10 mM Hepes, pH 7.8, 0.5 mM EDTA.

Gel-filtration was carried out with an ÄktaPure chromatographic system (GE Healthcare, Freiburg, Germany) equipped with a Superdex 75 10/300 column (GE Healthcare). The proteins were eluted with 50 mM potassium phosphate, 0.5 mM EDTA, pH 7.0, 0.15 M NaCl at a flow rate of 1 mL/min. The absorbance was measured at 280 and 415 nm. Recombinant GbE1 was applied at a concentration of 0.2 mM heme; Human Hb and horse Mb were employed as references at the same concentration.

### Spectroscopic studies and O_2_ binding curves

Absorbance spectra of purified GbE1 were taken in the range 350–650 nm. The deoxy-form was obtained by adding sodium dithionite. The determination of O_2_ equilibrium curves was done using a modified diffusion chamber technique previously described^54–56^. Samples were measured in at least duplicates of 5 µL (~0.2 mM heme) in 0.1 M Hepes, 0.5 mM EDTA, pH 7.2 at 20 °C. Ferric GbE1 was reduced for 5 to 10 min in N_2_ with the met-Hb reductase system^57^.

In the modified diffusion chamber technique, water-saturated gas mixtures of O_2_ and ultrapure (>99.998%) N_2_ were generated by GMS 500 gas mixing system (Loligo System, Denmark) and used to equilibrate the thin smear of 5 µL GbE1 sample with stepwise increments of oxygen tension (P_O2_). Absorbance traces were sampled at 436 nm by a photomultiplier (model RCA 931-A) and an Eppendorf model 1100 M photometer. The signal was digitalised, and saturation values were obtained using an in-house software^58^. P_50_ and cooperativity values were calculated from the zero intercept and slope of Hill plots, respectively. Each curve consisted of four to six saturation steps.

### Nitrite reductase activity

The reaction of recombinant deoxy GbE1 (10 µM heme in deoxygenated 50 mM HEPES, pH 7.13) with nitrite (0.1 mM) was carried out under pseudo-first-order conditions at 20 °C^59,60^. Before adding nitrite, the GbE1 was anaerobically titrated with 1 mM of sodium dithionite in a 1 cm cuvette sealed with a rubber cap. The measurement of the reaction kinetics was started immediately, and absorbance traces were recorded at 435 nm and 20 °C.

## Electronic supplementary material

Supplemental Information

## References

[1] Burmester T, Hankeln T (2014). Function and evolution of vertebrate globins. Acta Physiol. (Oxf.).

[2] Weber RE, Vinogradov SN (2001). Nonvertebrate hemoglobins: Functions and molecular adaptations. Physiol. Rev..

[3] Dickerson, R.E. & Geis, I. *Hemoglobin: structure, function, evolution, and pathology*, (Benjamin/Cummings Pub. Co., 1983).

[4] Wittenberg JB, Wittenberg BA (2003). Myoglobin function reassessed. J. Exp. Biol..

[5] Burmester T, Weich B, Reinhardt S, Hankeln T (2000). A vertebrate globin expressed in the brain. Nature.

[6] Burmester T, Hankeln T (2009). What is the function of neuroglobin?. J. Exp. Biol..

[7] Hankeln T (2005). Neuroglobin and cytoglobin in search of their role in the vertebrate globin family. J. Inorg. Biochem..

[8] Bentmann A (2005). Divergent distribution in vascular and avascular mammalian retinae links neuroglobin to cellular respiration. J. Biol. Chem..

[9] Mitz SA (2009). When the brain goes diving: glial oxidative metabolism may confer hypoxia tolerance to the seal brain. Neuroscience.

[10] Burmester T, Ebner B, Weich B, Hankeln T (2002). Cytoglobin: a novel globin type ubiquitously expressed in vertebrate tissues. Mol Biol Evol.

[11] Nakatani K (2004). Cytoglobin/STAP, its unique localization in splanchnic fibroblast-like cells and function in organ fibrogenesis. Lab. Invest..

[12] Schmidt M (2004). Cytoglobin is a respiratory protein in connective tissue and neurons, which is up-regulated by hypoxia. J. Biol. Chem..

[13] Hoogewijs D (2012). Androglobin: a chimeric globin in metazoans that is preferentially expressed in Mammalian testes. Mol. Biol. Evol..

[14] Fuchs C, Burmester T, Hankeln T (2006). The amphibian globin gene repertoire as revealed by the *Xenopus* genome. Cytogenet. Genome Res..

[15] Roesner A, Fuchs C, Hankeln T, Burmester T (2005). A globin gene of ancient evolutionary origin in lower vertebrates: evidence for two distinct globin families in animals. Mol. Biol. Evol..

[16] Blank M, Burmester T (2012). Widespread occurrence of N-terminal acylation in animal globins and possible origin of respiratory globins from a membrane-bound ancestor. Mol. Biol. Evol..

[17] Blank M (2011). A membrane-bound vertebrate globin. PLoS ONE.

[18] Koch J, Burmester T (2016). Membrane-bound globin X protects the cell from reactive oxygen species. Biochem. Biophys. Res. Commun..

[19] Gallagher MD, Macqueen DJ (2017). Evolution and expression of tissue globins in ray-finned fishes. Genome Biol Evol.

[20] Kugelstadt D, Haberkamp M, Hankeln T, Burmester T (2004). Neuroglobin, cytoglobin, and a novel, eye-specific globin from chicken. Biochem. Biophys. Res. Commun..

[21] Blank M (2011). Oxygen supply from the bird’s eye perspective: Globin E is a respiratory protein in the chicken retina. J. Biol. Chem..

[22] Storz JF, Opazo JC, Hoffmann FG (2011). Phylogenetic diversification of the globin gene superfamily in chordates. IUBMB Life.

[23] Hoffmann FG, Opazo JC, Storz JF (2011). Differential loss and retention of cytoglobin, myoglobin, and globin-E during the radiation of vertebrates. Genome Biol. Evol..

[24] Schwarze K, Burmester T (2013). Conservation of globin genes in the “living fossil” *Latimeria chalumnae* and reconstruction of the evolution of the vertebrate globin family. Biochim. Biophys. Acta.

[25] Schwarze K, Singh A, Burmester T (2015). The full globin repertoire of turtles provides insights into vertebrate globin evolution and functions. Genome Biol. Evol..

[26] Storz JF, Opazo JC, Hoffmann FG (2013). Gene duplication, genome duplication, and the functional diversification of vertebrate globins. Mol. Phylogen. Evol..

[27] Amemiya CT (2013). The African coelacanth genome provides insights into tetrapod evolution. Nature.

[28] Irisarri I, Meyer A (2016). The identification of the closest living relative(s) of tetrapods: Phylogenomic lessons for resolving short ancient internodes. Syst Biol.

[29] Takezaki N, Nishihara H (2017). Support for lungfish as the closest relative of tetrapods by using slowly evolving ray-finned fish as the outgroup. Genome Biol Evol.

[30] Irisarri I (2017). Phylotranscriptomic consolidation of the jawed vertebrate timetree. Nat Ecol Evol.

[31] Jørgensen, J. M. & Joss, J. *The Biology of Lungfishe*s, (Science Publishers, Enfield, NewHampshire, 2010).

[32] Koch J (2016). Unusual Diversity of Myoglobin Genes in the Lungfish. Mol Biol Evol.

[33] Biscotti MA (2016). The Lungfish Transcriptome: A Glimpse into Molecular Evolution Events at the Transition from Water to Land. Sci Rep.

[34] Chaves PTC (1992). Some aspects of the oogenesis in the South american lungfish, *Lepidosiren paradoxa* Fitzinger (Dipnoi). Rev. Bras. Zool..

[35] Antonini E, Brunori M (1970). Hemoglobin. Annu Rev Biochem.

[36] Kerr JGV (1900). The the external features the development of *Lepidosiren paradoxa*, Fitz. Proc. R. Soc. Lond..

[37] Carter GS, Beadle LC (1931). Reports of an Expedition to Brazil and Paraguay in 1926–7, supported by the Trustees of the Percy Sladen Memorial Fund and by the Executive Committee of the Carnegie Trust for the Universities of Scotland. The Fauna of the Swamps of the Paraguayan Chaco in relation to its Environment.—II. Respiratory Adaptations in the Fishes. Zool. J. Linn. Soc..

[38] Cunningham JT (1929). The vascular filaments on the pelvic limbs of *Lepidosiren*, their function and evolutionary significance. Proc. R. Soc. Lond..

[39] Lima SQ, Costa CM, Amemiya CT, Schneider I (2017). Morphological And Molecular Analyses of an Anatomical Novelty: The Pelvic Fin Filaments of the South American Lungfish. J Exp Zool B Mol Dev Evol.

[40] Fago A, Jensen FB (2015). Hypoxia tolerance, nitric oxide, and nitrite: lessons from extreme animals. Physiology (Bethesda).

[41] Ohno, S. *Evolution by gene duplication*, (Springer-Verlag, 1970).

[42] Cossins AR, Williams DR, Foulkes NS, Berenbrink M, Kipar A (2009). Diverse cell-specific expression of myoglobin isoforms in brain, kidney, gill and liver of the hypoxia-tolerant carp and zebrafish. J. Exp. Biol..

[43] Fraser J (2006). Hypoxia-inducible myoglobin expression in nonmuscle tissues. Proc. Natl. Acad. Sci. USA.

[44] Opazo JC (2015). Ancient duplications and expression divergence in the globin gene superfamily of vertebrates: Insights from the elephant shark genome and transcriptome. Mol. Biol. Evol..

[45] Schwarze K (2014). The globin gene repertoire of lampreys: convergent evolution of hemoglobin and myoglobin in jawed and jawless vertebrates. Mol. Biol. Evol..

[46] Katoh K, Asimenos G, Toh H (2009). Multiple alignment of DNA sequences with MAFFT. Methods Mol Biol.

[47] Katoh K, Toh H (2008). Recent developments in the MAFFT multiple sequence alignment program. Brief. Bioinform..

[48] Huelsenbeck JP, Ronquist F (2001). MRBAYES: Bayesian inference of phylogenetic trees. Bioinformatics.

[49] Ayres DL (2012). BEAGLE: an application programming interface and high-performance computing library for statistical phylogenetics. Syst. Biol..

[50] Le SQ, Gascuel O (2008). An improved general amino acid replacement matrix. Mol. Biol. Evol..

[51] Darriba D, Taboada G, Doallo R, Posada D (2011). ProtTest 3: fast selection of best-fit models of protein evolution. Bioinformatics.

[52] Nogueira AF (2016). Tetrapod limb and sarcopterygian fin regeneration share a core genetic programme. Nat Commun.

[53] de Lima JL (2015). A putative RA-like region in the brain of the scale-backed antbird, *Willisornis poecilinotus* (Furnariides, Suboscines, Passeriformes, Thamnophilidae). Genet Mol Biol.

[54] Weber RE (1981). Cationic control of O_2_ affinity in lugworm erythrocruorin. Nature.

[55] Weber RE (1992). Use of ionic and zwitterionic (Tris/BisTris and HEPES) buffers in studies on hemoglobin function. J. Appl. Physiol..

[56] Sick H, Gersonde K (1969). Method for continuous registration of O2-binding curves of hemoproteins by means of a diffusion chamber. Anal Biochem.

[57] Hayashi A, Suzuki T, Shin M (1973). An enzymic reduction system for metmyoglobin and methemoglobin, and its application to functional studies of oxygen carriers. Biochim. Biophys. Acta.

[58] Beedholm, K. Spektrosampler. (Aarhus University, Denmark, 2018).

[59] Fago A (2017). A comparison of blood nitric oxide metabolites and hemoglobin functional properties among diving mammals. Comp Biochem Physiol A Mol Integr Physiol.

[60] Fago A, Rohlfing K, Petersen EE, Jendroszek A, Burmester T (2018). Functional diversification of sea lamprey globins in evolution and development. Biochim Biophys Acta.

