# Supplementary material for: Globin E is a myoglobin-related, respiratory protein highly expressed in lungfish oocytes
Source: Sci Rep. 2019 Jan 22;9:280. doi: 10.1038/s41598-018-36592-w (PMC6343008; doi:10.1038/s41598-018-36592-w)
Supplement: Supplementary file 1 — Supplemental Information [file 41598_2018_36592_MOESM1_ESM.pdf]

Globin E is a myoglobin-related, respiratory protein highly expressed in lungfish oocytes

Julia Lüdemann[1], Kellen Matos Verissimo[2], Kimberley Dreger[1], Angela Fago[3], Igor Schneider[2] and Thorsten Burmester[1]

[1]Institute of Zoology, University of Hamburg, D-20146 Hamburg, Germany
[2]Instituto de Ciências Biológicas, Universidade Federal do Pará, Belém, PA, Brazil
[3]Department of Bioscience, Aarhus University, DK-8000 Aarhus C, Denmark

SUPPLEMENTAL INFORMATION

a. Sequences of *Lepidosiren paradoxa*

>LpaGbE1
ATGGCCATGTCTGCTGATGATATTCAGAAAGGAAGGTCTGCGTGGGAGAAGTTTTATGCAAATGCAGAGG
ATAATGGTGCAGTTGTGTTGAGCAGAATGTTCAAGGAACACCCCCACACTGTCAGTTACTTTAAAAACTT
CAAAGAATTGCAGTCCATGGCAGAGAAGGCCTCCTCTGTAGAACTTCAGGGCTTATCAGAAGTCCGAGGT
CATGGCAAGAAAGTATTGTCTGCTCTGAATGACATGGTACAGTATCTAGATAACATGGATTCTTTAAAAA
AAGTTACTGAACCTCTGGGCAAAAAACATGCTGTGGAACTTCAAGTGGATGTGAAGGATTTTGATATTAT
CTTTACAATTCTGCTGGACCTCCTGGGTGAAAAATGTGGTGGTGATGCCAAAACAGACTTGAAAAAAGTG
ACTGATCTTTTGTATGAGGAAATTAAGTCTACCTACTGA
>LpaGbE2a
ATGGCTCTTTCAGCTGAAGACATTCAGATGGTTAGTGGTGTGTGGAGTAAAATCTTTGCAGATGCTGAGT
CAAATGGTGCTGTTGTTTTAAGCAGAATGTTCAAGGAGTATCCACATACTGTGAAATATTTTAAGAACTT
CCCAGAGCTGCAGTCTATTGCTGAGACTGCTTCTGCTGCTGACATTGCAGGACTGGCTGAGGTTCGAGGC
CATGCAAAGACAGTACTTACAGCTTTTAATGATATGGTACAGCACTTGGAAAATATTGATACTTTAAAAG
AGACTGCTACACCTCTTGCCAAGAAGCATTCTGAAGAGCTTAAAGTAGATGTGAAGGACTTTAAAATCCT
CTGTGACAACCTGGTAGATCTCGTTGGAGAGAAGCAAGATGAAGATGCCAAAACCACCTTTAAGAAAGCA
GTGGATGTAATATATGAAAATATTTCAGCTGCATATTGA
>LpaGbE2b
ATGGCTCTTTCAGCTGAAGAAATTCAGAATGCTACTGGTGTGTGGAGTAAAATTTTTGCAGATGCCGAGT
CAAATGGTTCTGTGGTTTTAAGCAGAATGTTCAAGGAGTATCCACATACTGTGAAATATTTTAAAAACTT
CCCAGAGCTACAGTCTGTTGGTGAGACTGCTTCTGCTGCTGAGATTGCAGGACTGGCTGAGGTTCAAGGC
CATGCAAAGACCGTATTTACAGCTTTTAATGATATGGTACAGCACTTGGAAAATATTGATGCTTTAAAAG
AGACTGCTACTCCTCTAGCCAAGAAGCATTCTGAAGAGCTTAAAGTAGATGTGAAGGACTTTAAAATCCT
CTGTGACAACCTGGTAGATCTCGTTGGAGAGAAGCAAGATGAAGATGCCAAAAGCACCTTTAAGAAAGCA
GTGGATGAAATATATGAAAATATCAAAGCTGCATATTGA
>LpaGbE2c
ATGGCTTTTTCAGCAGAAGATATTCAGACTGCTACTGATGTTTGGGACAAAATCTTTGTAAATGCTGAAG
AGAATGGTGCTATTGTGTTAAGCAGAATGTTCAAGGAGCACAGTCATACTGTAAGCCATTTTAAAAACTT
CACAGAGTTGCAGTCTATTGCTGAGACTGGTTCTGCTGCAGAGATTGCAGCACTGGCTGAGGTTCAAGGG
CATGGGAAGAAAGTCTTTACAGCCTTGAATGATCTGGTTTTGAAGGTGGACAATGCAGATGCGTTAAAAG
AGGCTGTTGCTCCTCTAGCCAAGAAGCATGCTGAAGAGCTTAAAGTAGATGTGAAAGATTTGGGAGTCCT
CTGTGAAATCCTGATCGATCTTGTTGGAGAGAAGCAAGGTGATGCCAAAACAGCCTTTAAGAAAGTGATG
GATGTAATATATGAAAACATAAAAGCTGCATATTGA
>LpaGbE2d
ATGGCTCTTTCAGCAGAAGATATTCAGACTGCTACTGGTGTTTGGGACAAAATCTTTGTAAATGCTGAAG
AGAATGGTGCTATTGTGTTAAGCAGAATGTTCAAGGAGCACAGTCATACTGTAAGCCATTTTAAAAACTT
CACAGAGTTGCAGTCTATTGCTGAGACTGCTTCTGCTGCAGAGATTGCAGCACTGGCTGAGGTTCAAGGG
CATGGGAAGAAAGTCTTTACAGCCTTGAATGATCTGGTGCTGCATGTGGACAATGCAGATGCGTTAAACA
AGATTGCTGCTCCTCTAGCCAAGAAGCATGCTGAAGAGCTTAAAGTAGATGTGAAAGATTTGGGAGTCCT
CTGTGAAATCCTGATCGATCTTGTTGGAGAGAAGCAAGGTGAAGATGCCAAAACAGCCTTTAAGAAAGTG
ATGGATGTAATATATGAAAACCTAAAAGCTGCATATTGA
>LpaGbE2e
ATGGCTCTTTCAGCAGAAGATATTCAGACTGCTATTGGTGTTTGGGACAAAATCGTTGTAAATGCTGAAG
AGCATGGTGCTATTGTGTTAAGCAGAATGTTCAAGGAGCACAGTCATACTGTAAGCCATTTTAAAAACTT
CACAGAGTTGCAGTCTATTGCTGAGATTGCTTCTGCTGCAGAGATTGCAGGACTGGCTGAGGTTCGAGGG
CATGGGAAGAAAGTCTTTACAGCCTTGAATGATCTGGTGCTGCATGTGGACAATGCAGATGCTTTAAACA
AGATTGCTGCTCCTCTAGCCAAGAAGCATGCTGAAGAGCTTAAAGTTTGTGTTAAGGATCTGGGAGTCCT
CTGTGAAATCCTGATCGATCTTGTTGGAGAGAAGCAAGGTGATGCCAAAACAGCCTTTAAGAAAGTGATG
GATGTAATATATGAAAACATAAAAGCTGCATATTAA
>LpaGbX
TTCAAACAGTTGATAATGACAAATTTTTAACTTCAACTCCAGTTTACATTGACAAAAGAGACTACACCTG
TTAAAAAAAAAGAAAAAAAAGTAAGTGTTTTTCAGCTTCTCTGCATTTAATTAGATCCTGTCATTTGTTT
GTGATCTTTTGCACATTGCAATGGGCTGTGCTTTGTCAGAAGTTGGAAAAGAAGAGAATTCAGCAAGCAT
TGTGAATGAAATTTCTGGCGAAATAGGACCAGATACAGATATCGGATCAGGGACACAGTCTGGATCAATG
TCAAATCTTTCAACCCTTTCAGAATCCCAGAAGGAGCTTATTCAGGAATCATGGAAGATTCTTCATCAGG
ATATCACAAGAGTAGGAATCATAGTGTTCATACGCCTGTTTGAAACTCATCCAGAGTGTAAAGATGTATT
TTTCCTTTTTCGAGACATTGATGATGTTCAGCAGTTGAAGATAAGTAAGGAACTACAAGCACATGGTTTG
AGGGTGATGTCTTTTATTGAGAAGAGCGTTGCCAGATTACAGCAAGAGGACAAACTTCAACATATAGCCT
TTGAACTGGGTAGATGTCATTGCAGATACAATGCTCTGCCTAAATACTTTGAGTATGTAGGATTTCAGTT
TATGACTGCTGTGAAGCCCATTCTTAAAGAAAAATGGACATCAGAAGTAGAGGATGCATGGAAGAGTCTC

TTTAAATACTTGATTTCAATGATGAAGAAGGGTTTCCATGAAGAGGCAAAAAATCGTCTAAGCAACAAGT
CAACAGACCCAAAGAAACAGTTAACAACAAGAATGAAATTTGTTCAACATAATGTTTAATACAAAAAAGG
GTTAATAAAAAAAAAACATGATCTGAAATGAATGGGACATGTATTATGTATCTTTGGG
>LpaGbY
CATCCTGTTCAAGCACATTGTCAGCATGTCTTGTCTCAGCATGACTGATATTCAGAACATTAGAAACATC
TGGGGTATCATATGTCAGAATCCAGAGGCGAATGGGCAAATTGTTGTAACTAGACTTTTTGTAGATTATC
CACAGACCACACTATATTTCAAGAATTTTAAAAATCTTGATACTGTGGAAGGTATAAAAGAAAGCAAACA
AGTCCGAGAGCATGGTCGGAGAGTGGTAATGATGCTGGACAAGGTCATTGAGAATCTGGGTGACTGGAAT
GAGACCTCAAATCTTTTGTCCAAACTGGCAAAAAGACACCGGGATTATCACAACATACCAGTCAGGAACT
TCAAGTTCCTGTTTGCTTCACTCAACAGTGTTTACATTGATGTTTTTGGTCCAGCATTTACAGCAGATAT
CCAGACATCCTGGGAGAAGCTTTATTCACTGGTGTACCAAGAAATGGAAAAATGCTACAGCGCCATTCCT
TCATCATAGACTGTTACAAAGTGCTCCTTATATTGCAAGGGCAATACATATGCACTATTTTCTTTGTTTC
TTGGATTTGCATACTTTAATAGTACGCTAAGCTCACATTTTTAAGCAGTTTAAAAGCTGAAGTAGTATTA
TTCATGCTAAGTAATATTACATATAAAAAGTGTAAATAAGAATGTTAGAGAAAACAGAAGCTGTCAGTGT
TTTAACACATTATACAACTGTAAATACTACATAAACAAAAATATTGACAGAACACCTTAGTAATATTTAC
AAATCTTGGTCTGCTGTATTAACTTACCTGTCAGCCGGACTGTATTTTATTTCAGCACTGAGTAGAAAAC
GTATCTTGTACATTCTTGAAAAAAATGAAGACCAAAGTACATGGCAAAGGCTTCTTGTGTGCACAAAAAA
ACATTCCTCACAGACTTTTTCATATGGTTAAAAATTATGGCTTTCAGACTAAAGAAAGAGAGCCAAACCC
TTATCCAGTTTCTTGGATACATTTACATGCAAGTTGCTAATCCCTCATTAATCATTTCACTGAAACTAAC
ATATCATACAATAGCAATTTTCCTGATATTTATATTGCTGGCAACAAAGAAACTCTTTCTCAGTATATTT
CTTAAGTGGTAGTAGAGGAAATACTAAGCAGGAACTGGTTGAACCTCACTTCTACTATCTCCTTTTCTTT
TTCCACCAGTATTAAGAGAGTCCCATATACTGTGTTTTCAATTAAGACTTTTGCAAGTGGGCCCCTCCAG
TTGGTGCAGTGGAGCATTCCCTTG
>LpaHbA1
GATTGTCTGGTCTACACATCAGCTGAGACCAAGAATTTTAAAAATAAAAAAAAATCTAAATGGAATACAT
GAAAACAAAATGCCAAAAAAAATAAAATAAAATAAAAACAGATTTGTTTCCATTCATTGTTATGCAGGTG
ATGAAAAAATCTTTCAAGAATCCCAAGCTGGCTGTTTGGCTGTATGTCCAATAGTGAACGCACTTAGCGG
TATAAGGAGATGAGGACGTGAACTATTTGCCCAAGGCACTTTTCGGCAGCACAATTTATCTCAGGTGTGA
ATTTTTCTCCACAATGAGCAGCTATAGTAGTCATGATGGCTTCAATAAGGTACTGAAAGTTGCAAGGGTC
CACCATGAGTTCCCTGGCATGCTTTTCACTTAAAGTGTGCAAGCAACTTGACAGATCATGCAAATGTTGC
ACACCTTGCCCAATGGCATCCACCACCTTCTTCCCATGGTGCTTCACTTTTTCATTATTTGCTGAGAAAT
CATGTCCAAAGTGCGGGAAGTAGGACTTTGTACCAGGATAGCAACTAAACATTCTTGCTAAGGCTTCTCC
ACCTGCATTGGGTATCTGATGGAGTAATCCCCAGGCTTCTTTAATTAAGACTTCATCATCCTGACTGAAG
CGCATGCTTGCTATATTATGTGACAATCGCGACCCAGAGAAGAACAAGCACAGATGAT
>LpaHbA2
GGGTGTTCTGAATTATATATGAGGAAGTTCCTTTGTATGAATTTTTATTTCATCTTCATTTGTGATTTAG
GCATTTCGCTTATCTGTACTTTGATGTCAACACACGGCCAGTCAGTTGAAGATATTTGTCCCATGCAAGA
TGTAGTTCAGGAGTGAAGTCACCTTGCATGTGTGCAGCCAGGAGAACCAAAAAGCAATGAGAGATGTACG
GAAAGTTGGCAGGATCAACCTTGATGTTTTTGGCATGCAGGTCACTAAGGTCTGAGAGAGCAGCTGACAC
GTTATCGATATTCTTGATACCTTTACCAAGAGCAGCAATGATCTTTGCACCATGTGCACGGAGCTGGGCA
GACTTGGGGCTGGTGTCTACATTGGGAAAGTAGGTTCGGGTCTGGGGAAAGCTTGCAAACATTCTATTGA
AGAGTTCAGAACCATACTGCTCTGCATTAGGAGCAATCTTATCATAGCTGCTACTGAGAAGAGATTTTTC
AGCCTGGGTCAGAAGCATGGTTTCTCAAGGTATTTTCTT
>LpaHbB1
GGAGGAAGCCTTATCATGAATCGTCAGCCCTCCTGTGGTTGCAAACCGATTACTTGCCTACTATTTCTTA
AGACTGTCCACAAAACTCAGGCTGAATGTAAAAAAGCATAGAAGGTGTGATGTGAAGAGGATCCAGCTGT
TGCTGCAGATAAGGCAGGAAGGGGTTTTGTTCATTTTCTGCCTATATAAATTCATAAAAGCACAATAAAT
TTGGATAAATTCTTAAGAATAAAAAAGGCACCTCCCACATGCAAGCTTCCCCACCATGGTTCATTGGACA
AATGAGGAAGAATATGCCATCTTTTCTATATGGGGGGACATTGATGTTCATGAGGAAGGCCATCAGACTT
TGACAAGGCTGATGGTAGTGTATCCTTGGACAAAGAGGTACTTTTCTTCATTTGGAGACCTGAGCACCTC
CTGTTCTATAGCTGCGAATCACCTCGTAAAGGCCCATGGTGCGAAAGTCTTAACTGCCATTGGTGATGCT
CTCGTAGATATGCCCAACATCAAACACAACCTGACAGATCTCAGTCGCCTCCATTCAGAAATACTCCATG
TAGATCCAGAAAATTTTAGGCTTCTTGGCAAGTGTTTGTTAATTGTGCTGGCAGCCAAATTCGGTGCTAA
GAAGTTCAATGTTGATGTGCATGCTGCATGGAAAAAACTGATGGAGATCATTGCTGCAGGTCTATCCAAG
CAATATCATTAGGCCGAAGCAGTGCAGCGCATTCTTAAACAGAGTCAAGGAAAGAGTGACAATAAAATTC
CAAGGCATCATACACAGTAGTGCCCAGAGACTGGTACTGAAAAACAGGAGCAGCCAGAAATGGAGCTGTG
TATGAAATAAAAAAAATACAGAAATGCTACACTGTCTTTTATGGGGCAACAGAAATGCTACACTGTCTTT
TATGGGGCAACAGAAATGCTACACTGTCTTTTATGGGGCA
>LpaHbB2
AGTAAACTGTGCTCTGAACACAAACTTTGCTATCCATTTTAAGTGGGTGTGGTCAACCAATAGTTTTATC
AAATGATCAAATGTTTACCGCCCTCAGACAGCAATCATCTGAGAGATGATTAGCTGTTTTTGTGGGCCCC

ATTATCAGAGATGACTTCACTGGGTGGGAGCCGGCTTCTGCCCATATAAATCCCAACAAGCACTGCAATT
TTGTACCAGAATCTTGCAGAGCTATTTGTCAGCTACCTAACTACTAACTTGCAGCCATGGTTCATTGGGA
GGATGCCGAGAAACAATACATTGTCTCTGTCTTTTCAAAGATAGACGTTGACCATGTTGGTGCAAATACA
TTGGAAAGGGTCTTGATTGCTTTTCCTTGGACAAAGAGATACTTCAGTAGTTTTGGTGACCTTTCTAGTC
CTGGTGCCATTAAACATAATAACAAGGTGATCGCTCATGGAAGGAAAGTATTGGCTGCTATAATTGAATG
CACAAGGCATTTTGGAAATATCAAAGGTCATCTTGCCAATCTCAGCCACCTGCACTCTGAGAAACTCCAT
GTGGATCCACACAACTTCAGAGTTCTGGGACAATGTTTGAGGATCGAGCTGGCTGCCGCTCTTGGTTTTA
AGGAGTTCACTCCTGAAAGGAATGCTTACTTCCAAAAGTTTATGGATGTGATTTCTCACAGCCTGGGCCG
TGAGTACCACTAAACCATCCAAATGTGGCACAGGGGTTCATGATGCACCATCAAGAAAGCAGCTAATATT
CAATGTCAGTAGAGTCATAATGCAGAAACTGGCCCCCAGTACAGAACTTCAGGAAAATCTGCCAACAAAT
TGTATGATGTCATAATATAAAATGAGCTCCAGTGCAGATATGCATAGCTGTTCAGATACAATGTGCCTGT
TGACATTTATATAATAAATTGCTAAATCTTAAATGTTCAAGAAATGTCTGATATGCCTACACTTGGATTC
AGTTAAAATAAAGTAAAACCTT
>LpaHbB3
GCAAAGCCATCCATCAGCCACTTGCCATTAACTTGCAGCCATGGTTCACTGGGAATCCAATGAGAGACAA
ACCCTCAGCTCTGTGTGGGCAAAGATTGACAGTGAAGTTATAGGCGGAGAAGCTCTGGCAAGGCTCTTCA
TTGTCTATCCCTGGTCTCAGAGATACTTCAGTAAATTTGGTGACCTGTCTACTCATGATGCCATTAGCCA
CAACCCCAAGGTGAAAGCTCATGGCAAGAAAGTGCTAGCTGCTGTAGGTGATTGCTTAAAGCACCTGGAC
AACATCAAGGGTCATCTTACCGAGCACAGCCATCACCATTATAACGCACTCCATGTGGATCCAGCAAACT
TCACCCTTCTAGCATACTGTTTGGATATTGTTCTGGCCAGACATTTTGGTTATAAGGTGTTCACTCCTGA
AGTCCAAGGTACCTTCCAGAAATTTATGCGTGAGGTTACTGGTGGTCTTTCCACTGAGTACAGCTAAATC
ATACAAGTACAGCAAAGGGATTGTTGATGCACCATCAAGAGAGCAGCAAGTATCCAGAGTCAATGGATTC
GTAACGCAGAAACCAACAGCCAGTACACAACGTCAGGAAAATCCAAATACCAACAGATTGTATGATGTCA
TACTATACAATGAACTTCAGTTTAAATATGCATAGCTGTTCAGGTATGATGTGCCTGTTGACATTAATGT
CTCCACTCTTTAAATGTTAAATAAATGCCTGATAAGCTTATATTAAATTCAGTTAAAAATAAAATAAAAC
CTCTGTAAACACA
>LpaMb2
GGAAAAATAAAACTTCACTGTGCCTGTAGAAGATTCATCTGTGACTGCAGATATGACACTCTCAGACGCA
CAATGGAACAACGTCCTGGGGTTCTGGACAAAGCATATTGAGGGTGATCTTGCAAATATTGGCCATGAGG
TTTTAATTCGCCTGTTCTTACAAAGTAAAGCAGCCCAAAACCTCTTTGAGAAATTCAAGCACTTGAGCTC
AGAAGCAGAGATGAGGGGCTGTGCAGACCTACAGAAACATGGCAACACTGTCTTTACTGCCTTGGGAAAA
ACCCTGAAACTGAAGGGCCACCATGATGCTGAGCTTCGTCCAATGGCTGAGTCACATTCTAAGAAACACA
AAATCCCAGTGGAGAACTTCACGCTCATCTGCAATGTGATTGACAAGTATTTTCATGAGAAGTACCCAGA
TTATGCAGGAGAACTCAGAGAATCTTTTAAGCAAGCTACCCTTGGTGTTGCCCACACCTTGGAAAAACTG
TACAAAGAAGTGTAAAGATGGAAAAATGCTCTGGTGCTTACCAGTCAAGAAAAGAAACAACATCATTCTT
ACTTTATTCTTCATGTAACAATACCTGAGGTTAACTTTACTTCCTTAACAGTGACTGAGTATAAAATAAA
CGTTTTATGTGATCTATGAATAGTACTAAACTCTTTTTGGAAATAACTGAGTGCCTCAATATTTTAATGT
ACCCTGCATTTTTGATGGGTGTTTTGTTAGAACCAATAATACAAGGTGGTTGACTGATCATGCCACCCAG
CACAGGTCAAGTATAACTGAAAGAGCGACTTCTCTGTTTGTTGGCTTAAATCAAAAGCTGCTACGTAGCT
GATGTGTTGTTAGCAAGGACATGTGCTGTAATAAATGTATGGATAAATTATACAGTGAACAATGTCACAA
TAACAAAGGCTATGTTTTTTGTGATGCTGAACTCAAGTCGATAGAGGCAACTATGAGATCCTTCTAGTAG
TCCCAAAGTGTGAATCTGTAAAGTACCTTTGTGTGTACACACACACACACACA
>LpaMb3a
ACACACACACACACACACAGAATAGGTGCTGCAACCTCCACCCCAGCCCAGCCCCTCTCAGTGATGGCAA
AACTCTGATTGGCAGGTAGAAAGCTGTGAGAAAGAAAGGGGAGGATGGAAACCAGATAAAAGCCTTCAAT
TTTTCACAACACTTCAATTGCAGGGAAGAAGTTTTATTGTACCTGTACATAATAGTCTCTGTGATTGCGA
CTATGGCATCAGATGCACAGTGGAATGTTGCCTTAGACTTTTGGGAAAAAAATATTGCAAGTGATCCGAA
GAAACATGGTCATGCAGTTCTAATCGGCTTGTTCAAGAAAAGTAAAGACAGTCAAGTGCACTTTCCCAAA
TTTAAAGACCTTGCTAGTGAAGCAGAGATGAAAAGCTCTGAAGGGCTACAAAAACATGGCGAAACTGTGT
TCACTGCCTTAGGAAAAGCCCTAGCACAGAAGGATGGCATTGCTAATGAACTACGTCCTCTAGCAGAAAC
TCATGTCAAAGAGCACAAAATCCCTGTGGAAGAATTTTCAAAAATCTGTGATGTGATAAGCGATTATTGT
GCCATCGAATTTAAAGACTACACTGGAGATACCTGTACAGCTTTTAAGGCAGTGCTATCCGTTGTTGTCC
AAACCATGAACAATCTGTATAAAGAAGTGTAAATGTGACCTTCTCTAGTCTCTAATCTTTAGAAAGCACT
CTATTTCTCAGATTGTATGCATTCCATGTGACAATTTTCATATTTTCCCCCACACAGCAACTGTTTTTGC
TTTGTCAGATGTAATATAAACTAATAAAATCTTTTTTTGGAAAAAAAAAAAAAAA
>LpaMb3b
GATAATTTGCAGAAGTCTTTGAACTGAATGAGAAGCTATATATTTTTCACTGACTCCTTTTCAATACAGA
TGTGATACCATGAATCTTGTATTACTAATGACATATTTAGATTCCAAAGTAATTTAATTCCAGCAGAGGG
TATGCAGATAGACAAAAGCTTGCCCAAACAAGTCTCCACAATTATCCACCCCTGCCCATTTCAAGAAAGA
AAAAATGATTGGCAAATGAAGTCTGACAGAAAGAAAGAGAAAAAAAAAGAGAAGGCTGCAGAGCAGATAA
AAGGCATCCACCTCTTCCTAAAGTAAGAATTTAACTTTACCTGATTGAGAAAACTCTTCTTCCGATTCAG

AAATGGTAAAAGCTTCAAACTCACAGTACGAATGTCTCTATGCCTTTTTGATGCAAAGCATTGGGGAAGA
TCCTGCAGCGTTCGGTCATGAGAGTTTAATAAGTTTTTTCAAAGAAGATGAAGAAAACAAACAATATTTT
CCCAAGTTCAAACATCTTGCTAATGAAGCAGAGATGAGGAACTGTGCAGAGCTAAAGCAACATGGGAAAA
CAGTTGCCACGACTTTTGCAGACCTAGTTAAAAAGAAGGGAGACCATGATAAAGATATCAACAAACTGGC
CTTCTCGCATGTCCATGAGCACGGCGTTCCAGTAGGTAAATTTAAGGAAATTTTTGTCCATATGAAGAAA
AAACTAAGTCCAAAGCCAGGTTATACACCAGAGATTGATGCCAGCTTTGATGCAGTTACAGACAGCATTT
ACAACTGCTTGGAAGAGAATTACAAGAAAGTGAAAAAATAAATTGAACATCTACCACACCTAAAAAAAAG
GAATCCACTTACCATTAAACCGTTTAGTCTTAATGTGATGGAATTTGTACTTTCTTGTTTAACTCCATTA
CAATGCTTTTGATAGCATTCTGCTTTCTGTGCTCAAATGTAAAGCAAAGCAAACTTATTTTCCAACTAGC
TGAATAAGTTATACTTTACAGACATTTTTTTTTGGGGGGGGTTGAATTTTCATCACAAGCAGAACAGTGT
AAGTAGTCATGTTACAGATGAACTTTCAGAGAATCTTTTACTTGTCATTTGAATGTGTCCAGCTTTTTTT
ATGTTACAGTCTGTGTTGATGTCATCTGTATACATTGCATTGTAGACCCTTTACAAGAACAGAATCAACT
ATTTTACTGTGTTGGTTAAAGTGCAAAAGAGATAAATGTATAAAAGTTTTAAGAAGTCCATAATTAACTA
TATAGCAGGCTTGTGTGTACATGTGTGCATGCAAACACACACATGCACACACAA
>LpaMb5
AAGAAACTTCATTGTGTTGTAAGAAGATTCTTCTGCTGCTTTAGCTATGGCTGGACTTTCAGATGCACAA
TGGAATGACCTCTTGGGCTTCTTTGATAAATTCATTGCACCCAATTCGGCAGAACACGGCAAACACATTT
TAATTCGCATGTTTGAGCGTGACAGAGCAACCCAGAGTGTGTTTCCAAAGTTCAAGGATGTCTCTCCTTC
AGACTTGCCAAAGAATGCTGATGTGAAGAAACATGGTGGTGTTGTTGTTGATTTCTTGGGAAAACTGCTG
AAACAGAAGGGGCATAATGAAAGCATGTTGCACACTATGGCTGAGACTCATAAGAATACACACAAGGTTT
CACCGGATTATTTTCAACTAATTTCTTCTGTCATGGATGTATACATACATGAGAACTTGCCAGGAGAGTA
TGCACCTGTCCGGGATGCTATGAATGCAGCTCTGAAGCAGATTGCCAGTACCCTGAAAAGCAATTACTCC
AAAGTATAAGGACAAACTATTGTTATTATTTGTACCTCTTCCATAACAGAAAGACTTGATCATAAAACCA
TATATCTTTGTAATAGAACTATTATCTACAATTTACTGAAACTTTTCTAACAAAAATTAAAAAGAAAAAA
TGCATTTTGCTTTATAACTGTACTGTATTTGGGGAAGTCATGGAATGTGACTTTATAACAGCACTGATCC
ACTTATTTGAATAAAACCTCTTAACTGATATAGAAAACGTATCACACTAAGTGCCTATATTGTATAACAG
GGAATCAGAATACTAGATGTTAGAACTACAGTCTGTATTCCTTGCGTGTCTGAAACTTAAAAGACCCATC
TCATTTAAAATTAAGCAGAATCACATACTATATATATATATATAAATAT
>LpaMb7
ATGACAAGCCTTTCAGAAGCACAGTGGAATGAACTCTTGGCCTTTTGGGACAAATATGTTGCCCCAAGTT
CTTTAGAGCATGGCAAACACATTTTAATTCGCATGTTTCAGACAGACAAAGCAACTCAGGCCCTCTTTTC
AAAGTTCAAGGATATCCCTACTTCAGACCTAGCAATCAACGATGACGTGAAGAAACATGGTGGTGTTGTT
GTTGATTTCTTGGGAAAGCTGCTAAAACTGAAGGGTCAGAATGACAGTATGCTACACACTATGGCTCAGT
CTCATAAGAACAAGCACAAAATTCCACTCGACTACTTTCAATTAATTTCTTCTGTCATTGACGTGTATGT
GCATGAAAACCTGCCAGGAGAGTATGCACCTGTCCGTGAATCCATGAAAGCAGCACTGAGCCAGATTGCC
AATGGCCTGAAAGACAATTACACCAAAGTATAA

b. Sequences of *Protopterus annectens*

>PanGbE1a
ATGGCTTTGGCTGCTGATGATATTCAGAAGGCCAAGAGTGTTTGGGAGAAATTTTATGTAAATGCTGAGG
ATAATGGAGCAATTGTATTGAGCAGAATGTTCAAAGAGCACCCACATACTGTGAGCTACTTCACAAACTT
CAAAGAACTACAGTCCATTGCAGGAACTGCCTCAGCTGCAAAACTTGAAGGCCTATCAGAAGTTCGGACC
CATGGCAAGAAAGTTTTGTCTGCTCTGAATGATATGGTACAGCAAGTGGACAACATGGATGCTTTAAAAG
CTATTATTGAGCCTTTAGGCAAGAAGCATGCTGTGGAACTAAAAGTTGATGTGAAGGAATTTGAAATACT
TTGTGGAATTCTGCTGGACCTCATGGCTGAAAAATGTGGTGAAGATACCAAAACAGACTTCAAAAAAGTG
ACAGATGTTGTTTGTGAGCAAATCAAGTCTACTTATTGA
>PanGbE1b
ATGGCTTTGGCTGCTGATGATATTCAGAAGGCCAAGAGTGTTTGGGAGAAATTTTATGTAAATGCTGAGG
ACAATGGAGCAATTGTATTGAGCAGAATGTTCAAAGAGCACCCACATACTGTGAGCTACTTCACAAACTT
CAAAGAACTACAGTCCATTGCAGGAACTGCCTCAGCTGCAAAACTTGAAGGCCTATCAGAAGTTCGGGCC
CATGGCAAGAAAGTTTTGTCTGCCTTGAATGATATGGTATCACAAGTGGACAACATGGATGCTTTAAAAG
CTATTATTGAGCCTTTAGGCAAGAAGCATGCTGTGGAACTAAAAGTTGATGTAAAGGAATTTGAAATACT
TTGTGGAATTCTGCTGGACCTCATGGCTGAAAAATGTGGTGAAGATACCAAAACAGACTTCAAAAAAGTG
ACAGATGTTGTTTGTGAGCAAATCAAGTCTACTTATTGA
>PanGbE2a
ATGGCTCTTTCAGCTGAGGATACACAGACTGCTGGTGCTGTTTGGGAAAAGATTTATGCAGATGTTGAGG
ATAATGGTGCTGTTGTGTTAAGTAGAATGTTCAAAGAACACCATCATACTGTGAGCTACTTTAAAAATTT
TACACAGCTGCAGTCTGTTGCTGAGACTGCTTCTGCTGAGGAGATTGCAGCCCTGGCTGAAGTTCGAGCC
CATGGGAAGAAAGTTTTCTTAGCCTTGAATGATATGGTGCCACATTTAAACAATGTGGATGCTTTAAAAG
AAACTATTGCTCCATTAGCCAAGAAGCATGCTACAGAGCTTAAAATAGATGTGAAGGACTTCGAGATAAT

ATTCGATAATCTTTTGGCACTTATTGGAGAAAAGCAAGGTGCAGATGCCAAAACAGCCTTTAAGAAAGTG
ACAGATCTAATATATGAAGAAATAAAAGCTGCATATTGA
>PanGbE2b
ATGGCTCTTTCAGCTGAGGATACACAGACTGCTAGTGCTGTTTGGGAAAAAATCTATGCAGATGTTGAGG
ATAATGGTGCTGTTGTGTTAAGCAGAATGTTCAAAGAAAATCCTCATACTGTGAGCTACTTTAAAAACTT
CACACAGCTGCAGTCTATTGCTGAGACTGCTTCTGCTGAGGAGATTGCAGCCCTGGCTGAGGTTCGAGCC
CATGGAAAGAAGGTTTTCTCAGCCTTGAATGATATGGTATCACACTTGACCAATGTGGATGCTTTAAAAG
AGACCATTAATCCTCTAGCCAAGAAGCATGCTGCAGAACTTAAAGTAGATGTGAAGGACTTTAGGATAAT
ATTTGAAAATCTGCTGGATCTTATTGGAGAGAAGCAAGGTGCAGATGCCAAAACAGCCTTTAAGAAAGTG
ACAGATCTAATATATGAAGAAATAAAAGCTGCATATTGA
>PanGbE2c
ATGGCTCTTTCAGCTGAGGATACACAGACTGCTGGTGCTGTTTGGGAAAAGATTTATGCAGATGTTGAGG
ATAATGGTGCTGTTGTGTTAAGTAGAATGTTCAAAGAACACCATCATACTGTGAGCTACTTTAAAAATTT
TACACAGCTGCAGTCTGTTGCTGAGACTGCTTCTGCTGAGGAGATTGCAGCCCTGGCTGAAGTTCGAGCC
CATGGGAAGAAAGTTTTCTTAGCCTTGAATGATATGGTGCCACATTTAACCAATGTGGATGCTTTAAAAG
AAACTATTGCTCCATTAGCCAAGAAGCATGCTGCAGAACTTAAAGTAGATGTGAAGGACTTTAGGATAAT
ATTTGAAAATCTGCTGGATCTTATTGGAGAGAAGCAAGGTGCAGATGCCAAAACAGCCTTTAAGAAAGTG
ACAGATCTAATATATGAAGAAATAAAAGCTGCATATTGA

c. Sequences of *Protopterus aethiopicus*

>PaeGbE1a
ATGGCTTTGGCTGCTGATGATATTCAGAAGGTCAAAAGTGTTTGGGAGAAATTTTATGTAAATGCCGAGG
ACAATGGAGCAATTGTATTGAGCAGAATGTTCAAAGAGCACCCACATACTGTGAGCTACTTCACAAACTT
CAAAGAACTACAGTCCATTGCAGGAACTGCTTCAGCTGCAAAACTTGAAGGCCTATCTGAAGTTCGTGCC
CATGGCAAGAAAGTTTTGTCTGCTCTGAATGATATGGTACAGCAAGTGGATAACATGGATGCTTTAAAAG
CTATTATTGAGCCTTTAGGCAAGAAGCATGCTGTGGAACTAAAAGTTGATGTGAAGGAATTTGAAATACT
TTGTGGGATTCTGCTGGACCTCATGGCTGAAAAATGTGGTGAAGATACCAAAACAGACTTCAAAAAAGTG
ACAGATGTTGTTTGTGAGCAAATCAAGTCTACTTATTGA
>PaeGbE1b
ATGGCTTTGGCTGCTGATGATATTCAGAAAGCTAGGGGTGTTTGGGAGAAATTTTATGTAAATGCTGAGG
ATAATGGAGCAATTGTATTAAGCAGAATGTTCAAAGAGCACCCACATACTCTGAGCTACTTCACAAACTT
CAAAGAACTACAGTCCACTGCAGGAACTGCATCAGTTACAGAACTTGAAGGCCTATCAGAAGTTCGCACC
CATGGCAAGAAAGTTTTGTCTGCTCTGAATGATATGGTACAGCAAGTGGACAACATGGATGCTTTAAAAG
CTATTATTGAGCCTTTAGGCAAGAAGCATGCTGTGGAACTGAAAGTGGATGTAAAGGAATTTGAAATACT
TTGTGCAATTCTGCTGGAACTCATGGCTGAAAAATGTGGCGAAGATGCCAAGACAGACTTCAAAAAAGTG
ACAGATGTTGTGTGTGAACAAATCAAGTCTACTTATTGA
>PaeGbE1c
ATGGCTTTGGTTGCTGATGATATTCAGAAGGCCAAGAGTGTTTGGGAGAAATTTTATGTAAATGCTGAGG
ACAATGGAGCAATTGTATTGAGCAGAATGTTCAAAGAGCACCCACATACTGTGAGCTACTTCACAAACTT
CAAAGAACTACAGTCCATTGCAGGAACTGCCTCAGCTGCAAAACTTGAAGGCCTATCAGAAGTTCGTGCC
CATGGCAAGAAAGTTTTGTCTGCTCTGAATGATATGGTACAGCAAGTAGACAACATGGATGCTTTAAAAG
CTATTATTGAGCCTTTAGGCAAGAAGCATGCTGTGGAACTAAAAGTTGATGTGAAGGAATTTGAAATACT
CTGTGGAATTCTGCTGGACCTCATGGCTGAAAAATATGGTGAAGATACCAAAACAGACTTCAAAAAAGTG
ACAGATGTTGTTTGTGAGCAAATCAAGTCTACTTATTGA
>PaeGbE2a
ATGGCTCTTTCAGCTGAGGATACACAGACTGCTGGTGCTGTTTGGGAAAAGATTTATGCAGATGTTGAGG
ATAATGGTGCTGTTGTGTTAAGTAGAATGTTCAAAGAACACCATCATACTGTGAGCTACTTTAAAAATTT
TACACAGCTGCAGTCTGTTGCTGAGACTGCTTCTGCTGAGGAGATTGCAGCCCTGGCTGAAGTTCGAGCC
CATGGGAAGAAAGTTTTCTTAGCCTTGAATGATATGGTGCCACATTTAAGCAATGTGGATGCTTTAAAAG
AAACTATTGCTCCATTAGCCAAGAAGCATGCTACAGAGCTTAAAGTAGATGTGAAGGACTTCGAGATAAT
ATTCGATAATCTTTTGGCACTTATTGGAGAAAAGCAAGGTGCAGATGCCAAAACAGCCTTTAAGAAAGTG
ACAGATCTAATATATGAAGAAATAAAAGCTGCATATTGA
>PaeGbE2b
ATGGCTCTTTCAGCTGAGGATATACAAACTGTTAGTGCTATTTGGGAAAAAATCTATGCAGATGTTGAAG
ATAATGGTGCTGATGTGTTAAGTAGAATGTTCAAAGAACACCATCATACTGTGAATTATTTTAAAAATTT
CACTCAGCTGCAGTCTGTTGCTGAGACTGCCTCTGCTGTGGACATTGCAGCCCTGGCTGAAGTTCGAGCC
CATGGAAAGAAAGTTTTCTCAGCCTTGCATGATATGGTCCCACACTTGACCAATGTGGATGCTTTAAAAG
AGACCATTAATCCTCTAGCCAAGAAGCATGCTGCAGAACTTAAAGTAGATGTGAAGGACTTTAGGATACT
ATTTGAAAATCTGCTGGATCTTATTGGAGAGAAGCAAGGTGCAGATGCCAAAACAGCCTTTAAGAAAGTC
ACAGATCTAATATATGAAGAAATAAAAGCTGCATATTGA

```
>PaeGbE2c
ATGGCTCTTTCAGCTGAGGATGCCAAGACTGCTAGTGCTGTTTGGGAAAAAATCTATACAGATGTTGAGG
ATAATGGTGCTGTTGTATTAAGCAGAATGTTCAAAGAACACCATCATACTGTGAGCTACTTTAAAAACTT
CACACAGCTGCAGTCTGTTGCTGAGACTGCTTCTGCTCAGGAGATTGCAGCCCTGGCTGAAGTTCGAACC
CATGGGAAGAAAGTTTTCTCCGCCTTGAATGATATGGTATCACACTTAACCAATGTGGATGCTTTAAAAG
GGACCATTGCTCCATTAGCCAAGAAGCATGCTACAGAGCTTAAAGTAGATGTGAAGGACTTTGTGATAAT
CTTTGAAAATCTGCTGGATCTCATTGGAGAGAAGCAAGGTGGCGATGCCAAAGAAGCCTTTAAGAAAGTG
ACAGATGTAATGTATGAAGAAATAAAAGCTGCATATTGA
>PaeGbE2d
ATGGCTTTTTCAGCTGAGGATGCCAAGACTGCTAGTGCTGTGTGGGAAAAAATCTATGCAGATGTTGAGG
ATAATGGTGCTATTGTATTAAGCAGAATGTTCAAAGAAAATCCTCATACTGTGAGCTACTTTAAAAACTT
CACACAGCTGCAGTCTATTCCCGAGACTGCTTCTGCTGAGGAAATTGCAGCCCTGGCTGAGGTTCGAGGC
CATGGAAAGAAGGTTTTCTCAGCCTTGAATGATCTGGTATCACACTTGACCAATGTGGATTCTTTAAAAG
CGACCATTGTTCCATTAGCCAAGAAGCATGCTACTGAGCTAAAAGTAGATGTGAAGGACTTTGGGATCAT
TTTTGAAAATCTGCTGCATCTCATTGGACAGAAGCAAGGTGGAGATGCCAAAGAAGCTTTCGAGAAAGTT
ACAAATCTAATATATGAAGAAATAAAAGCTGCATATTGA
```

Supplemental Information Fig. 1. Nucleotide sequences obtained in this study.

a. Sequences from *Lepidosiren paradoxa*

```
>LpaGbE1
MAMSADDIQKGRSAWEKFYANAEDNGAVVLSRMFKEHPHTVSYFKNFKELQSMAEKASSVELQGLSEVRG
HGKKVLSALNDMVQYLDNMDSLKKVTEPLGKKHAVELQVDVKDFDIIFTILLDLLGEKCGGDAKTDLKKV
TDLLYEEIKSTY
>LpaGbE2a
MALSAEDIQMVSGVWSKIFADAESNGAVVLSRMFKEYPHTVKYFKNFPELQSIAETASAADIAGLAEVRG
HAKTVLTAFNDMVQHLENIDTLKETATPLAKKHSEELKVDVKDFKILCDNLVDLVGEKQDEDAKTTFKKA
VDVIYENISAAY
>LpaGbE2b
MALSAEEIQNATGVWSKIFADAESNGSVVLSRMFKEYPHTVKYFKNFPELQSVGETASAAEIAGLAEVQG
HAKTVFTAFNDMVQHLENIDALKETATPLAKKHSEELKVDVKDFKILCDNLVDLVGEKQDEDAKSTFKKA
VDEIYENIKAAY
>LpaGbE2c
MAFSAEDIQTATDVWDKIFVNAEENGAIVLSRMFKEHSHTVSHFKNFTELQSIAETGSAAEIAALAEVQG
HGKKVFTALNDLVLKVDNADALKEAVAPLAKKHAEELKVDVKDLGVLCEILIDLVGEKQGDAKTAFKKVM
DVIYENIKAAY
>LpaGbE2d
MALSAEDIQTATGVWDKIFVNAEENGAIVLSRMFKEHSHTVSHFKNFTELQSIAETASAAEIAALAEVQG
HGKKVFTALNDLVLHVDNADALNKIAAPLAKKHAEELKVDVKDLGVLCEILIDLVGEKQGEDAKTAFKKV
MDVIYENLKAAY
>LpaGbE2e
MALSAEDIQTAIGVWDKIVVNAEEHGAIVLSRMFKEHSHTVSHFKNFTELQSIAEIASAAEIAGLAEVRG
HGKKVFTALNDLVLHVDNADALNKIAAPLAKKHAEELKVCVKDLGVLCEILIDLVGEKQGDAKTAFKKVM
DVIYENIKAAY
>LpaGbX
MGCALSEVGKEENSASIVNEISGEIGPDTDIGSGTQSGSMSNLSTLSESQKELIQESWKILHQDITRVGI
IVFIRLFETHPECKDVFFLFRDIDDVQQLKISKELQAHGLRVMSFIEKSVARLQQEDKLQHIAFELGRCH
CRYNALPKYFEYVGFQFMTAVKPILKEKWTSEVEDAWKSLFKYLISMMKKGFHEEAKNRLSNKSTDPKKQ
LTTRMKFVQHNV
>LpaGbY
MSCLSMTDIQNIRNIWGIICQNPEANGQIVVTRLFVDYPQTTLYFKNFKNLDTVEGIKESKQVREHGRRV
VMMLDKVIENLGDWNETSNLLSKLAKRHRDYHNIPVRNFKFLFASLNSVYIDVFGPAFTADIQTSWEKLY
SLVYQEMEKCYSAIPSS
>LpaHbA1
MRFSQDDEVLIKEAWGLLHQIPNAGGEALARMFSCYPGTKSYFPHFGHDFSANNEKVKHHGKKVVDAIGQ
GVQHLHDLSSCLHTLSEKHARELMVDPCNFQYLIEAIMTTIAAHCGEKFTPEINCAAEKCLGQIVHVLIS
LYR
>LpaHbA2
MLLTQAEKSLLSSSYDKIAPNAEQYGSELFNRMFASFPQTRTYFPNVDTSPKSAQLRAHGAKIIAALGKG
IKNIDNVSAALSDLSDLHAKNIKVDPANFPYISHCFLVLLAAHMQGDFTPELHLAWDKYLQLTGRVLTSK
YR
>LpaHbB1
MVHWTNEEEYAIFSIWGDIDVHEEGHQTLTRLMVVYPWTKRYFSSFGDLSTSCSIAANHLVKAHGAKVLT
AIGDALVDMPNIKHNLTDLSRLHSEILHVDPENFRLLGKCLLIVLAAKFGAKKFNVDVHAAWKKLMEIIA
AGLSKQYH
>LpaHbB2
MVHWEDAEKQYIVSVFSKIDVDHVGANTLERVLIAFPWTKRYFSSFGDLS
>SPGAIKHNNKVIAHGRKVLAAIIECTRHFGNIKGHLANLSHLHSEKLHVD
>PHNFRVLGQCLRIELAAALGFKEFTPERNAYFQKFMDVISHSLGREYH"
>LpaHbB3
MVHWESNERQTLSSVWAKIDSEVIGGEALARLFIVYPWSQRYFSKFGDLSTHDAISHNPKVKAHGKKVLA
AVGDCLKHLDNIKGHLTEHSHHHYNALHVDPANFTLLAYCLDIVLARHFGYKVFTPEVQGTFQKFMREVT
GGLSTEYS
>LpaMb2
MTLSDAQWNNVLGFWTKHIEGDLANIGHEVLIRLFLQSKAAQNLFEKFKHLSSEAEMRGCADLQKHGNTV
FTALGKTLKLKGHHDAELRPMAESHSKKHKIPVENFTLICNVIDKYFHEKYPDYAGELRESFKQATLGVA
HTLEKLYKEV
>LpaMb3a
```

MASDAQWNVALDFWEKNIASDPKKHGHAVLIGLFKKSKDSQVHFPKFKDLASEAEMKSSEGLQKHGETVF
TALGKALAQKDGIANELRPLAETHVKEHKIPVEEFSKICDVISDYCAIEFKDYTGDTCTAFKAVLSVVVQ
TMNNLYKEV
>LpaMb3b
MVKASNSQYECLYAFLMQSIGEDPAAFGHESLISFFKEDEENKQYFPKFKHLANEAEMRNCAELKQHGKT
VATTFADLVKKKGDHDKDINKLAFSHVHEHGVPVGKFKEIFVHMKKKLSPKPGYTPEIDASFDAVTDSIY
NCLEENYKKVKK
>LpaMb5
MAGLSDAQWNDLLGFFDKFIAPNSAEHGKHILIRMFERDRATQSVFPKFKDVSPSDLPKNADVKKHGGVV
VDFLGKLLKQKGHNESMLHTMAETHKNTHKVSPDYFQLISSVMDVYIHENLPGEYAPVRDAMNAALKQIA
STLKSNYSKV
>LpaMb7
MTSLSEAQWNELLAFWDKYVAPSSLEHGKHILIRMFQTDKATQALFSKFKDIPTSDLAINDDVKKHGGVV
VDFLGKLLKLKGQNDSMLHTMAQSHKNKHKIPLDYFQLISSVIDVYVHENLPGEYAPVRESMKAALSQIA
NGLKDNYTKV

b. Sequences of *Protopterus annectens*

>PanGbE1a
MALAADDIQKAKSVWEKFYVNAEDNGAIVLSRMFKEHPHTVSYFTNFKELQSIAGTASAAKLEGLSEVRT
HGKKVLSALNDMVQQVDNMDALKAIIEPLGKKHAVELKVDVKEFEILCGILLDLMAEKCGEDTKTDFKKV
TDVVCEQIKSTY
>PanGbE1b
MALAADDIQKAKSVWEKFYVNAEDNGAIVLSRMFKEHPHTVSYFTNFKELQSIAGTASAAKLEGLSEVRA
HGKKVLSALNDMVSQVDNMDALKAIIEPLGKKHAVELKVDVKEFEILCGILLDLMAEKCGEDTKTDFKKV
TDVVCEQIKSTY
>PanGbE2a
MALSAEDTQTAGAVWEKIYADVEDNGAVVLSRMFKEHHHTVSYFKNFTQLQSVAETASAEEIAALAEVRA
HGKKVFLALNDMVPHLNNVDALKETIAPLAKKHATELKIDVKDFEIIFDNLLALIGEKQGADAKTAFKKV
TDLIYEEIKAAY
>PanGbE2b
MALSAEDTQTASAVWEKIYADVEDNGAVVLSRMFKENPHTVSYFKNFTQLQSIAETASAEEIAALAEVRA
HGKKVFSALNDMVSHLTNVDALKETINPLAKKHAAELKVDVKDFRIIFENLLDLIGEKQGADAKTAFKKV
TDLIYEEIKAAY
>PanGbE2c
MALSAEDTQTAGAVWEKIYADVEDNGAVVLSRMFKEHHHTVSYFKNFTQLQSVAETASAEEIAALAEVRA
HGKKVFLALNDMVPHLTNVDALKETIAPLAKKHAAELKVDVKDFRIIFENLLDLIGEKQGADAKTAFKKV
TDLIYEEIKAAY

c. Sequences of *Protopterus aethiopicus*

>PaeGbE1a
MALAADDIQKVKSVWEKFYVNAEDNGAIVLSRMFKEHPHTVSYFTNFKELQSIAGTASAAKLEGLSEVRA
HGKKVLSALNDMVQQVDNMDALKAIIEPLGKKHAVELKVDVKEFEILCGILLDLMAEKCGEDTKTDFKKV
TDVVCEQIKSTY
>PaeGbE1b
MALAADDIQKARGVWEKFYVNAEDNGAIVLSRMFKEHPHTLSYFTNFKELQSTAGTASVTELEGLSEVRT
HGKKVLSALNDMVQQVDNMDALKAIIEPLGKKHAVELKVDVKEFEILCAILLELMAEKCGEDAKTDFKKV
TDVVCEQIKSTY
>PaeGbE1c
MALVADDIQKAKSVWEKFYVNAEDNGAIVLSRMFKEHPHTVSYFTNFKELQSIAGTASAAKLEGLSEVRA
HGKKVLSALNDMVQQVDNMDALKAIIEPLGKKHAVELKVDVKEFEILCGILLDLMAEKYGEDTKTDFKKV
TDVVCEQIKSTY
>PaeGbE2a
MALSAEDTQTAGAVWEKIYADVEDNGAVVLSRMFKEHHHTVSYFKNFTQLQSVAETASAEEIAALAEVRA
HGKKVFLALNDMVPHLSNVDALKETIAPLAKKHATELKVDVKDFEIIFDNLLALIGEKQGADAKTAFKKV
TDLIYEEIKAAY
>PaeGbE2b
MALSAEDIQTVSAIWEKIYADVEDNGADVLSRMFKEHHHTVNYFKNFTQLQSVAETASAVDIAALAEVRA
HGKKVFSALHDMVPHLTNVDALKETINPLAKKHAAELKVDVKDFRILFENLLDLIGEKQGADAKTAFKKV
TDLIYEEIKAAY

```
>PaeGbE2c
MALSAEDAKTASAVWEKIYTDVEDNGAVVLSRMFKEHHHTVSYFKNFTQLQSVAETASAQEIAALAEVRT
HGKKVFSALNDMVSHLTNVDALKGTIAPLAKKHATELKVDVKDFVIIFENLLDLIGEKQGGDAKEAFKKV
TDVMYEEIKAAY
>PaeGbE2d
MAFSAEDAKTASAVWEKIYADVEDNGAIVLSRMFKENPHTVSYFKNFTQLQSIPETASAEEIAALAEVRG
HGKKVFSALNDLVSHLTNVDSLKATIVPLAKKHATELKVDVKDFGIIFENLLHLIGQKQGGDAKEAFEKV
TNLIYEEIKAAY
```

Supplemental Information Fig. 2. Amino acid sequences obtained in this study. The GbE peptide sequences identified by mass spectrometry in the ovaries of *L. paradoxa* and *P. aethiopicus* are underlined.

```
#NEXUS
Begin data;
    Dimensions ntax=192 nchar=256;
    Format datatype=protein gap=- missing=X matchchar=. interleave;
    Matrix
AcaGbY       ------------------------------------------------------MTDLDRRHIREIWTA-AFENPEENGRLVIIRFFSDYPASKQYF---
AcaMb        ME----------------------------------------------------LSDQEWQKVIDIWGK-VEPEIPAYGQVVILRLFEQHPETQEKFDKF
AplGbE       MP----------------------------------------------------FSEAEVQSARGAWEK-IYVDAEDNGTAVLVRMFTEHPDTKSYFTHF
CanMb        MA--------------------------------------------------------DFDMVLKCWGP-VEADHATHGSLVLTRLFTEHPETLKLFPKF
CauMb1       MA--------------------------------------------------------DHELVLKCWGV-VEADFEGTGGEVLTRLFKQHPETQKLFPKF
CauMb2       MA--------------------------------------------------------DYERFLKCWGA-VEADYTGNGGEVLTRLFKAHPDTQKLFPKF
CcaMb1       MA--------------------------------------------------------DHELVLKCWGG-VEADFEGTGGEVLTRLFKQHPETQKLFPKF
CcaMb2       MA--------------------------------------------------------DYERFLKCWGA-IEADYAGHGGEVLTRLFKEHPDTLKLFPKF
CcrMb        MG----------------------------------------------------LSDGEWQLVLNIWGK-VEADIPGHGQEVLIRLFKNHPETLEKFDKF
CliGbE       MS----------------------------------------------------FSEAEVQGARGAWEK-MYADAEDNGTTVLVRMFTEHPDTKSYFTHF
CmiGbY       MT---------------------------------------------------GITEADKENIHFIWEK-LYENPEENGKTIVLRMFTDYPETKMYFQHF
CmyGbE       MA----------------------------------------------------FSEAEVQRARGAWEK-MYANAEDNGTTVLVRMFTEHPDTKSYFTHF
CpiCygb      ME------------------------------------KVQGEMEIERWERSEELSDAEKKVIQETWSR-VYMNCEDVGVSILIRFFVKFPSAKQYFSQF
CpiGbE       MA----------------------------------------------------FSEAEVQRARGAWEK-MYANAEDNGTTVLVRMFTEHPDTKSYFTHF
CpiGbX       MGCALSGS------GIAPGKTISESKRSPSSENLAAPKAGPEHGGDGLGAGPFPLADAQKERIQESWRI-LHDNIARVGIIVFIRLFETHPECKDVFFLF
CpiGbY       MA---------------------------------------------------LLTDADKKNIQHIWAK-LFENPEENGKTIVIKLFKDYPETKAYF---
CpiHbA       MV----------------------------------------------------LNAGDKANVKAVWNK-VAAHVEEYGAETLERMFTVYPQTKTYFPHF
CpiHbaA      MV----------------------------------------------------LNAGDKANVKAVWNK-VAAHVEEYGAETLERMFTVYPQTKTYFPHF
CpiHbaD      -M----------------------------------------------------LNHDEKQLIKHAWEK-VLGHQEDFGAEALERMFAVYPQTKTYFPHF
CpiHbB1      MV---------------------------------------------------HWTAEEKQLITNLWGK-V--NVAECGSEALARLLIVYPWTQRFFSSF
CpiHbB2      MV---------------------------------------------------HWTADEKQLITSLWGK-V--NVEECGSEALARLLIVYPWTQRFFSTF
CpiHbG       MV---------------------------------------------------HWTAEEKQLITSLWGK-V--NAEECGGEALARLLIVYPWTQRFFSSF
CpiHbZ       MT----------------------------------------------------LTQAEKAAVVAIWGK-IAAQADALGTESLERLFSSFPQTKTYFPHF
CpiMb        MG----------------------------------------------------LSDDEWHHVLGIWAK-VEPDLSAHGQEVIIRLFQVHPETQERFAKF
CpiNgb       ME----------SGR---------------------------------------LSSTQQALIRESWQK-VSSNLLQHGIVLFTRLFDLDPDLLPLFQYN
CraMb        MA--------------------------------------------------------DFDMVLKCWGP-MEADHATHGSLVLTRLFTEHPETLKLFPKF
CycrMb       MG----------------------------------------------------LSDGEWHLVLNVWGK-VETDLAGHGQEVLIRLFKSHPETLEKFDKF
DreCygb1     ME---------------------------------------GDGGVQLTQSPDSLTEEDVCVIQDTWKP-VYAERDNAGVAVLVRFFTNFPSAKQYFEHF
DreCygb2     ME------------------------------------KEREDEETEGRERPEPLTDVERGIIKDTWAR-VYASCEDVGVTILIRFFVNFPSAKQYFSQF
DreGbX       MGCAISGS------GL---------------------TAGAPEIRPGEEETPAGLTTNHIRLIKESWRL-IQEDIAKVGIIMFVRLFETHPECKDVFFLF
DreHbAa      MS----------------------------------------------------LSDTDKAVVKAIWAK-ISPKADEIGAEALARMLTVYPQTKTYFSHW
DreHbAe      MS----------------------------------------------------LSAKDKAAVKTLWAK-IAGKADDIGHDALSRMLIVYPQTKTYFSHW
DreHbAx      MP-----------------------------------------------------SSAEKELIAEIWDQ-MTPVAEEIGSEALLRMFTTFPQTKTYFSHL
DreHbBa      MV---------------------------------------------------EWTDAERTAILGLWGK-L--NIDEIGPQALSRCLIVYPWTQRYFATF
DreHbBe      MV---------------------------------------------------VWTDFEKATIQDIFAK-A--DYDVIGPQALARCLIVYPWTQRYFAKF
DreMb        MA--------------------------------------------------------DHDLVLKCWGA-VEADYAANGGEVLNRLFKEYPDTLKLFPKF
DreNgb       ME---------------------------------------------------KLSEKDKGLIRDSWES-LGKNKVPHGIVLFTRLFELDPALLTLFSYS
EpeMb        MA--------------------------------------------------------DLDAVLKCWGA-VEADFNTVGGLVLARLFKDHPETQKLFPKF
FalGbE       MS----------------------------------------------------LSEAEVQSARGAWEK-IYVDAEDNGTAVLVRMFTEHPDTKSYFTHF
FpeGbE       MS----------------------------------------------------FSEAEVQSARGAWEK-IYVDAEDNGTTVLVRMFTEHPDTKSYFTHF
FruMb        MA--------------------------------------------------------DFETVLKFWGP-VEADYGAHGGIVLTRLFTENPETQKLFPKF
GfoGbE       MS----------------------------------------------------LSEAEVQSARGAWEK-IYVDAEDNGTAVLVRMFTEHPDTKSYFTHF
GgaCygb      ME------------------------------------KVQGEMEIERWERSEEISDAEKKVIQETWSR-VYANCEDVGVSILIRFFVNFPSAKQYFSQF
GgaGbE       MS----------------------------------------------------FSEAEVQSARGAWEK-MYVDAEDNGTAVLVRMFTEHPDTKSYFTHF
GgaHbA       MV----------------------------------------------------LSAADKNNVKGIFTK-IAGHAEEYGAETLERMFTTYPPTKTYFPHF
GgaHbAD      MM----------------------------------------------------LTAEDKKLIQQAWEK-AASHQEEFGAEALTRMFTTYPQTKTYFPHF
GgaHbG       MV---------------------------------------------------HWTAEEKQLITGLWGK-V--NVAECGAEALARLLIVYPWTQRFFASF
GgaMb        MG----------------------------------------------------LSDQEWQQVLTIWGK-VEADIAGHGHEVLMRLFHDHPETLDRFDKF
GgaNgb       ME----------SGM---------------------------------------LSRTQQALIRESWRR-VSGSPVQHGVVLFSRLFDLDPDLLPLFQYN
GgiMb        MA--------------------------------------------------------DFDMVLKCWGP-VEADYTTHGSLVLTRLFTEHPETLKLFPKF
HsaCYGB      ME------------------------------------KVPGEMEIERRERSEELSEAERKAVQAMWAR-LYANCEDVGVAILVRFFVNFPSAKQYFSQF
HsaHBA       MV----------------------------------------------------LSPADKTNVKAAWGK-VGAHAGEYGAEALERMFLSFPTTKTYFPHF
HsaHBB       MV---------------------------------------------------HLTPEEKSAVTALWGK-V--NVDEVGGEALGRLLVVYPWTQRFFESF
HsaHBD       MV---------------------------------------------------HLTPEEKTAVNALWGK-V--NVDAVGGEALGRLLVVYPWTQRFFESF
HsaHBE       MV---------------------------------------------------HFTAEEKAAVTSLWSK-M--NVEEAGGEALGRLLVVYPWTQRFFDSF
HsaHBG       MG---------------------------------------------------HFTEEDKATITSLWGK-V--NVEDAGGETLGRLLVVYPWTQRFFDSF
HsaHBZ       -S----------------------------------------------------LTKTERTIIVSMWAK-ISTQADTIGTETLERLFLSHPQTKTYFPHF
HsaMB        MG----------------------------------------------------LSDGEWQLVLNVWGK-VEADIPGHGQEVLIRLFKGHPETLEKFDKF
HsaNGB       ------------------------------------------------------MERPEPELIRQSWRA-VSRSPLEHGTVLFARLFALEPDLLPLFQYN
IpuGbX       MGCAISGL------GL----------------------APKNTTEAASEDDAPHLTSEHIAMIKESWKV-IQEDIAKVGIIVFVRLFETHPECKDVFFLF
LchCygb      ME------------------------------------KVQGEMEMDRWERSDQLSDTEVESIRQIWSN-VYTNCENVGVLVLIRFFVNFPSAKQYFSQF
LchGbE       MA----------------------------------------------------LSDAEVQTARDVWGQ-IYANAEENGTIILVRMFTEHPDTKSYFGNF
LchGbX1      MGCVFSGS------GIAPSKSTPDINGS----EAESRLSEELKSGSGQNSDALLLSEPQKELIQESWRI-LHQDITRLGIIMFIRLFETHPECKDVFFLF
LchGbX2      MGCAISGLSWRAVKGP----------------------GEEAKGKEGEAEALPAISQLQIHLIQESWKL-IQEDIAKVGIIMFVRLFETHPECKDVFFLF
LchGbY       MA---------------------------------------------------ALTEADKQNIRGIWKT-VFENAEENGRTIVIRLFEKYPETKVYFKNF
LchHbA1      M-----------------------------------------------------LSANDKTLISSTWNK-VAANAEDIGAEALERLFLAHPQTKIYFSHM
LchHbA2      MG----------------------------------------------------LTAADKTLIKSIWGK-VEKETEAIGVEALVRLFKCFPQSKVYFDHF
LchHbB1      MV---------------------------------------------------HWTETERATIETVYQK-L--HLDEVGREALTRLFIVYPWTTRYFKSF
LchHbB2      MV---------------------------------------------------TWTAEERKAITSVWSK-V--NPEEVGHEALIRLFIVYPWTQRYFSTF
LchMb        MA----------------------------------------------------LSEAEWGLILKVWGK-AEPEAASNGKSVLLRMFQEHPDTQQHFPKF
LchNgb       ME----------KHN---------------------------------------FSVRSKELIRESWDR-LGKNKLPHGTVMFTRLFELDPDLMHLFNYN
LpaGbE1      MA----------------------------------------------------MSADDIQKGRSAWEK-FYANAEDNGAVVLSRMFKEHPHTVSYFKNF
LpaGbE2a     MA----------------------------------------------------LSAEDIQMVSGVWSK-IFADAESNGAVVLSRMFKEYPHTVKYFKNF
LpaGbE2b     MA----------------------------------------------------LSAEEIQNATGVWSK-IFADAESNGSVVLSRMFKEYPHTVKYFKNF
LpaGbE2c     MA----------------------------------------------------FSAEDIQTATDVWDK-IFVNAEENGAIVLSRMFKEHSHTVSHFKNF
LpaGbE2d     MA----------------------------------------------------LSAEDIQTATGVWDK-IFVNAEENGAIVLSRMFKEHSHTVSHFKNF
LpaGbE2e     MA----------------------------------------------------LSAEDIQTAIGVWDK-IVVNAEEHGAIVLSRMFKEHSHTVSHFKNF
LplaHbA      MP-------------------------------------------IVDSGSVGAISAAEKSLIVSAWAP-VYAKYEEAGVDILVKFFAANPEAQAFFPKF
LplaHbB      MP-------------------------------------------IVDSGSVPALTAAEKATIRTAWAP-VYAKYQSTGVDILIKFFTSNPAAQEFFPKF
MangMb       MG----------------------------------------------------LSDGEWHLVLNIWGK-VETDLAGHGQEVLIRLFRSHPETLEKFDKF
MbiMb        MG----------------------------------------------------LSEAEWQLVLHVWAK-VEADLSGHGQEILIRLFKGHPETLEKFDKF
MgaGbE       MS----------------------------------------------------FSEAEVQSARGAWEK-IYVDAEDNGTAVLIRMFTEHPDTKSYFTHF
MglaHb2      MS----------------------------------------------AHGIARTTEGERAAVRASWAV-LMKDYEHAGVQILDKFFKANPAAKPFFTKM
MmuMb        MG----------------------------------------------------LSDGEWQLVLNVWGK-VEADLAGHGQEVLIGLFKTHPETLDKFDKF
MniMb        MA--------------------------------------------------------DFEMVLKHWGP-VEADYATHGNLVLTRLFTEHPETQKLFPKF
MunGbE       MS----------------------------------------------------FSEAEVQSARGAWEK-IYVDAEDNGTAVLVRMFTEHPDTKSYFSHF
NcoMb        MA--------------------------------------------------------DFDMVLKCWGP-MEADYATHGGLVLTRLFTEHPETLKLFPKF
OanGbY       MV---------------------------------------------------QVTDVEKANIQSIWSK-MMENLEKNGIDIFTRLFREYPETKKYF---
OanHbW       MV---------------------------------------------------NWTSEEKHAIVSIWGK-V--DIEETGANALSRLLVVYPWTQRYFSAF
OanMb        MG----------------------------------------------------LSDGEWQLVLKVWGK-VEGDLPGHGQEVLIRLFKTHPETLEKFDKF
OanNgb       ME----------NGR---------------------------------------LSGPEQELIRESWRS-VNSNPLEHGMILFTRLFDLEPDLLPLFQYN
OlaCygb1     ME------------------------------------RKQ--GEVDHLERSRPLTDKERVMIQDSWAK-VYQNCDDAGVAILVRLFVNFPSSKQYFSQF
OlaCygb2     MSCRESPPPPSPPPQML---------------------GVQRGECEDRPERAEPLSDAEMEIIQHTWGH-VYKNCEDVGVSVLIRFFVNFPSAKQYFSQF
```

```
OlaHbA      MS----------------------------------------------------LSAKDKAAVKAFWAK-VSGQADAIGSDALSRMLVVYPQTKTYFAHW
OlaHbB      MV---------------------------------------------------EWTEQERSIITNIFGN-L--DYEDVGSKALSRCLIVYPWTQRYFASF
OlaMb       MA--------------------------------------------------------DYDMVLKHWGP-VEADYNTHGNLVLTRLFHEYPETQKLFPKF
OlaNgb      ME---------------------------------------------------KLSGKDKELIRGSWES-LGKNKVPHGVIMFSRLFELDPALLSLFNYN
PaeGbE1a    MA----------------------------------------------------LAADDIQKVKSVWEK-FYVNAEDNGAIVLSRMFKEHPHTVSYFTNF
PaeGbE1b    MA----------------------------------------------------LAADDIQKARGVWEK-FYVNAEDNGAIVLSRMFKEHPHTLSYFTNF
PaeGbE1c    MA----------------------------------------------------LVADDIQKAKSVWEK-FYVNAEDNGAIVLSRMFKEHPHTVSYFTNF
PaeGbE2a    MA----------------------------------------------------LSAEDTQTAGAVWEK-IYADVEDNGAVVLSRMFKEHHHTVSYFKNF
PaeGbE2b    MA----------------------------------------------------LSAEDIQTVSAIWEK-IYADVEDNGADVLSRMFKEHHHTVNYFKNF
PaeGbE2c    MA----------------------------------------------------LSAEDAKTASAVWEK-IYTDVEDNGAVVLSRMFKEHHHTVSYFKNF
PaeGbE2d    MA----------------------------------------------------FSAEDAKTASAVWEK-IYADVEDNGAIVLSRMFKENPHTVSYFKNF
PanGbE1a    MA----------------------------------------------------LAADDIQKAKSVWEK-FYVNAEDNGAIVLSRMFKEHPHTVSYFTNF
PanGbE1b    MA----------------------------------------------------LAADDIQKAKSVWEK-FYVNAEDNGAIVLSRMFKEHPHTVSYFTNF
PanGbE2a    MA----------------------------------------------------LSAEDTQTAGAVWEK-IYADVEDNGAVVLSRMFKEHHHTVSYFKNF
PanGbE2b    MA----------------------------------------------------LSAEDTQTASAVWEK-IYADVEDNGAVVLSRMFKENPHTVSYFKNF
PanGbE2c    MA----------------------------------------------------LSAEDTQTAGAVWEK-IYADVEDNGAVVLSRMFKEHHHTVSYFKNF
PanGbX      MGCALSR---------AGATDNSTTDVNEILEEMGPDTVIGSRTQSEVVSDLSTLAESQKELIQESWKI-LHQDIARLGVIVFIRLFETHPECKDVFFMF
PanGbY      MC---------------------------------------------------SLSTTDIKNIRDIWSI-VCQNPEENGRTVVIRLFLDYPQTRTYFKNF
PanHba1     MP----------------------------------------------------LSKAEKTILLAVWER-ISPYIEEFGAQALTRMFRCFPETKIYFHEK
PanHba2     MT----------------------------------------------------LTAAEKSCLASTFEK-IAPKAEQYGSELFQRMFLGFPQTRTYFAHV
PanHba3     MT----------------------------------------------------FTREDEVHIGEAC-K-LLTQIPNAGGEALARMFAAFPGTKSYFQKF
PanHba4     MT----------------------------------------------------FTHEDEVHIAEAC-K-LLTQIPNAGGEALARMFAAFPGTKSYFQKF
PanHbb1     MV---------------------------------------------------HWTSEEEFAITSIWGN-V--DVHEEGHDTLTRLMVVYPWTKKYFSSF
PanHbb2     MV---------------------------------------------------LWDAAEKKVIASVWAK-V--DIEADGFQALLRTLHVYPWTKRYFSHF
PanHbb3     MV---------------------------------------------------NWKANERQAVTSVWAS-I--DAAGHGQETLERLLHVYPWSRRYFGKF
PanHbb4     MV---------------------------------------------------NLEAAEKQTITSFWAS-V--DPAAQGENALQRLLYCYPWSRRYFAKF
PanHbb5     MV---------------------------------------------------HWDATEKQTITSFWGK-V--DLAADGEHSLQRLLYVYPWCRRYFHKF
PanMb1      MA---------------------------------------------------SLSDAQWKKLQEFWVKNVEPNLTKHGQEVLVRMFVNHKSTLEYFPKF
PanMb2      MT----------------------------------------------------LSEAQWNNVLAFWAKHIENDPTKHGHEVLIRLFLESKAAQNLFDKF
PanMb3      MA-----------------------------------------------------SAAQWDTTLKFWEAHVAGDLKKHGHEALVRLFLKNKDSQKHFPKF
PanMb4      MA---------------------------------------------------GLSEVQWNELLAFWDKYVAPSSSEHGKHILIRMFQTEKATQTLFSKF
PanMb5      MA---------------------------------------------------GLSDAQWNDLLAFFDKFIAPNSAEHGKHILIRMFDSDRATQSLFPKF
PanMb6a     MACP-----------------------------------------------------------AKFWEENVVPDAAEHGKNILIRLYKEDPAALGFFPKY
PanMb6b     MACP-----------------------------------------------------------AKFWEENVVPDAAEHGKNILIRLYKEDPAAQGFFSKY
PanMb7      MA---------------------------------------------------GLSEAQWNELLAFWDKYVAPSSSEHGKHILIRMFDADKATQALFSKY
PcaMb       MV----------------------------------------------------LSEGEWQLVLHVWAK-VEADVAGHGQDILIRLFKSHPETLEKFDRF
PgeMb       MA--------------------------------------------------------DFDMVLKCWGL-VEADYATYGSLVLTRLFTEHPETLKLFPKF
PhuGbE      MS----------------------------------------------------LSEAEVQSARGAWEK-IYVDAEDNGTAVLVRMFTEHPDTKSYFTHF
PmaaHb1     MP-------------------------------------------IVDSGSVPALTAAEKATIRTAWAP-VYAKYQSTGVDILIKFFTSNPAAQAFFPKF
PmaaHb10    MP-------------------------------------------IVDSGSVGALSASEKAAVAGSWKA-VYANYEAAGKAVLIKFFTSNPGVQDFFPKF
PmaaHb11    MP-------------------------------------------IVDSGSAGALSAAEKAIITDSWKV-VYADYEAAGKAILIKFFTSNPGVQDFFPKF
PmaaHb12    MP-------------------------------------------IVDSGSVGEFSAAEKSLIVSAWAP-VYAKYEEAGVDILVKFFSDNPGVQDFFPKF
PmaaHb13    MP-------------------------------------------IVDSGSVGAISAAEKSLIVSAWAP-VYAKYEEAGVDILVKFFAANPEAQAFFPKF
PmaaHb14    MP-------------------------------------------IVDSGSVGAISAAEKSLIVSAWAP-VYAKYEEAGVDILVKFFAANPEAQAFFPKF
PmaaHb2a    MP-------------------------------------------IVDTGSVAPLSAAEKTKIRSAWAP-VYSNYETSGVDILVKFFTSTPAAQEFFPKF
PmaaHb3     MP-------------------------------------------IVDSGSVAPLSAAEKTKIRSAWAP-VYSNYETTGVDILVKFFTSTPAAQEFFPKF
PmaaHb5a    MP-------------------------------------------IVDTGSVAPLSAAEKTKIRSAWAP-VYSTYETSGVDILVKFFTSTPAAQEFFPKF
PmaaHb5b    MP-------------------------------------------IVDSGSVAPLSAAEKTKIRSAWAP-VYSTYETSGVDILVKFFTSNPAAQEFFPKF
PmaaHb6     MG------------------------------------------ALQDSGIVSSFKEDEKAALRESWDI-FNNSHQDAGVKILARFIINNPEAKKFFPKF
PmaaHb7     MP-------------------------------------------IEDTGSKPDFSDEEKKAIKDSWSG-VYSEYESASSEILIKFFVDNPSAQDFFPKF
PmaaHb8     MP-------------------------------------------IVDSGSVAPLSAAEKTKIRSAWAP-VYSNYETSGVDILVKFFTSTPAAQEFFPKF
PmaaHb9     MP-------------------------------------------IVDSGSVGALSGAEKAAIADSWKA-VYSNYEEAGKAILIKFFTSNPGVQDFFPKF
PmaaMb1     MS-------------------------------------------IADSGSAPALSGDEKSAVRDTWKV-VYPHAEDHGTTILIKFLTENADAKKFFPKF
PmaaMb2     MS------------------------------------------AIVDSGSAPALSGDEKAAIKSTWPS-VFAKAEDVGAEMLSRFISSNADVKKYFPKF
PmaCygb     ME-------------------------------------------------QGWLSEEEIEALQDIWEK-VFKSAEDVGVILLVRLFTGHPASKQYFPMF
PmaGbX1     MGCTVSTD------ERTGAQSSSQGQSQASRKQQQPEQQRAAGEGHQPPGPPQAPSESQRRLVRDSWLA-LQCDIARVGVIMFVRLFETHPECKDVFYQF
PsiCygb     ME------------------------------------KVQGEMEIERWERSEELSDAEKKVIQETWSR-VYTNCEDVGVSILIRFFVQFPSAKQYFSQF
PsiGbE      MA----------------------------------------------------FSEAEVQRARGAWEK-MYANAEDNGTTVLVRMFTEHPDTKSYFTHF
PsiGbX      MGCALSGS------GSAPGKRSSEAEQ----KDRAAWKAGPVRGGEGLEAGPFPPAGAQKERIQESWRI-LHDSIARVGIIVFIRLFETHPECKDAFFLF
PsiGbY      MA---------------------------------------------------LLTDADKKSIHHIWSK-LFENPEENGKIIVIRLFKDYPETKAYF---
PsiHbaA     MV----------------------------------------------------LTACDKTNVKAVWTK-VSGHLEDYGAETLERMFATYPSTKTYFAHF
PsiHbaD     -M----------------------------------------------------LTADEKQLVLHAWDK-VQGHQEDFGAEALERMFTTYPSTKTYFPHF
PsiHbB      MV---------------------------------------------------HWTAEEKQFITSLWGK-V--DVAECGGEALARLLIVYPWTQRFFSSF
PsiHbG      MV---------------------------------------------------HWTPEEKQMITSLWAK-V--NVAECGGEALARMMIVYPWTQRFFSTF
PsiHbZ      MT----------------------------------------------------LTQAEKAAVVAIWEK-VATHANAIGAESLERLFSGFPQTKTYFPHF
PsiMb       MG----------------------------------------------------LSDNEWQHVLGIWAK-VESDIPAHGQEVMIRLFQVHPETQSLFAKF
PsiNgb      ME-----------SGR--------------------------------------LSSTEKALIRESWQK-VSSNLLQHGIVLFTRLFDLDPDLLPLFQYN
RnoCygb     ME------------------------------------KVPGDMEIERRERNEELSEAERKAVQATWAR-LYANCEDVGVAILVRFFVNFPSAKQYFSQF
RnoMb       MG----------------------------------------------------LSDGEWQMVLNIWGK-VEGDLAGHGQEVLISLFKAHPETLEKFDKF
SchMb       MA--------------------------------------------------------DFDAVLKFWGP-VEADYTSHGGLVLTRLFKEHPETQKLFPKF
SjaMb       MA--------------------------------------------------------DFDAVLKFWGP-VEADYDKIGNMVLTRLFTEHPDTQKLFPKF
TalaMb      MA--------------------------------------------------------DFDAVLKCWGP-VEADYTTIGGLVLTRLFKEHPDTQKLFPKF
TalbMb      MA--------------------------------------------------------DFDAVLKCWGP-VEADYTTMGGLVLTRLFKEHPETQKLFPKF
TguCygb     ME------------------------------------KVQGEMEIERWERSEEISDAEKKVIQEIWSR-VYANCEDVGVSILIRFFVNFPSAKQYFSQF
TguGbE      MS----------------------------------------------------LSEAEVQSARGAWEK-IYVDAEDNGTAVLVRMFTEHPDTKSYFPHF
TguHbA      MV----------------------------------------------------LSAGDKSNVKAVFGK-IGGQADEYGADALERMFATYPQTKTYFPHF
TguHbAD     -M----------------------------------------------------LTGEDKKLLQQTWGK-LGGAEEEVGADALWRMFHSYPPTKTYFPHF
TguHbE      MV---------------------------------------------------NWTAEEKQLVTTLWGR-V--NVDECGAEALARLLVAYPWTQRFFVSF
TguMb       MG----------------------------------------------------LSDQEWQQVLTVWGK-VESDLAGHGHQILMRLFQDHPETLDRFEKF
TguNgb      ME-----------SGM--------------------------------------RLSGGQRALIRESWQR-VSGSPVQHGLVLFTRLFDLDPDLLPLFQYN
TniCygb1    ME------------------------------------RMQRDGEVDHVEQPGPLTEKEKVMIQDSWAK-VFQSCDDAGVAILVRFFVNFPSSKQFFKDF
TniGbX      MGCAISSL------GA---------------KAEFGDRSAEEEDAAAAAAAVVYPREDQIQMIKDSWKV-IRDDIAKVGIIMFVRLFETHPECKDVFFLF
TniMb       MG--------------------------------------------------------DFDMVLKFWGP-VEADYSAHGGMVLTRLFTENPETQQLFPKF
TniNgb      ME---------------------------------------------------KLSSKDKELIRGSWDS-LGKNKVPHGVILFSRLFELDPELLNLFHYT
TruCygb1    ME------------------------------------RMQGDGELDHVERPSPLTDKEKVMIQDSWAV-VFQSCDDAGVAILVRLFVSFPSSKQLFKDF
TruCygb2    MSHRESPPAPPPPPQLLGG-------------------QRRDVEGEDGPERAKPLSDTEREMIQDTWGH-IYKNCEDVGVSVLIRFFVNFPSAKRYFSQF
TruNgb      ME---------------------------------------------------KLSSKDKELIRGSWDS-LGKNKVPHGVIMFSRLFELDPELLSLFHYT
TthMb       MA--------------------------------------------------------DFDAVLKCWGP-VEADYTTIGGLVLTRLFKEHPETQKLFPKF
XlaGbY      MA---------------------------------------------------DLTAADIENINEIWCK-IYANPEESGKTVVIRLFTTYPQTKVYFKNL
XlaHbA1     -T----------------------------------------------------LTDSDKAAVIALWGK-IAPQANAIGAEALERLFLSYPQTKTYFSHF
XlaHbAT5    MT----------------------------------------------------FSSAEKAAIASLWGK-VSGHTDEIGAEALERLFLSYPQTKTYFSHF
XtrCygb     ME------------------------------------KVQGENDMERWERLEEITESERGVIKETWAR-VYANCEDVGVSILIRFFVNFPSAKQHFSQF
XtrGbX      MGCILSSL------GWQWRDSLDHTETSP-------------------LLPTLNLSEQQQQLLVESWRL-IQHDIAKVGVILFVRLFETHPECKDVFFLF
XtrGbY      MA---------------------------------------------------DLTGADIENINEVWSK-IYANPEESGRTVVISLFLTYPQTKIYFKNL
XtrHbA1     MH----------------------------------------------------LTADDKKHIKAIWPS-VAAHGDKYGGEALHRMFMCAPKTKTYFPDF
XtrHbB1     MV---------------------------------------------------NLTAKERQLITGTWSK-I--CAKTLGKQALGSMLYTYPWTQRYFSSF
XtrHbB2     MV---------------------------------------------------HWTAEEKATIASVWGK-V--DIEQDGHDALSRLLVVYPWTQRYFSSF
XtrNgb      ME-----------KDQ--------------------------------------LSGPQKELIRESWQT-VSQDQLHHGTVLFSRLFELEPELVFLFQYN
ZalGbE      MS----------------------------------------------------LSEAEVQSARGAWEK-IYVDAEDNGTAVLVRMFTEHPDTKSYFTHF

AcaGbY      -KTVP------TDGDLKAHPQVAFHGRRIMVAFSQVIENMENWNQACVLLERLVNNHKNIHQ-VPSGMFQLLFQAMLCTFDDLLGRT-FTP-EKRVSWEK
AcaMb       -KNLK------SLDEMKNSEDLKKHGTIVLTALGKILKQKRQHE---AELAPLAQSHATKHK-IPVKYLEFISEVIVGVIAEKRSAD-FGA-ESQAAMRK
AplGbE      -KGMD------SAEEMKQSDQVRGHGKRVFTAINDMVQHLDNTEAFLGILNPLGQKHATQLK-VDPKNFRIICDIILQMMEEKFGG------DCKASFEK
```

```
CanMb        -AGI-------AHGDLAGDAGVSAHGATVLKKLGDLLKARGGHA---ALLKPLSSSHATKHK-IPIINFTLIAEVIGKVMEEKAGLD---A-AGQTALRN
CauMb1       -VGI-------AQSDLAGNAAVNAHGATVLKKLGELLKARGDHA---AILKPLATTHANKHK-IALNNFRLITEVLVKVMAEKAGLD---A-AGQTALRK
CauMb2       -KGI-------SQSELAGNALVAAHGATVLKKLGELLRAKGDHA---AILHPMATTHANKHK-ITLNNFRLITEVLVEVMKEKAGLD---S-AGQGALKR
CcaMb1       -VGI-------AQSDLAGNAAVKAHGATVLKSWASCLKARGDHA---AILKPLATTHANTHK-IALNNFRLITEVLVKVMAEKAGLD---A-GGQSALRR
CcaMb2       -KGI-------PQSELAGDTLVASHGATVLKKLGELLRAKGDHA---AILQPLATTHANKHK-IALNNFRLITEVLVKVMAEKAGLD---T-AGQGALKR
CcrMb        -KSLK------SEGEMKASEDLKKHGATVLTALGGILKKKGQHA---AELQPLAQSHANKHK-IPVKYLEFISEAIIQVLQSKHSGD-FGA-DTKEAMKK
CliGbE       -KGMD------SAEEMKQSDQVRGHGKKVFTAINDMVQHLDNSEAFLGIVNPLGKKHATQLK-IDPKNFRIICDIILQLMEEKFGG------DCKSSFEK
CmiGbY       -KNIS------TLEEMKKSPQIKRHGKIVMSALNKLIANLDNGEELSSLLAKMAERHINVHK-VDLHNFQIIFNIIIAILEETFGNA-FTP-EIRGTWTK
CmyGbE       -KGMG------TAEEMEQSDQVRSHGKRVLTTINDLVQHLDSTDAFLGIVNPLGKKHAMQLK-VDPKNFRIICDIILQLMEEKYGG------DCKASFEK
CpiCygb      -KHME------DPLEMERTPQLRKHARRVMGAVNTVVENINDSEKVSSVLALVGKAHALKHK-VEPVYFKFFTGVMLEVIAEEYAND-FTP-EVQRAWTK
CpiGbE       -KGMG------TAEEMEQSDQVRSHGKRVLTTINDLVQHLDSTDAFLGIVNPLGKKHAMQLK-VDPKNFRIICDIILQLMEEKYGG------DCKASFEK
CpiGbX       -RDIE------DLEQLKMNKELRAHGLRVMSFIEKSVARLDQEDKLEQLAFELGRSH-YRYN-APPKYYEYVGIQFISTVQPILKER-WTP-EVEEAWQV
CpiGbY       -KNIP------TEGNLQEDPLVRFHGRRVMVALNQVVENLDNWKQACRILDRLADKHKNVHQ-VPAVNFQSMFQVILNVCKDVLGNE-FST-EVSLSWEK
CpiHbA       --DLH------H-----GSAQIRTHGKKVLTALGEAVNHIDDLA---SALSKLSDIHAQTLR-VDPVNFKFLNHCFLVVVAIHQPSV-LTP-EVHVSLDK
CpiHbaA      --DLH------H-----GSAQIRTHGKKVLTALGEAVNHIDDLA---SALSKLSDIHAQTLR-VDPVNFKFLNHCFLVVVAIHQPSV-LTP-EVHVSLDK
CpiHbaD      --DLH------H-----DSEQIRHHGKKVVTALGDAVRHMDNLS---EALSELSNLHAYNLR-VDPVNFKLLSHCFQVVLAVHLADE-YTP-QVHVAYDK
CpiHbB1      -GNLS------SPTAIIGNPKVRAHGKKVLTSFGEAVKNLDNIK---ATYAKLSELHCDKLH-VDPENFRLLGDILVIVLAAHFGRE-FTP-ACQAAWQK
CpiHbB2      -GNLS------NAEAILHNPHVHAHGKKVLTSFGEAVKNLDHIK---QTFATLSKLHCEKLH-VDPENFKLLGNVLIIVLASHFTKE-FTP-ACQAAWQK
CpiHbG       -GNLS------SPTAITGNPKVRAHGKKVLTSFGEAVKNLDNIK---ATYAKLSELHCNKLH-VDPENFRLLGDILVIVLASHFGRE-FTP-ACQAAWQK
CpiHbZ       --DLS------Q-----GSAQLHGHGSKVLGAIGEAVKNIDNIT---GALATLSELHAYILR-VDPVNFKLLSHCILCSVAAHFPND-FTP-EVHAAWDK
CpiMb        -KNLK------TIDELKSSEEVKKHGTTVLTALGRILKLKNNHE---PELKPLAESHATKHK-IPVKYLEFICEIIVKVIAEKHPSD-FGA-DSQAAMRK
CpiNgb       CKKFS------SPQECLSSPEFLDHIRKVMLVIDAAVTHLENLSSLEEYLSNLGKKH-QTVG-VKVDSFSAVGESLLFMLEKCLGTA-FSP-DVREAWTR
CraMb        -AGI-------AHGDLAGDAGVSAHGATVLNKLGDLLKARGAHA---ALLKPLSSSHATKHK-IPIINFKLIAEVIGKVMEEKAGLD---A-AGQTALRN
CycrMb       -KHLK------SEDDMRRSEDLRKHGNTVLTALGGILKKKGHHE---AELKPLAQSHATKHK-IPIKYLEFISEAIIHVLHSKHPAE-FGA-DAQAAMKK
DreCygb1     -RELQ------DPAEMQQNAQLKKHGQRVLNALNTLVENLRDADKLNTIFNQMGKSHALRHK-VDPVYFKILAGVILEVLVEAFPQC-FSPAEVQSSWSK
DreCygb2     -QDME------DPEEMEKSSQLRKHARRVMNAINTVVENLHDPEKVSSVLVLVGKAHAFKYK-VEPIYFKILSGVILEILAEEFGEC-FTP-EVQTSWSK
DreGbX       -RDVE------DLERLRTSRELRAHGLRVMSFIEKSVARLDQLERLETLALELGKSH-YRYN-APPKYYGYVGAEFICAVRPILKDR-WTP-ELEEAWKT
DreHbAa      -ADLS------P-----GSGPVKKHGKTIMGAVGEAISKIDDLV---GGLAALSELHAFKLR-VDPANFKILSHNVIVVIAMLFPAD-FTP-EVHVSVDK
DreHbAe      -KDLS------P-----GSAPVRKHGKTVMGGVAEAVSKIDDLN---AGLLNLSELHAFQLR-VDPANFKILSHNILVVLATLFPAD-FTP-EAHVAMDK
DreHbAx      --NIS------A-----NSEHLRSHGKKIVEALAEGAKNVSTLT---TTLAPLSRFHAYQLR-IHPTNFKLFNHCILVTLACRMGDD-FTP-VVHAAIDK
DreHbBa      -GNLS------SPAAIMGNPKVAAHGRTVMGGLERAIKNMDNVK---NTYAALSVMHSEKLH-VDPDNFRLLADCITVCAAMKFGQAGFNA-DVQEAWQK
DreHbBe      -GNLY------NAAAILGNPMVAAHGKTVLKGLELAVKNMDNIK---ATYADLSVLHSEKLH-VDPDNFRLLADCLTIVVAAQMGAG-FTP-EVQAAFQK
DreMb        -SGI-------SQGDLAGSPAVAAHGATVLKKLGELLKAKGDHA---ALLKPLANTHANIHK-VALNNFRLITEVLVKVMAEKAGLD---A-AGQGALRR
DreNgb       -TNCG------DAPECLSSPEFLEHVTKVMLVIDAAVSHLDDLHTLEDFLLNLGRKH-QAVG-VNTQSFALVGESLLYMLQSSLGPA-YTT-SLRQAWLT
EpeMb        -AGI-------T-GDIAGNAAVAAHGATVLKKLGELLKAKGNHA---AIIKPLANSHAKQHK-IPINNFKLITEALAHVLHEKAGLD---A-AGQTALRN
FalGbE       -KGMD------SAEEMKQSDQVRGHGKKVFGAINDMVQHLDNSEAFLGILTPLGKKHASQLK-IDPKNFRIICDIILQLMEEKFGG------DCKASFEK
FpeGbE       -KGMD------SAEEMKQSDQVRGHGKKVFSAINDMVQHLDNSEAFLGIVNPLGKKHATQLK-IDPKNFRILCDIILQLMEEKFGG------DCKASFEK
FruMb        -AGI-------TQSDLAGNAAVSAHGATVLKKLGELLKAKGNHA---ALLQPLANTHATKHK-IPINNFKLIAEVIGKVMEEKAGLD---A-AGQQALKN
GfoGbE       -KGMD------SAEEMKQSDQVRGHGKKVFSAINDMVQHLDNSEAFLGIVTPLGKKHATQLK-IDPKNFRIICDIILQLMEEKFGG------DCKASFEK
GgaCygb      -KHMD------DTLEMERSLQLRKHAQRVMGAINTVVENLDDPEKVSSVLALVGKAHALKHK-VEPVYFKKLTGVMLEVIAEAYGND-FTP-EAHGAWTK
GgaGbE       -KGMD------SAEEMKQSDQVRGHGKRVFTAINDMVQHLDNTEAFLGILNPLGQKHATQLK-IDPKNFRIICDIILQLMEEKFGG------DCKASFEK
GgaHbA       --DLS------H-----GSAQIKGHGKKVVAALIEAANHIDDIA---GTLSKLSDLHAHKLR-VDPVNFKLLGQCFLVVVAIHHPAA-LTP-EVHASLDK
GgaHbAD      --DLS------P-----GSDQVRGHGKKVLGALGNAVKNVDNLS---QAMAELSNLHAYNLR-VDPVNFKLLSQCIQVVLAVHMGKD-YTP-EVHAAFDK
GgaHbG       -GNLS------SPTAILGNPMVRAHGKKVLSSFGDAVKNLDNIK---NTFSQLSELHCDKLH-VDPENFRLLGDILIIVLAAHFSKD-FTP-ECQAAWQK
GgaMb        -KGLK------TPDQMKGSEDLKKHGATVLTQLGKILKQKGNHE---SELKPLAQTHATKHK-IPVKYLEFISEVIIKVIAEKHAAD-FGA-DSQAAMKK
GgaNgb       CKRFA------SPQECLAAPEFLDHIRKVMLVIDAAVSHLEDLPCLEEYLCNLGKKH-QAVG-VKVESFSTVGESLLYMLEKCLGAA-FSP-DVREAWIE
GgiMb        -AGI-------AHGDLAGDAGVSAHGATVLNKLGDLLKARGAHA---ALLKPLSSSHATKHK-IPIINFKLIAEVIGKVMEEKAGLD---A-AGQTALRN
HsaCYGB      -KHME------DPLEMERSPQLRKHACRVMGALNTVVENLHDPDKVSSVLALVGKAHALKHK-VEPVYFKILSGVILEVVAEEFASD-FPP-ETQRAWAK
HsaHBA       --DLS------H-----GSAQVKGHGKKVADALTNAVAHVDDMP---NALSALSDLHAHKLR-VDPVNFKLLSHCLLVTLAAHLPAE-FTP-AVHASLDK
HsaHBB       -GDLS------TPDAVMGNPKVKAHGKKVLGAFSDGLAHLDNLK---GTFATLSELHCDKLH-VDPENFRLLGNVLVCVLAHHFGKE-FTP-PVQAAYQK
HsaHBD       -GDLS------SPDAVMGNPKVKAHGKKVLGAFSDGLAHLDNLK---GTFSQLSELHCDKLH-VDPENFRLLGNVLVCVLARNFGKE-FTP-QMQAAYQK
HsaHBE       -GNLS------SPSAILGNPKVKAHGKKVLTSFGDAIKNMDNLK---PAFAKLSELHCDKLH-VDPENFKLLGNVMVIILATHFGKE-FTP-EVQAAWQK
HsaHBG       -GNLS------SASAIMGNPKVKAHGKKVLTSLGDAIKHLDDLK---GTFAQLSELHCDKLH-VDPENFKLLGNVLVTVLAIHFGKE-FTP-EVQASWQK
HsaHBZ       --DLH------P-----GSAQLRAHGSKVVAAVGDAVKSIDDIG---GALSKLSELHAYILR-VDPVNFKLLSHCLLVTLAARFPAD-FTA-EAHAAWDK
HsaMB        -KHLK------SEDEMKASEDLKKHGATVLTALGGILKKKGHHE---AEIKPLAQSHATKHK-IPVKYLEFISECIIQVLQSKHPGD-FGA-DAQGAMNK
HsaNGB       CRQFS------SPEDCLSSPEFLDHIRKVMLVIDAAVTNVEDLSSLEEYLASLGRKH-RAVG-VKLSSFSTVGESLLYMLEKCLGPA-FTP-ATRAAWSQ
IpuGbX       -RDVE------DLERLRTSKELRAHGLRVMSFIEKSVARLDQLERLDSLAVELGRSH-YRYY-APPKYYGYVGTEFICAVRPILKEK-WTP-ELEKAWKT
LchCygb      -RHLE------DPLDMERSVQLRKHARRVMGAINTVVENVEDQD-IASVLAPVGKAHALKHK-VEPVYFKILSGVILEILAEEYAQH-FTP-EVQKAWTK
LchGbE       -KGMG------SAAEMEQSAQVRTHGKKIFSALNDMIQHLDSTDALLGVVNPLGKKHATQLK-VDPKNFKIICNILLQVLDEKFGG------DARAGFEK
LchGbX1      -RDID------DFHQLKMSKELQAHGLRVMSFIEKSVARLEQKDKLEQIAFELGRSH-CRYN-APPKYYQYVGVQFISVVKPILKEA-WTP-EVEEAWQI
LchGbX2      -RDVD------DLQRLRTSKELRAHGLRVMSFVEKSVARLEHSERLEQLALELGKSH-YRYN-APPKYYQYVGIEFVCAVQPILKER-WTP-EVEEAWQT
LchGbY       -KNIS------TMEEMQKNEQIRIHGLRVMNSLNQVIQNIDNLNEVYSILTHLAKRHQYVHR-VDVHNFKLIFGVIIKILKEALGAT-FTE-EICTSWQK
LchHbA1      --DLS------P-----GSSMLRAHGKKVMGTIEGSIKSIDKLA---TVLSRLSDMHAYNFM-VDPVNFKLLSQCILVALATQLMAD-FTP-EAQCAWDK
LchHbA2      -TDLS------P-----SSQKLHAHAKVVLGALTKAVNHLDNIT---DTLHDISLVHAKKLL-VDPVNFELLGHCLEVALAAHFATD-FTP-EVHLAIDK
LchHbB1      -GDLS------SSKAIASNPKVTEHGLKVMNKLTEAIHNLDHIK---DLFHKLSEKHFHELH-VDPQNFKLLSKCLIIVLATKLGKQ-LTP-DVQATWEK
LchHbB2      -GSLS------SSTVIARNFKVQQHAAKVINALTEAIRNIDNLK---ASFSDLSKLHFQKLH-VDPENFKLLGKTLIITLSEKLGSE-FSP-QIQAAWEK
LchMb        -KHM-------TYQELQSSEELKTHGDTVLSKLGCLLKLKGNHA---GDLHPLAQTHATKHK-IPLHNFEIISEIIVKILAEKYPGD-FGA-DGQAALKK
LchNgb       -SSFP------SQAGCLTSPEFIDHIHKVMMVVDAAVNSLDNLLSLEDYLINLGKKH-QAAG-VKMESFQVVGESLLYMLEHGLDSA-FSV-EVRHAWIA
LpaGbE1      -KELQSMAEKASSVELQGLSEVRGHGKKVLSALNDMVQYLDNMDSLKKVTEPLGKKHAVELQ-VDVKDFDIIFTILLDLLGEKCGG------DAKTDLKK
LpaGbE2a     -PELQSIAETASAADIAGLAEVRGHAKTVLTAFNDMVQHLENIDTLKETATPLAKKHSEELK-VDVKDFKILCDNLVDLVGEKQDE------DAKTTFKK
LpaGbE2b     -PELQSVGETASAAEIAGLAEVQGHAKTVFTAFNDMVQHLENIDALKETATPLAKKHSEELK-VDVKDFKILCDNLVDLVGEKQDE------DAKSTFKK
LpaGbE2c     -TELQSIAETGSAAEIAALAEVQGHGKKVFTALNDLVLKVDNADALKEAVAPLAKKHAEELK-VDVKDLGVLCEILIDLVGEKQG-------DAKTAFKK
LpaGbE2d     -TELQSIAETASAAEIAALAEVQGHGKKVFTALNDLVLHVDNADALNKIAAPLAKKHAEELK-VDVKDLGVLCEILIDLVGEKQGE------DAKTAFKK
LpaGbE2e     -TELQSIAEIASAAEIAGLAEVRGHGKKVFTALNDLVLHVDNADALNKIAAPLAKKHAEELK-VCVKDLGVLCEILIDLVGEKQG-------DAKTAFKK
LplaHbA      -KGLD------SADQLKKSPAVRWHAERIINAVNDAVVAMDDPAKQSLQLKELSQKHAHELN-VDPKYFKVLAGVISDAVVK-------SG-DAKAAVDK
LplaHbB      -QGLT------SADQLKKSMDVRWHAERIINAVNDAVVAMDDTEKMSLKLNELSSKHAKSFQ-VDPQYFKVLAAVIVDTVL-----------PGDAGLEK
MangMb       -KHLK------SEDDMRRSEDLRKHGNTVLTALGGILKKKGHHE---AELKPLAQSHATKHK-IPIKYLEFISEAIIQVLHNKHPGE-FGA-DTQAAMKK
MbiMb        -KHLK------SEAEMKASEDLKKHGHTVLTALGGILKKKGHHE---AELKPLAQSHATKHK-IPIKYLEFISDAIIHVLHSKHPSD-FGA-DAQGAMTK
MgaGbE       -KGMD------SAEEMKQSDQVRGHGKRVFTAINDMVQHLDNTEAFLGILNPLGQKHATQLK-IDPKNFRIICDIILQLMEEKFGG------DCKTSFEK
MglaHb2      -KDLH------TLEDLASSADARWHVERIIQAVNFAVINIEDREKLSNKFVKLSQDHIEEFHVTDPQYFMILSQTILDEVEKRNGG--LSG-EGKSGWHK
MmuMb        -KNLK------SEEDMKGSEDLKKHGCTVLTALGTILKKKGQHA---AEIQPLAQSHATKHK-IPVKYLEFISEIIIEVLKKRHSGD-FGA-DAQGAMSK
MniMb        -AGI-------AKADMAGNAAISAHGATVLKKLGELLKAKGSHA---AIIKPMANSHATKHK-IPIKNFELISEVIGKVMHEKAGLD---A-AGQKALKN
MunGbE       -KGMD------SAEEMKQSDQIRGHGKRVFTAINDMVQHLDNSEAFLGIVNPLGKKHATQLK-IDPKNFRVICDIILQLMEEKFGG------DCKASFEK
NcoMb        -AGI-------AHGDLAGDAGVSAHGATVLNKLGDLLKARGAHA---ALLKPLSSSHATKHK-IPIINFKLIAEVIGKVMEEKAGLD---A-AGQTALRN
OanGbY       -KNIP------LEGNLQEDPLLRSHGRRVMVALNRIIQNLDNWKQVCKILNPLAEKHKIIHS-VDVENFQFMLKCVGDVCQDYLGPC-YTP-EIAESFQK
OanHbW       -GNLS------SPTAIVGNPRVRAHGKKVLTSFGEAVKDLDHVK---SNFAKLSQLHSEKLH-VDPENFRLLGDNLIIVLAATLGKD-FTP-EAQAAWQK
OanMb        -KGLK------TEDEMKASADLKKHGGTVLTALGNILKKKGQHE---AELKPLAQSHATKHK-ISIKFLEYISEAIIHVLQSKHSAD-FGA-DAQAAMGK
OanNgb       CRQFS------SPRDCLASPEFLDHIRKVMLVIDAAVIHLDDLSSLEEYLTNLGRKH-KAIG-VKLSSFSTVGESLLFMLDKCLGPA-FSP-ATREAWTR
OlaCygb1     -KHIE------DAEELEKSSQLRKHARRVMNAINTLVESLDNSDKVSSVLNAVGKAHAIRHK-VDPVYFKILSGVILEVLGEAYPQV-MTA-EVASAWTN
OlaCygb2     -QDMQ------DPEEMEKSSQLRQHARRVMNAIN-VVENLQDPEKVSSVLALVGKAHAVKHK-VEPIYFKILSGVMLSVLSEDFPEF-FTA-EVQLVWTK
OlaHbA       -KDLS------P-----GSAPVKKHGKTVMGGIADAVGKIDDIS---SGLLNLSELHAFTLR-VDPTNFKILSHNILVVMAIMFPQD-FTP-EVHVALDK
OlaHbB       -GNLY------NAEAIKTNPNIAAHGTKVLHGLDRAVKNMDNIK---ATYAELSVLHSEKLH-VDPDNFKLLADCLTIVIAAKLGSA-FSP-EIQATFQK
OlaMb        -AGI-------AKGDMAGNAALSAHGATVLKKLGELLKARGNHG---AILKPLANSHATKHK-IPINNFRLITEVIGKVMAEKAGLD---A-AGQQALRS
OlaNgb       -TNCG------STQDCLSSPEFLDHVTKVMLVIDAAVNHLDDLHSLEDFLLNLGRKH-QAVG-VSTQSFAVVGESLLYMLQCSLGQA-YTA-ALSQAWLN
PaeGbE1a     -KELQSIAGTASAAKLEGLSEVRAHGKKVLSALNDMVQQVDNMDALKAIIEPLGKKHAVELK-VDVKEFEILCGILLDLMAEKCGE------DTKTDFKK
PaeGbE1b     -KELQSTAGTASVTELEGLSEVRTHGKKVLSALNDMVQQVDNMDALKAIIEPLGKKHAVELK-VDVKEFEILCAILLELMAEKCGE------DAKTDFKK
PaeGbE1c     -KELQSIAGTASAAKLEGLSEVRAHGKKVLSALNDMVQQVDNMDALKAIIEPLGKKHAVELK-VDVKEFEILCGILLDLMAEKYGE------DTKTDFKK
PaeGbE2a     -TQLQSVAETASAEEIAALAEVRAHGKKVFLALNDMVPHLSNVDALKETIAPLAKKHATELK-VDVKDFEIIFDNLLALIGEKQGA------DAKTAFKK
PaeGbE2b     -TQLQSVAETASAVDIAALAEVRAHGKKVFSALHDMVPHLTNVDALKETINPLAKKHAAELK-VDVKDFRILFENLLDLIGEKQGA------DAKTAFKK
PaeGbE2c     -TQLQSVAETASAQEIAALAEVRTHGKKVFSALNDMVSHLTNVDALKGTIAPLAKKHATELK-VDVKDFVIIFENLLDLIGEKQGG------DAKEAFKK
PaeGbE2d     -TQLQSIPETASAEEIAALAEVRGHGKKVFSALNDLVSHLTNVDSLKATIVPLAKKHATELK-VDVKDFGIIFENLLHLIGQKQGG------DAKEAFEK
PanGbE1a     -KELQSIAGTASAAKLEGLSEVRTHGKKVLSALNDMVQQVDNMDALKAIIEPLGKKHAVELK-VDVKEFEILCGILLDLMAEKCGE------DTKTDFKK
PanGbE1b     -KELQSIAGTASAAKLEGLSEVRAHGKKVLSALNDMVSQVDNMDALKAIIEPLGKKHAVELK-VDVKEFEILCGILLDLMAEKCGE------DTKTDFKK
```

```
PanGbE2a    -TQLQSVAETASAEEIAALAEVRAHGKKVFLALNDMVPHLNNVDALKETIAPLAKKHATELK-IDVKDFEIIFDNLLALIGEKQGA------DAKTAFKK
PanGbE2b    -TQLQSIAETASAEEIAALAEVRAHGKKVFSALNDMVSHLTNVDALKETINPLAKKHAAELK-VDVKDFRIIFENLLDLIGEKQGA------DAKTAFKK
PanGbE2c    -TQLQSVAETASAEEIAALAEVRAHGKKVFLALNDMVPHLTNVDALKETIAPLAKKHAAELK-VDVKDFRIIFENLLDLIGEKQGA------DAKTAFKK
PanGbX      -RDID------DIQQLQLSRELQAHGLRVMSFIEKSVARLQQEDKLQQLAFELGRCH-CRYN-ALPKYFEYVAFQFMTAVKPILKEK-WTS-EVDDAWKA
PanGbY      -KNID------TEEGIKESRQVRQHGRRVVMLLSKVIECLEDWDKTSTLLSELADRHQQHHK-VEVVNFKFLFAALNSVYIDVFGPT-FTP-DIEASWQK
PanHba1     --NID------P-----GSSYVRNQGGKIVTAIGTAVQNADNIQ---EALADLCCLHAYRIR-VDPVNFQYFSKCFLTVLAVHLQDD-FTA-DVHVVWDK
PanHba2     --DTS------A-----NSPQLSAHGAKIIASLGKAIKNVDNMS---AALSDLSDLHAQSIR-VDPSNFKYISHCLLVLLAAHLKAD-FTP-QVQIAWDK
PanHba3     -GDYK------A-----SSDKVIQHGKKVVDALVQASQHLHDLE---SQLHPLSVKHATELM-VDPVNFEHLSHCIHVTIAAHHGEK-YTA-EMHRSADK
PanHba4     -GDYK------A-----SSDKVIQHGKKVVDALVQASQHLHDLE---SHLHPLSEKHARELM-VDPVNFEHLSHCIHVTIAAHHGEK-YTA-EMHRSFDK
PanHbb1     -GNLS------TTSTIASNDRVQAHGAKVLTAIGDALVDLPNIK---QNLTDLSRLHSEILH-VDPENFRLLGQCLVIVLAAKFGAEKFNA-DVQAAWQK
PanHbb2     -GAMS------TLKDIESNPKVRAHGRKVMAAVGDAISHMDNIK---GHLSQLSQLHSDKLH-VDPANFELLGNNIVIVLAAKFGADAFTP-EVQATFQK
PanHbb3     -GDLS------TFSAIRQNPHVRTHGAKVLGAVVECLNHMDDIK---GHLAQLSILHSDTLH-VDPANFTLLGNCFLIVLAKSVGAG-FTP-DVHAACHK
PanHbb4     -GDLS------TISAIRKNSHVRAHGHKVLSAVGDCIPHLGDIK---GHLAQLSKLHCETLH-VDPANFCLLGKIIVVVLASHFGAK-FTP-EVQAAFQK
PanHbb5     -GDLS------TISAIRKNSHVRAHGHKVLSALGDCVPHLGDIK---GHLAQLSKLHCETLH-VDPANFCLLGQIITIVLASRFGAA-FTP-EVQACFEK
PanMb1      -RHLT------TEAEMRSNEDIRKHGNTVFTALGKLVKLKGNVE---GDLRSMADSHANKHK-IHLENFDIISKVIDNYFHESFPGD-YGA-DVQDYMKA
PanMb2      -RHLG------SEAEMRSCADLQKHGNTVFTALGKTLKLKGHHD---ADLRPMAESHSHKHK-IPVENFTLICSIIDKYLHESF-SD-YTG-DTRESLKS
PanMb3      -KDLA------SEAEMRGSDGLKNHGETVFTALGKALQQRDGIA---NELRPLAVTHSQNHK-IPLEEFENICEVIDVYLAEICPD--YAG-ETRTSVKA
PanMb4      -KDI-------PTSDLAVNADVKKHGGVVVDFLGKLLKLKGQND---SQLHTMAESHKNKHK-IPLDYFQVISSVIDVYVNENLPEE-YAP--VRQSMKS
PanMb5      -KDA-------PAADLPKNADVKKHGGVVVDFLGKLLKQKGHNE---SMLHTMAETHKNKHK-VLPDYFQLISSVIDVYVHENLPAE-YAP--VRDAMNA
PanMb6a     -KDI-------PVSELGNNADVKEQGAVVVKALGELLKLKGQHE---SQLHAMAESHKNTYK-IPVEYFPKIFKITDAYLQEKVGAA-YAA--IQAAMNV
PanMb6b     -KDT-------PVSELGNNADVKEQGAVVVKALGELLKLKGQHE---SQLHAMAESHKNTYK-IPVEYFPKIFKITDAYLHEKVGAV-YAA--IQAAMNV
PanMb7      -KDI-------PTSDLAANADVKKHGGVVVDFLGKLLKLKGQND---SQLHTMAESHKNKHR-IPLDYFQLISTVIDVYVYENLPGE-YGP--VRESLKA
PcaMb       -KHLK------TEAEMKASEDLKKHGVTVLTALGAILKKKGHHE---AELKPLAQSHATKHK-IPIKYLEFISEAIIHVLHSRHPGD-FGA-DAQGAMNK
PgeMb       -AGI-------AHGDLAGDAGVSAHGATVLNKLGDLLKARGGHA---ALLKPLSSSHATKHK-IPIINFKLIAEVIGKVMEEKAGLD---A-AGQTALRN
PhuGbE      -KGMD------SAEEMKQSDQVRGHGKKVFSAINDMVQHLGNSEAFLGIVTPLGKKHATQLK-IDPKNFRIICDIILQLMEEKFGG------DSKASFEK
PmaaHb1     -QGLT------SADQLKKSMDVRWHAERIINAVNDAVVAMDDTEKMSLKLRELSGKHAKSFQ-VDPQYFKVLAAVIVDTVL-----------PGDAGLEK
PmaaHb10    -KGLD------SADKLSKSPAVRWHAERIINAVNDSVVALDDPEKQSLKLKALSQKHAYEFH-VDSQYFKVLSATILEQVDDANGG--LSA-EGKSGWEK
PmaaHb11    -KGLD------SADQLSKSAAVRWHAERIINAVNDAVVALDDPEKQSLKLKALSKKHAQEFN-VDPQYFKVLSANVLEQVAAANGG--LSA-EAQGAWEK
PmaaHb12    -KGLD------SADQLKKSPAVRWHAERIINAVNDAVVALDDPPKLSLKLKALSKKHAQELN-VDPQYFKVLAGVISDAVAK-------SG-DEKAAVDK
PmaaHb13    -KGLD------SADQLKKSPAVRWHAERIINAVNDAVVALDDPAKQSLQLKALSQKHAHELN-VDPSYFKVLAGVISDAVAK-------SG-DAKAAVDK
PmaaHb14    -KGLD------SADKLKKSPAVRWHAERIINAVNDAVVALDDPAKQSLQLKALSQKHAHELN-VDPSYFKVLAGVISDAVAK-------SG-DAKAAVDK
PmaaHb2a    -KGLT------TADQLKKSADVRWHAERIINAVNDAVVSMDDTEKMSMKLRDLSGKHAKSFQ-VDPQYFKVLAAVIADTVA-----------AGDAGFEK
PmaaHb3     -KGLT------TADQLKKSADVRWHAERIINAVNDAVVSMDDTEKMSMKLGDLSGKHAKSFQ-VDPQYFKVLAAVIADTVA-----------AGDAGFEK
PmaaHb5a    -KGLT------TADQLKKSADVRWHAERIINAVNDAVASMDDTEKMSMKLRDLSGKHAKSFQ-VDPQYFKVLAAVIADTVA-----------AGDAGFEK
PmaaHb5b    -KGLT------TADQLKKSADVRWHAERIINAVNDAVASMDDTEKMSMKLRDLSGKHAKSFQ-VDPQYFKVLAAVIADTVA-----------AGDAGFEK
PmaaHb6     -KGLN------TAEELQNSAEVRIHGDKILAAVQQAVLDLDDPDKQKNKLKDLSKSHAQQFN-VEPAYFKTFAEVILKYVTETCGKS-FTS-EMRTSWSK
PmaaHb7     -KDLD------SEEKLKNSTAVRWHAERIINAVNDVIWLLDEPEKNAKNLKELSEKHAVQLN-VDAKFFKVLAELILDKVAEKSGDS-FSD-SARSAWEK
PmaaHb8     -KGMT------TADQLKKSADVRWHAERIINAVNDAVTSMDDTEKMSMKLRDLSGKHAKSFQ-VDPQYFKVLAAVIADTVA-----------AGDAGFEK
PmaaHb9     -KGLD------SADQLSKSAAVRWHAERIINAVNDAVVALDDPEKLSLKLKALSKKHAQEFN-VDPQYFKVLAVNIVEGVSSANGG--LGA-EAQAAWEK
PmaaMb1     -QALK------TADEMKSSPVLRDHAKRIMNSINDMVVALDDTNAQNAQMNGLSKKHANDFK-VDPKYFKVISNVILSVIAEGLGAQ-FND-AAKNGWSK
PmaaMb2     -KDIS------SQAELKSSAKVRDHAKRIMAFINDLVDNIDNAGAQTAKLHSLSAEHAEKFK-VDPQYFKVISNVLLDILGETFGAS-FSG-ATRSAWIK
PmaCygb     -KDLE------TADDLKASAKLRWHAGRVMGSLDKAVRSLRKPEELIKILRAVGLSHARKATPVDVKYYHILGGIIMDVLLETFKDE-LSP-TARSAWTK
PmaGbX1     -RDCE------DLQKLKMNKQLQAHGLRVMSFIEKSVARLEQECVLEQLIVEMGRKH-YKYN-ASPKYYSFVGIEFIATVQPFLQEK-WTN-EVEDAWQC
PsiCygb     -KHME------DPLEMERTPQLRKHARRVMGAVNTVVENINDSEKVSSVLALVGKAHALKHK-VEPVYFKFFTGVMLEVIAEEYAND-FTP-EVQRAWTK
PsiGbE      -KGMG------TAEEMEQSDQIRSHGKKVLTAINDLVQHLDSTDAFLGIVNPLGKKHATQLK-IDPKNFRVICDIILQLMEEKYGG------DCKASFEK
PsiGbX      -RDIE------DLQQLKMSKELQAHGLRVMSFIEKSVARLDQEDKLEHLAFELGRNH-YRYS-APPKYYEYVGVQFISAVQPILKER-WTP-ELQEAWQV
PsiGbY      -KSIP------TEGNLQEDPQIRYHGRRVMVALNQVIENLDNWKQACRILEHVAEKHKNTHH-VPAANFQSMFQVILSVCKELMGNE-FSS-EVSSAWEK
PsiHbaA     --DLH------H-----GSSQVRTQGKKVLSALGDAVAHVDDLP---SALSRLSDLHAKNLR-VDPVNFKLLSHCFLVVVALHHPSL-FTP-EVHVSLDK
PsiHbaD     --DLH------H-----DSEQVRHHGKKVVTALGNAVHHMDTLS---KTLSDLSDLHAYNLR-VDPVNFKLLSHCFQVVLAVHLRDE-YTP-QLHVAFDK
PsiHbB      -GNLS------SPTAILGNPKVRAHGKKVLTSFGEAVKNLDSVK---ATYAKLSELHCNKLH-VDPENFRLLGDILVIVLASHFGKE-FTP-ACQAAWQK
PsiHbG      -GNLS------NPQAIQHNPKVLEHGKKVLTSFGNAVKDLDHIK---ETFAHLSELHCEKLH-VDPENFKLLGNVLVIVLAAHIRKD-FPP-ACQAAWHK
PsiHbZ      --DLS------Q-----GSAQLHGHGSKVLSAIGEATKNIDNIT---GALATLSELHAYILR-VDPVNFKLLSHCILCSLAAHFPND-FTP-EVHAALDK
PsiMb       -KNLK------TADEMKSSDELKKHGITVLTALGRILKQKNNHE---QELKPLAESHATKHK-IPVKYLEFICEIIVKVIAEKHPAD-FGA-DSQAEMRK
PsiNgb      CRQFS------SPLECLSSPEFLDHIRKVMLVIDAAVTHLENLSSLEEYLTNLGKKH-QAVG-VKVDSFSAVGESLLFMLEKCLSTA-FSS-DVREAWTK
RnoCygb     -KHME------DPLEMERSPQLRKHACRVMGALNTVVENLHDPDKVSSVLALVGKAHALKHK-VEPMYFKILSGVILDVIAEEFAND-FPV-ETQKAWTK
RnoMb       -KNLK------SEEEMKSSEDLKKHGCTVLTALGTILKKKGQHA---AEIQPLAQSHATKHK-IPVKYLEFISEVIIQVLKKRYSGD-FGA-DAQGAMSK
SchMb       -TGI-------AQADMAGNAAISAHGATVLKKLGELLKAKGNHA---AILKPMANSHATKHK-IPINNFKLISEIIVKVMQEKAGMD---A-GGQQALRN
SjaMb       -AGI-------GLGDMAGNAAISAHGATVLKKLAEVLKAKGNHA---GIIKPLANSHATKHK-IAINNFKLITEIIVKVMQEKAGLD---A-GGQTALRN
TalaMb      -AGI-------AQADLAGNAAISAHGATVLKKLGELLKAKGSHA---SILKPMANSHATKHK-IPINNFKLISEVLVKVMQEKAGLD---A-GGQTALRN
TalbMb      -AGI-------AQADIAGNAAISAHGATVLKKLGELLKAKGSHA---AILKPLANSHATKHK-IPINNFKLISEVLVKVMHEKAGLD---A-GGQTALRN
TguCygb     -KHME------DPLEMERSLQLRKHARRVMGAINTVVENLNDSEKVSSVLALVGKAHALKHK-VEPIYFKKLTGVMLEVIAEEYPND-FTP-EAHGAWTK
TguGbE      -KGMD------SAEEMKQSDQVRGHGKKVFGAINDMVQHLDNSEAFLGIVTPLGKKHATQLK-IDPKNFRIICDIILQLMEEKFGG------DCKASFEK
TguHbA      --DLG------K-----GSAQVKGHGKKVAAALVEAANNVDDLA---GALSKLSDLHAQKLR-VDPVNFKLLGQCFLVVVATRNPSL-LTP-EVHASLDK
TguHbAD     --DMS------Q-----GSDQVRGHGKKVMAALSNAVKNLDNLS---QALSELSNLHAYNLR-VDPVNFKFLSQCLQVTLATRLGKE-YSP-EVHSAVDK
TguHbE      -GNMS------SPTAVLGNPMVRAHGKKVLTSFGEAVRNMDSVK---KCFAQLSKLHCEKLH-VDPENFRLLGDVLVVVLASHFGKE-FTP-ACQAAWQK
TguMb       -KGLK------TPDAMKGSEDLKKHGVTVLTQLGKILKAKGNHE---AELKPLAQTHATKHK-IPVKYLEFISEVIIKVLAEKHAAD-FGA-DAQAAMKK
TguNgb      CKQFA------SPHECLSAPEFLDHIRKVMLVIDAAVSHLENLSCLEEYLCNLGKKH-QAVG-VKVESFSTVGESLLYMREEKPGAA-FSP-EVQGGWSK
TniCygb1    -KHME------EPEEMQQSVQLRKHAHRVMTALNTLVESLDNSDRVASVLKSVGRAHALKHN-VDPKYFKILSGVILEVLGEAFTEI-ITA-EVASAWTK
TniGbX      -RDVE------DLERLRSSRELRAHGLRVMSFIEKSVARLDQQDRLEALAVELGKSH-YHYN-APPKYYSYVGAEFICAVQPILKER-FTS-ELEEAWKT
TniMb       -VGI-------AQSELAGNAAVSAHGATVLKKLGELLKAKGNHA---AILQPLANSHATKHK-IPIKNFKLIAEVIGKVMAEKAGLD---T-AGQQALRN
TniNgb      -TNCG------STQDCLSSPEFLEHVTKVMLVIDAAVSHLDDLHSLEDFLLNLGRKH-QAVG-VKPQSFAMVGESLLYMLQCSLGQA-YTA-SLRQAWLN
TruCygb1    -KDIE------EPEEMQRSIQLRKHAHRVMTTINTLVENLDDADAMASALKSVGRAHALRHK-VDPKYFKILSGVILEVLGEAFTEI-ITA-EVASAWTK
TruCygb2    -QDME------DPEEMERSSQLRHHACRVMNAINTVVENLHDPEKVSSVLALVGKAHAIKHK-VEPMYFKILCGVMLEVLCEAFPEF-FTA-DVRMVWAK
TruNgb      -TNCG------STQDCLSSPEFLEHVTKVMLVIDAAVSHLDDLHSLEDFLLNLGRKH-QAVG-VNPQSFATVGESLLYMLQCSLGQA-YTA-SLRQAWLN
TthMb       -AGI-------AQADIAGNAAVSAHGATVLKKLGELLKAKGSHA---AILKPLANSHATKHK-IPINNFKLISEVLVKVMHEKAGLD---A-GGQTALRN
XlaGbY      -KNIA------TLEEMQVNPGIRAHGKRVMGALNQVIQNLNDWEVVSSALTHLAQRHQDVHK-VGVNNFQLLFLVILTIFKEALGAD-FTP-EHCKSWEK
XlaHbA1     --DLS------H-----GSADLANHGGKVVNALGEAAKHINDLD---AALSTLSDLHAYNLR-VDPGNFKLLSHTIQVTLAIHFHKE-FDA-ATQAAWDK
XlaHbAT5    --DLS------H-----GSKDLRSHGGKVVKAIGNAATHIDDIP---HALSALSDLHAFKLK-VDPGNFKLLSHAIQVTLAIHFPAE-FNA-DAQAAWDK
XtrCygb     -KHME------DPLEMEGSVQLRKHARRVMGAVNSVVENLGDPEKITTVLSIVGKSHALKHK-VDPVYFKILTGVMLEVIAEEYAKD-FTP-DVQLAWNK
XtrGbX      -RDVD------DLQALRANKDLRAHGLRVLSFVEKSVARIADCARLEELALELGRSH-YRYN-APPRYYQYVGTEFISAVCPMLHDK-WTA-EVEEAWKG
XtrGbY      -KNIS------TLQEMQDNAGIRAHGKRVMGALNHVIENLKDWDAVCSALSHLAKRHQDVHK-VEVNNFELLFLVIISVFKEALGSG-FTP-EQSKSWEK
XtrHbA1     --DFS------E-----HSKHILAHGKKVSDALNEACNHLDNIA---GCLSKLSDLHAYDLR-VDPGNFPLLAHQILVVVAIHFPKQ-FDP-ATHKALDK
XtrHbB1     -GNLS------SIEAIFHNAAVATHGEKVLTSIGEAIKHMDDIK---GYYAQLSKYHSETLH-VDPYNFKRFCSCTIISMAQTLQED-FTP-ELQAAFEK
XtrHbB2     -GNLS------NVSAVSGNVKVKAHGNKVLSAVGSAIQHLDDVK---SHLKGLSKSHAEDLH-VDPENFKRLADVLVIVLAAKLGSA-FTP-QVQAVWEK
XtrNgb      SSHFS------KVQDCLSSAEFTEHIRKVMTVIDAAVSSLDCLSSLDEYLTSLGRKH-RAVG-VKLESFNTVGESLLFALESCLGDA-FTS-DTREAWSL
ZalGbE      -KGMD------SAEDMKQSDQVRGHGKKVFSAINDMVQHLDNSEAFLGIVTPLGKKHATQLK-IDPKNFRIICDIILQLMEEKFGG------DCKASFEK

AcaGbY      FFQVIQEEVEAAYDR-----------------------------------------
AcaMb       ALELFRNDMDRKYKELGFQGE-----------------------------------
AplGbE      VTNEICTHLNNVYKEAGW--------------------------------------
CanMb       VMAIIITDMEADYKELGFTE------------------------------------
CauMb1      VMEAVIGDIDTYYKEFGFAG------------------------------------
CauMb2      IMDCIIHDIDRYYKEIGFAG------------------------------------
CcaMb1      VMDVVIGDIDTYYKEIGFAG------------------------------------
CcaMb2      VMDCIIRDIDRYYKEIGFAG------------------------------------
CcrMb       ALELFRNDMAAKYKELGFQG------------------------------------
CliGbE      VTNEICSHLNNVYKEAGW--------------------------------------
CmiGbY      LFGVIYACLESHYKDAGFY-P-----------------------------------
CmyGbE      VTNEICTRLNNAYKEAGW--------------------------------------
CpiCygb     VKSLIYTHVTAAYKEVGWV-QYPNSTM-----------------------------
CpiGbE      VTNEICTRLNNAYKEAGW--------------------------------------
CpiGbX      GSSSAVGPLPCCRWQRSRP-----------------------RLGGLDP---FILP
CpiGbY      LFGLLSEQINASYVSTSKS-------------------------------------
```

```
CpiHbA       FLSAVGTVLTSKYR------------------------------------------
CpiHbaA      FLSAVGTVLTSKYR------------------------------------------
CpiHbaD      FLAAVSAVLAEKYR------------------------------------------
CpiHbB1      LVRVVAHALSYKYH------------------------------------------
CpiHbB2      LVSAVAHALALGYH------------------------------------------
CpiHbG       LAGVVAHALAHEYH------------------------------------------
CpiHbZ       FLSQISSVLTEKYR------------------------------------------
CpiMb        ALELFRNDMASKYKEFGFQG------------------------------------
CpiNgb       LYGSVVKAMSSGWDARKEG-E-----------------------------------
CraMb        VMAIIITDMEADYKELGFTE------------------------------------
CycrMb       ALELFRNDIAAKYKELGFHG------------------------------------
DreCygb1     LMGILYWQMNRVYAEVGWE----NSKK-----------------------------
DreCygb2     LMAALYWHITGAYTEVGWV-KLSSSAV-----------------------------
DreGbX       LFQYVTSIMREGFLEEERN-KRSNTQTSSRERPDKRSTAI----------------
DreHbAa      FFNNLALALSEKYR------------------------------------------
DreHbAe      FLSALALAMSEKYR------------------------------------------
DreHbAx      FLSAFSAVLAEKFR------------------------------------------
DreHbBa      FLAVVVSALCRQYH------------------------------------------
DreHbBe      FIAVAVSALGRQYH------------------------------------------
DreMb        VMDAVIGDIGGYYKEIGFAG------------------------------------
DreNgb       MYSIVVSAMTRGWAKNGEH-KSN---------------------------------
EpeMb        VMGIVIADLEANYKELGFTG------------------------------------
FalGbE       VTNEICTHLNNIYKEEGW--------------------------------------
FpeGbE       VTNEICTHLNNVYKEAGW--------------------------------------
FruMb        VMATIIADIDVTYKDLGFS-------------------------------------
GfoGbE       VTNEICTHLNNIYKEEGW--------------------------------------
GgaCygb      MRTLIYTHVTAAYKEAGWV-SYPSATL-----------------------------
GgaGbE       VTNEICTHLTNIYKEAGW--------------------------------------
GgaHbA       FLCAVGTVLTAKYR------------------------------------------
GgaHbAD      FLSAVSAVLAEKYR------------------------------------------
GgaHbG       LVRVVAHALARKYH------------------------------------------
GgaMb        ALELFRNDMASKYKEFGFQG------------------------------------
GgaNgb       LYSAVVKAMQRGWEVLPEG-D-----------------------------------
GgiMb        VMAVIIADMEADYKELGFTE------------------------------------
HsaCYGB      LRGLIYSHVTAAYKEVGWV-QQVPNATTPPATLPSSG------------------P
HsaHBA       FLASVSTVLTSKYR------------------------------------------
HsaHBB       VVAGVANALAHKYH------------------------------------------
HsaHBD       VVAGVANALAHKYH------------------------------------------
HsaHBE       LVSAVAIALAHKYH------------------------------------------
HsaHBG       MVTGVASALSSRYH------------------------------------------
HsaHBZ       FLSVVSSVLTEKYR------------------------------------------
HsaMB        ALELFRKDMASNYKELGFQG------------------------------------
HsaNGB       LYGAVVQAMSRGWDGE----------------------------------------
IpuGbX       LFQYVTWLMRRGYNEEEEEAKRNNTVGSSRERPRQRNTAL----------------
LchCygb      LMSIICCHVTATYKEVGWG-QLSNSTM-----------------------------
LchGbE       VTDVLCTHLNHAYKEAGW--------------------------------------
LchGbX1      LFKYLTAVMKKGYYVEEKK-ININNALYTKIPNALSSNPVQNNL------------
LchGbX2      LFKYVTEVMKRGYLQEEKI-KRTMETVEPKQASRRANNAI----------------
LchGbY       MLSFTYDYLVSCYHKSSGP-------------------------------------
LchHbA1      FLALISEILFSKYR------------------------------------------
LchHbA2      FLYEVEKALFETYR------------------------------------------
LchHbB1      LLSVVVAALSREYH------------------------------------------
LchHbB2      FMALVIDSLSRQYN------------------------------------------
LchMb        ALSMIIQDMGGMYKEFGFKG------------------------------------
LchNgb       LYSFVVETMARGWGANGEN-KLN---------------------------------
LpaGbE1      VTDLLYEEIKSTY-------------------------------------------
LpaGbE2a     AVDVIYENISAAY-------------------------------------------
LpaGbE2b     AVDEIYENIKAAY-------------------------------------------
LpaGbE2c     VMDVIYENIKAAY-------------------------------------------
LpaGbE2d     VMDVIYENLKAAY-------------------------------------------
LpaGbE2e     VMDVIYENIKAAY-------------------------------------------
LplaHbA      LLSQVVILLKSAY-------------------------------------------
LplaHbB      LMSMICILLRSSY-------------------------------------------
MangMb       ALELFRNDIATKYKELGFHG------------------------------------
MbiMb        ALELFRKDIAAKYKELGFHG------------------------------------
MgaGbE       VTNEICTHLTNIYKEAGW--------------------------------------
MglaHb2      VMTIICKMLKSKY-------------------------------------------
MmuMb        ALELFRNDIAAKYKELGFQG------------------------------------
MniMb        VMTTIIADIEANYKELGFTG------------------------------------
MunGbE       VTNEICTHLNNAYKEAGW--------------------------------------
NcoMb        VMAVIIADMEADYKELGFTE------------------------------------
OanGbY       LQSSLYDQVVITYLHSGSD-------------------------------------
OanHbW       LVGVVASALSSQYH------------------------------------------
OanMb        ALELFRNDMAAKYKEFGFQG------------------------------------
OanNgb       LYTAVVHAMSRGWGGE----------------------------------------
OlaCygb1     LLAILCCSIKAVYEELGWP-HLSNSTS-----------------------------
OlaCygb2     LMAAVYWHVTGAYTEVGWL-QVSSSAV-----------------------------
OlaHbA       FLAAVSLALSEKYR------------------------------------------
OlaHbB       FLAVVVSALGRQYH------------------------------------------
OlaMb        VMAGIIAEIEADYKELGFAG------------------------------------
OlaNgb       MYSIVVAAMSRGWAKNGED-KAD---------------------------------
PaeGbE1a     VTDVVCEQIKSTY-------------------------------------------
PaeGbE1b     VTDVVCEQIKSTY-------------------------------------------
PaeGbE1c     VTDVVCEQIKSTY-------------------------------------------
PaeGbE2a     VTDLIYEEIKAAY-------------------------------------------
PaeGbE2b     VTDLIYEEIKAAY-------------------------------------------
PaeGbE2c     VTDVMYEEIKAAY-------------------------------------------
PaeGbE2d     VTNLIYEEIKAAY-------------------------------------------
PanGbE1a     VTDVVCEQIKSTY-------------------------------------------
PanGbE1b     VTDVVCEQIKSTY-------------------------------------------
PanGbE2a     VTDLIYEEIKAAY-------------------------------------------
PanGbE2b     VTDLIYEEIKAAY-------------------------------------------
PanGbE2c     VTDLIYEEIKAAY-------------------------------------------
PanGbX       LFKYLISLMKKGFQEEEKT-HLINKSTYPKKQLISKMNFVKNNV------------
PanGbY       FYSLTYQQLEKCYSTCPSS-------------------------------------
PanHba1      FMCMLSCILAVKYR------------------------------------------
PanHba2      FLAAVSAVLTEKYR------------------------------------------
PanHba3      FFERVSSQLVSLYR------------------------------------------
PanHba4      GLECVSSELVSLYR------------------------------------------
PanHbb1      LMGVIAAGLSKQYH------------------------------------------
PanHbb2      LVAVISSALTREYH------------------------------------------
PanHbb3      LMVEIADGLSRQYH------------------------------------------
PanHbb4      LVAEVAAGLSRKYH------------------------------------------
```

```
PanHbb5      LVGVTAAGLSSQYH------------------------------------------
PanMb1       TLALIVQTLTKLYKELGK--------------------------------------
PanMb2       ALGGVCHSLEKLYKEV----------------------------------------
PanMb3       VLDVFSQSMTTLYGEV----------------------------------------
PanMb4       ALNQIANGLKDNYAKV----------------------------------------
PanMb5       ALKQIANTLKSNYAKV----------------------------------------
PanMb6a      AFDQIADGLKTQYQTV----------------------------------------
PanMb6b      AFDQIADGLKTQYQTV----------------------------------------
PanMb7       ALSQIANGLKANYAKV----------------------------------------
PcaMb        ALELFRKDIAAKYKELGYQG------------------------------------
PgeMb        VMAVIIADMEADYKELGFTE------------------------------------
PhuGbE       VTNEICTHLNNIYKEEGW--------------------------------------
PmaaHb1      LMSMICILLRSSY-------------------------------------------
PmaaHb10     LLSSICIHLKSAY-------------------------------------------
PmaaHb11     LLSIISILLKSQY-------------------------------------------
PmaaHb12     FLSQVVILLKFAY-------------------------------------------
PmaaHb13     FLSQVVILLKSAY-------------------------------------------
PmaaHb14     FLSQVVILLKSAY-------------------------------------------
PmaaHb2a     LMSMICILLRSAY-------------------------------------------
PmaaHb3      LMSMICILLRSAY-------------------------------------------
PmaaHb5a     LMSMICILLRSAY-------------------------------------------
PmaaHb5b     LMSMICILLRSAY-------------------------------------------
PmaaHb6      LLSLIIIELQSAY-------------------------------------------
PmaaHb7      LLTYICISLKVAY-------------------------------------------
PmaaHb8      LMSMICILLRSAY-------------------------------------------
PmaaHb9      FLSQVSILLKSQY-------------------------------------------
PmaaMb1      LLTTTCIGLKSAF-------------------------------------------
PmaaMb2      LLSIICIGLRSAF-------------------------------------------
PmaCygb      LLGTLCTEFENAYREEGVL-EQAAA-------------------------------
PmaGbX1      LFRYIAAVMKRGYLEEEAASNGVNTANYDRGQGNHGATAM----------------
PsiCygb      LKSLIYTHVTATYKEAD---REPSK-------------------------------
PsiGbE       VTNEICTRLNNAYKEAGW--------------------------------------
PsiGbX       GSGSAETSPSPG------------------------------------PLWFFIRP
PsiGbY       LFRLLFEQINTSYANASKS-------------------------------------
PsiHbaA      FMCAVSAVLTSKYR------------------------------------------
PsiHbaD      FMEAVCNVLTEKYR------------------------------------------
PsiHbB       LVGVVAHALAHQYH------------------------------------------
PsiHbG       LVAAVAHALALRYH------------------------------------------
PsiHbZ       FLSQISSVLTEKYR------------------------------------------
PsiMb        ALELFRNSMASKYKEFGFQG------------------------------------
PsiNgb       LYGAVVKAMSRGWDARKEG-E-----------------------------------
RnoCygb      LRGLIYSHVTAAYKEVGWV-QQVPNTTTLPATLPSSG------------------P
RnoMb        ALELFRNDIAAKYKELGFQG------------------------------------
SchMb        VMAAVIADLEANYKELGFSG------------------------------------
SjaMb        VMGVFIADMDANYKELGFSG------------------------------------
TalaMb       VMGIIIADLEANYKELGFTG------------------------------------
TalbMb       VMGIIIADLEANYKELGFSG------------------------------------
TguCygb      MKTLIYTHVTAAYKEVGWA-QYPTATL-----------------------------
TguGbE       VTNEICTHLNNIYKEEGW--------------------------------------
TguHbA       FLCAVGTVLTAKYR------------------------------------------
TguHbAD      FMSAVASVLAEKYR------------------------------------------
TguHbE       LVRVVAHALAHEYH------------------------------------------
TguMb        ALELFRNDMATKYKEFGFQG------------------------------------
TguNgb       LYNAVVKAMQRGWETLPEG-D-----------------------------------
TniCygb1     LLANMCCGIAAVYKEAGWT-ELSSSVE-----------------------------
TniGbX       LFQYVTGLMRKGHQEEGSR--QRHLALPPKDGPEKRTSAL----------------
TniMb        IMATIIADIDATYKELGFS-------------------------------------
TniNgb       MYSVVVASMSRGWAKNGED-KAD---------------------------------
TruCygb1     LLANMCCAVAAVYEEAGWT-KLSSSAE-----------------------------
TruCygb2     LMAAVYWHVTGAYTDVGWL-QVSSSAV-----------------------------
TruNgb       MYSVVVAAMSRGWAKNGED-KAD---------------------------------
TthMb        VMGIIIADLEANYKELGFSG------------------------------------
XlaGbY       LFSITYNFLDSCYTKSDS--------------------------------------
XlaHbA1      FLAEVATVLTSKYR------------------------------------------
XlaHbAT5     FLAVVSAVLVSKYR------------------------------------------
XtrCygb      LRSHLYSHVLSAYKEAGWT-QYPSNSV-----------------------------
XtrGbX       LFAYICTVMERGYQEEERR-HSDGRSLIDGLQGNKGLI------------------
XtrGbY       LFSITYKYLESCYANTDS--------------------------------------
XtrHbA1      FLVSVSNVLTSKYR------------------------------------------
XtrHbB1      LFAAIADALGKGYH------------------------------------------
XtrHbB2      LNATLVAALSHGYF------------------------------------------
XtrNgb       LYANVVQSMSRGWHRDSQE-QREGI-------------------------------
ZalGbE       VTNEICTYLNNIYKEEGW--------------------------------------
;
End;
begin mrbayes;
prset aamodel=fixed(GTR);
prset
aarevmatpr=fixed(4.1586,2.708,3.8656,24.35,10.1598,9.4882,20.2114,3.5106,1.4657,3.8675,5.2486,10.9961,2.4819,11.5206,4
6.2446,20.9301,1.7679,2.142,24.925,7.3554,1.2126,5.2294,3.5606,27.4689,3.8171,23.7387,1.2423,2.9529,61.886,4.7361,0.51
58,3.2531,8.395,5.6641,5.8071,3.0761,1.6717,49.6584,5.1728,5.2994,16.589,14.064,44.1125,1.8734,0.6694,20.9847,3.6294,0
.8758,1.5827,39.2126,19.572,0.4439,5.9873,0.8187,0.612,51.2992,5.1201,8.2657,9.0697,0.1046,0.1475,2.7681,0.2499,0.1704
,3.8588,12.1332,4.1661,0.2924,1.3217,0.3714,0.0342,0.8297,5.5689,6.2662,3.1366,5.811,0.1298,8.7426,10.8123,0.7374,27.2
397,11.1863,6.5557,11.402,19.1672,40.3888,3.4127,4.1467,0.433,0.6816,17.6791,1.6996,0.184,4.103,5.9867,5.9141,0.7616,1
.1743,2.3971,2.6214,47.0891,0.7127,5.698,31.6401,16.3622,0.3508,6.1073,11.9723,10.5666,2.3107,2.5174,2.0576,3.0472,0.0
852,0.433,2.9019,1.3651,0.8764,1.9268,17.0218,1.2701,2.6266,0.5349,0.7503,1.0652,3.5836,6.8211,4.3286,6.6732,4.9779,9.
685,5.7157,5.8408,51.9152,1.1643,40.5499,1.5561,41.8074,10.8855,0.7658,0.6271,10.1128,1.0923,2.2747,104.177,1.3451,61.
7519,25.3635,2.4365,1.7833,2.9635,6.0617,2.9314,16.6574,6.4234,0.234,3.8184,7.3241,11.1216,0.4882,1.2907,1.8118,17.597
6,0.9768,3.3942,19.7646,6.8105,4.7085,18.5746,0.9241,3.5396,1.6142,24.0373,76.3432,6.4046,13.0905,5.5905,0.9306,0.8767
,2.9006,63.3164,2.4345,3.9184,0.9623,1.3776,2.405,21.4061,30.8333,1.8539,2.439);
prset
statefreqpr=fixed(0.079066,0.055941,0.041977,0.053052,0.012937,0.071586,0.040767,0.057337,0.022355,0.062157,0.099081,0
.0646,0.022951,0.042302,0.04404,0.061197,0.053287,0.012066,0.034155,0.069147);
```

```
lset rates=gamma;
mcmc ngen=5000000 printfreq=1000 samplefreq=1000 nchains=4 savebrlens=yes;
End;
```

Supplemental Information Fig.3. Multiple sequence alignment (Nexus-format) of the globin sequences used for phylogenetic studies.

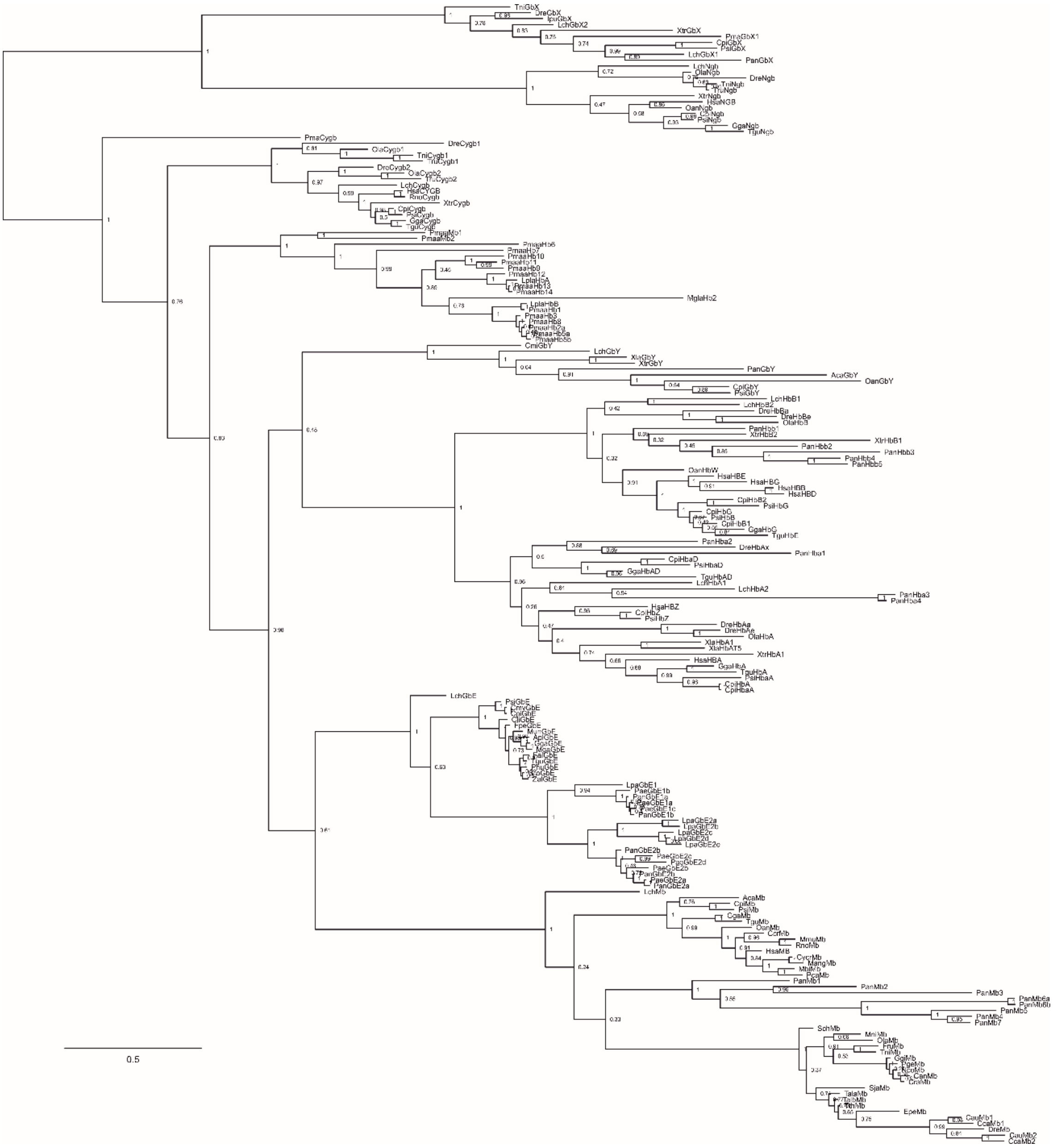

Supplemental Information Fig. 4. Bayesian phylogenetic tree of vertebrate globins. Tree reconstruction was carried out with the amino sequences assuming the LG model. The bar represents 0.1 PAM distance. The numbers at the nodes are posterior probabilities. For the abbreviations and accession numbers, see Supplemental Information Table 2. The full figure is available at https://figshare.com/s/860919619281ae184b37.

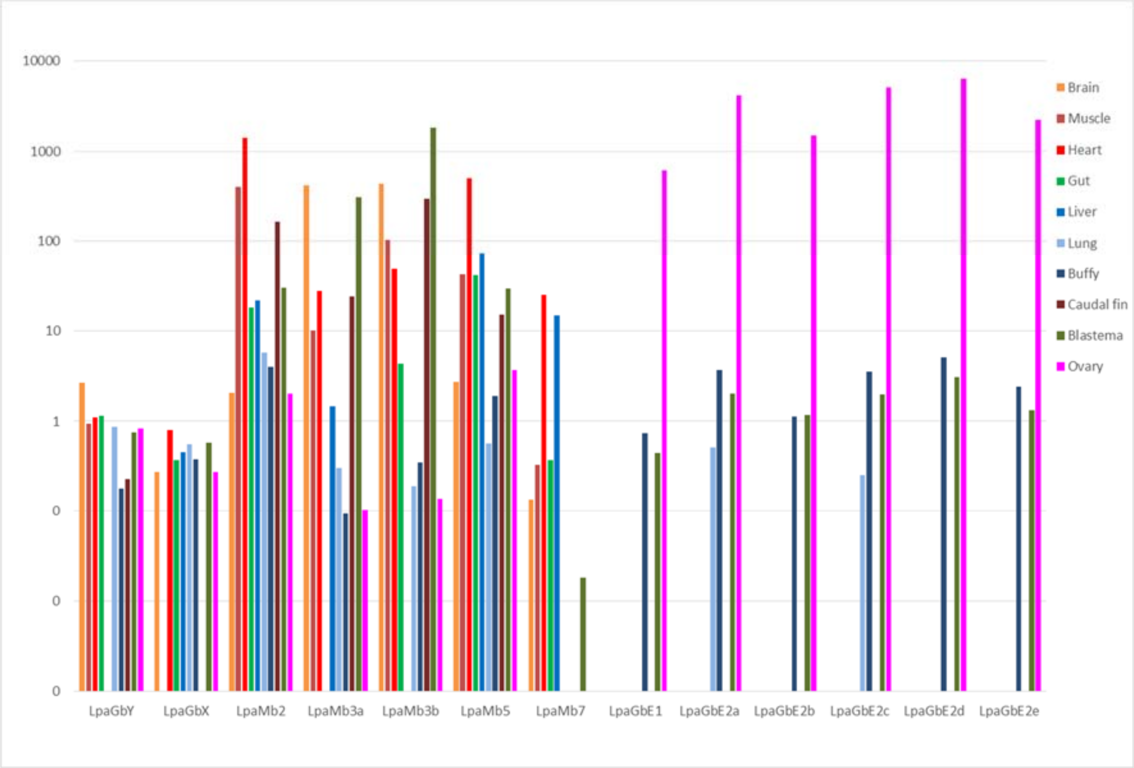

Supplemental Information Fig. 5. Expression of the *Mb* and *GbE* genes in selected *L. paradoxa* tissues, as estimated by RNA-Seq. The mRNA levels are displayed as RPKM in log-scale values. Transcriptome accession numbers are given in Supplemental Information Table 1, the copy numbers in Supplemental Information Table 3.

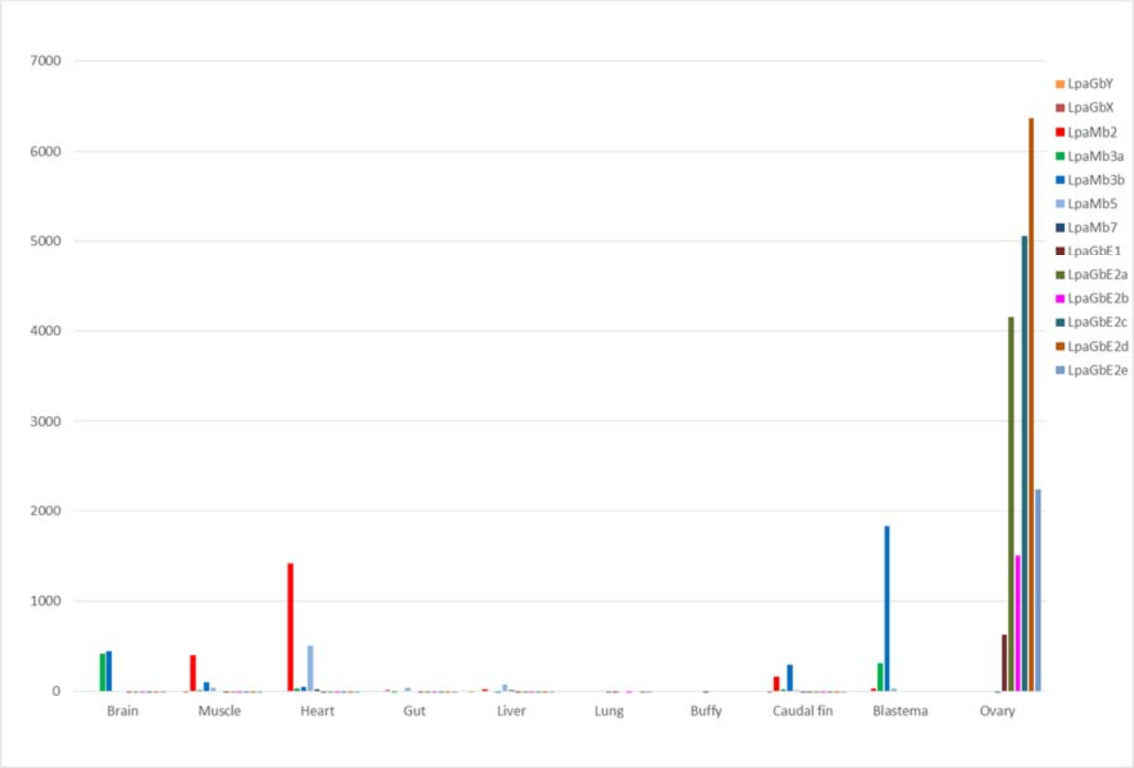

Supplemental Information Fig. 6. Tissue-specific expression of the *Mb* and *GbE* genes in *L. paradoxa*, as estimated by RNA-Seq. The mRNA levels are displayed as RPKM values. Transcriptome accession numbers are given in Supplemental Information Table 1, the copy numbers in Supplemental Information Table 3. Log-scale data are presented in Supplemental Information Fig. 7.

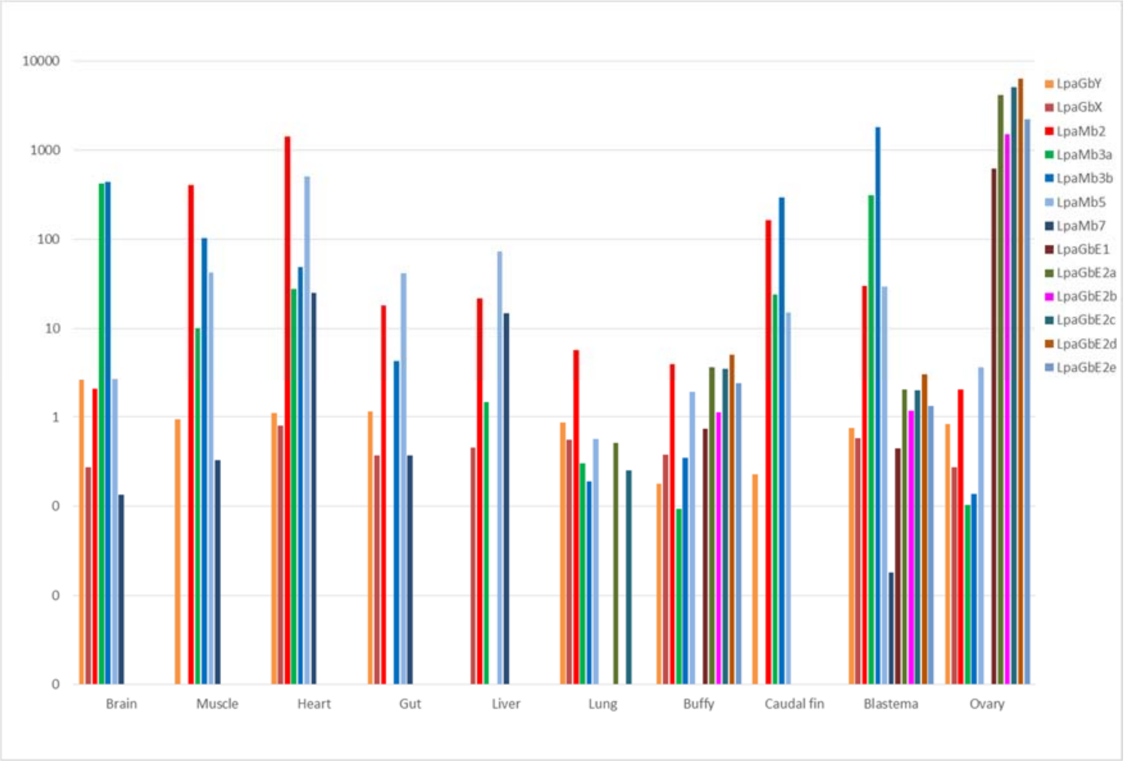

Supplemental Information Fig. 7. Tissue-specific expression of the *Mb* and *GbE* genes in *L. paradoxa*, as estimated by RNA-Seq. The mRNA levels are displayed as RPKM in log-scale values. Transcriptome accession numbers are given in Supplemental Information Table 1, the copy numbers in Supplemental Information Table 3.

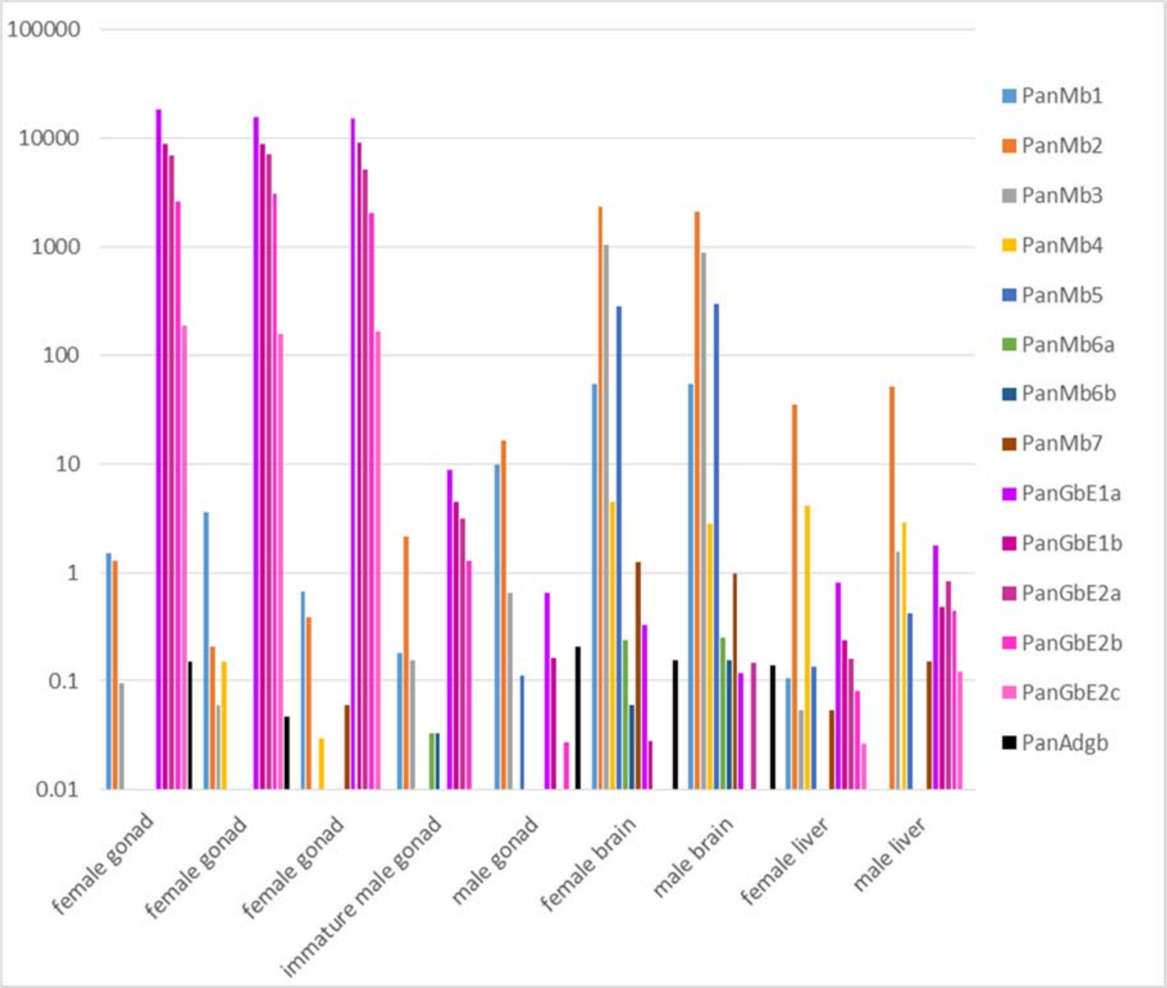

Supplemental Information Fig. 8. Expression of the *Mb* and *GbE* genes in selected *P. annectens* tissues, as estimated by RNA-Seq. The mRNA levels are displayed as RPKM values in log-scale. Transcriptome accession numbers are given in Table S1. Note the dominant expression of *GbE* in the ovary.

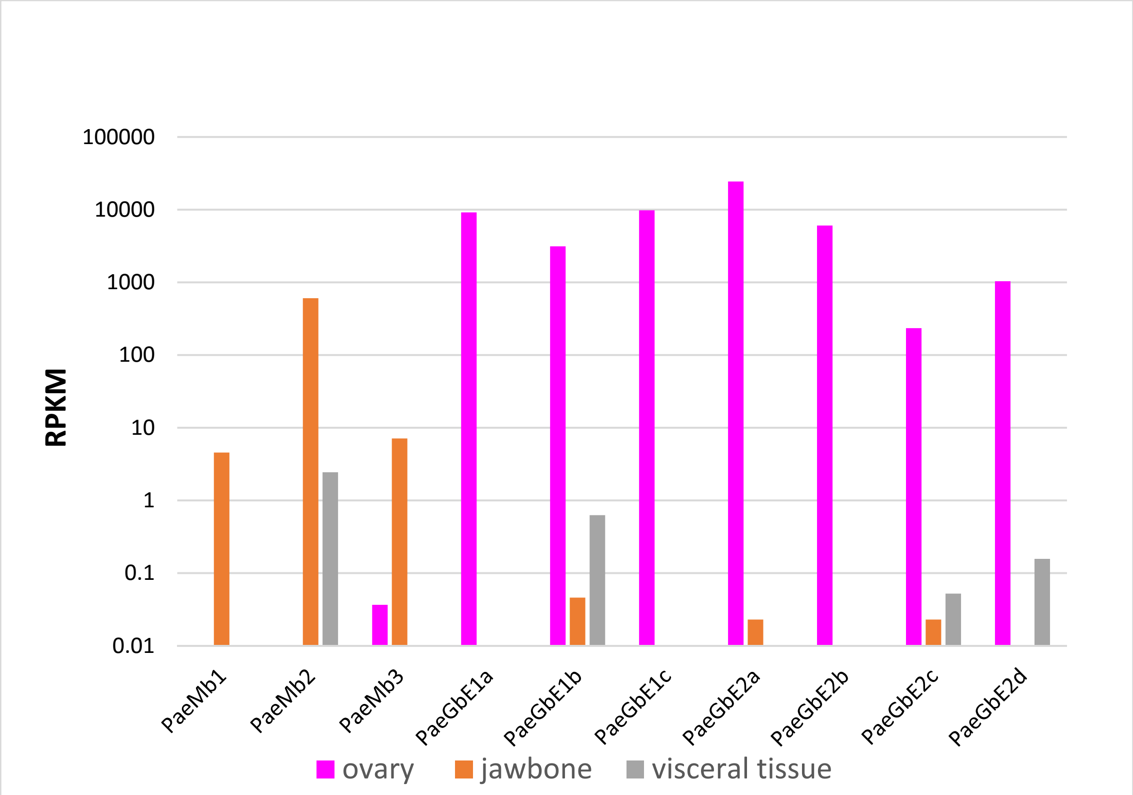

Supplemental Information Fig. 9. Expression of *Mb* and *GbE* genes in selected *P. aethiopicus* tissues, as estimated by RNA-Seq. The mRNA levels are displayed as log-scale RPKM values in log-scale. Transcriptome accession numbers are given in Supplemental Information Table 1.

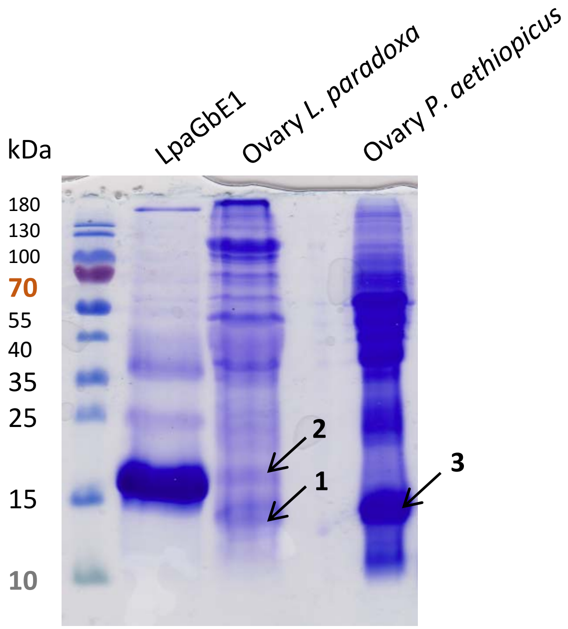

Supplemental Information Fig. 10. SDS-PAGE gel electrophoresis of recombinantly expressed GbE1 of *L. paradoxa* (lane 1) and proteins from the ovaries of *L. paradoxa* (lane 2) and *P. aethiopicus* (lane 4). The numbers indicate the bands excised for mass spectrometry.

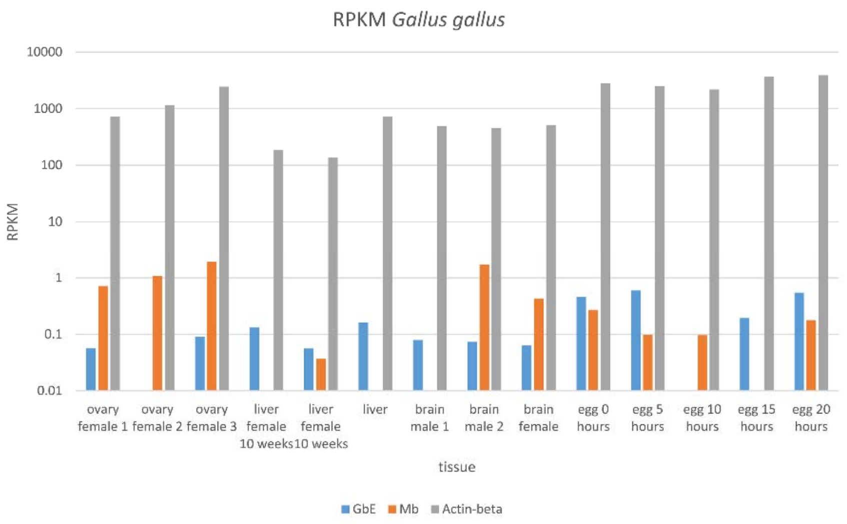

Supplemental Information Fig. 11. RNA-Seq of *Mb*, *GbE* and β-actin genes in chicken tissues. The mRNA levels are RPKM values in log-scale.

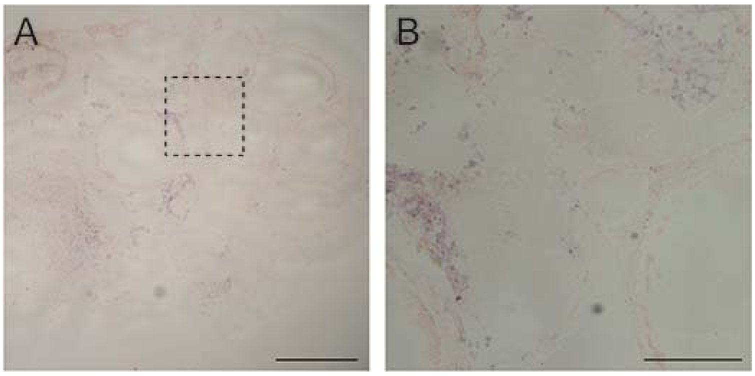

Supplemental Information Fig. 12. *In situ* hybridization of *GbE1* sense control probe. (A) sense probe showed no specific signal in *L. paradoxa* ovary sections. (B) zoom in of a region denoted in a dashed box in A. Scale bar: 2 mm (A) and 0.5 mm (B).

Supplemental Information Table 1. List of lungfish SRA data sets used in this study.

Run	Study	BioProject	BioSample	Experiment	Instrument	sex	tissue	species
SRR3632086	SRP076182	PRJNA317232	SAMN04601251	SRX1823854	Illumina HiSeq 2000	n.d.	muscle	*Lepidosiren paradoxa*
SRR3632085	SRP076182	PRJNA317232	SAMN04601250	SRX1823853	Illumina HiSeq 2000	n.d.	gut	*Lepidosiren paradoxa*
SRR3632084	SRP076182	PRJNA317232	SAMN04601249	SRX1823852	Illumina HiSeq 2000	n.d.	heart	*Lepidosiren paradoxa*
SRR3632083	SRP076182	PRJNA317232	SAMN04601248	SRX1823851	Illumina HiSeq 2000	n.d.	head	*Lepidosiren paradoxa*
SRR3632082	SRP076182	PRJNA317232	SAMN04601247	SRX1823850	Illumina HiSeq 2000	n.d.	brain	*Lepidosiren paradoxa*
SRR3632081	SRP076182	PRJNA317232	SAMN04601246	SRX1823849	Illumina HiSeq 2000	n.d.	gonad	*Lepidosiren paradoxa*
SRR3632080	SRP076182	PRJNA317232	SAMN04601245	SRX1823848	Illumina HiSeq 2000	n.d.	liver	*Lepidosiren paradoxa*
SRR3632079	SRP076182	PRJNA317232	SAMN04601244	SRX1823847	Illumina HiSeq 2000	n.d.	caudal fin	*Lepidosiren paradoxa*
SRR2895276	SRP065764	PRJNA301089	SAMN04235701	SRX1411327	Illumina HiSeq 2000	n.d.	blastema	*Lepidosiren paradoxa*
SRR2895273	SRP065764	PRJNA301089	SAMN04235701	SRX1411326	Illumina HiSeq 2000	n.d.	blastema	*Lepidosiren paradoxa*
SRR2895270	SRP065764	PRJNA301089	SAMN04235701	SRX1411325	Illumina HiSeq 2000	n.d.	blastema	*Lepidosiren paradoxa*
SRR2895265	SRP065764	PRJNA301089	SAMN04235701	SRX1411324	Illumina HiSeq 2000	n.d.	blastema	*Lepidosiren paradoxa*
SRR2895261	SRP065764	PRJNA301089	SAMN04235701	SRX1411322	Illumina HiSeq 2000	n.d.	blastema	*Lepidosiren paradoxa*
SRR2895255	SRP065764	PRJNA301089	SAMN04235701	SRX1411321	Illumina HiSeq 2000	n.d.	blastema	*Lepidosiren paradoxa*
SRR2895254	SRP065764	PRJNA301089	SAMN04235701	SRX1411314	Illumina HiSeq 2500	n.d.	blastema	*Lepidosiren paradoxa*
SRR1693787	SRP050575	PRJNA269317	SAMN03253061	SRX796494	Illumina HiSeq 2000	n.d.	kidney,lung,brain, spleen,heart,liver	*Lepidosiren paradoxa*
SRR7515656	SRP153183	PRJNA477294	SAMN04235701	SRX4385751	Illumina HiSeq 2500	female	ovary	*Lepidosiren paradoxa*
SRR8167646	SRP153183	PRJNA477294	SAMN04235701	SRX4988299	Illumina HiSeq 2500	n.d.	Buffy coat	*Lepidosiren paradoxa*
SRR8167645	SRP153183	PRJNA477294	SAMN04235701	SRX4988300	Illumina HiSeq 2500	n.d.	brain	*Lepidosiren paradoxa*
SRR8167644	SRP153183	PRJNA477294	SAMN04235701	SRX4988301	Illumina HiSeq 2500	n.d.	lung	*Lepidosiren paradoxa*
SRR505726	SRP013624	PRJNA164839	SAMN01041868	SRX152530	Illumina HiSeq 2000	n.d.	brain	*Protopterus annectens*
SRR505725	SRP013624	PRJNA164839	SAMN01041868	SRX152530	Illumina HiSeq 2000	n.d.	brain	*Protopterus annectens*
SRR505724	SRP013624	PRJNA164839	SAMN01041869	SRX152529	Illumina HiSeq 2000	n.d.	liver	*Protopterus annectens*
SRR505723	SRP013624	PRJNA164839	SAMN01041870	SRX152531	Illumina HiSeq 2000	n.d.	kidney	*Protopterus annectens*
SRR505722	SRP013624	PRJNA164839	SAMN01041870	SRX152531	Illumina HiSeq 2000	n.d.	kidney	*Protopterus annectens*
SRR505721	SRP013624	PRJNA164839	SAMN01041869	SRX152529	Illumina HiSeq 2000	n.d.	liver	*Protopterus annectens*

SRR2028027	SRP057960	PRJNA282925	SAMN03580842	SRX1016237	Illumina HiSeq 2000	male	gonad	*Protopterus annectens*
SRR2028021	SRP057960	PRJNA282925	SAMN03580841	SRX1016236	Illumina HiSeq 2000	female	liver	*Protopterus annectens*
SRR2028020	SRP057960	PRJNA282925	SAMN03580839	SRX1016234	Illumina HiSeq 2000	female	brain	*Protopterus annectens*
SRR2028017	SRP057960	PRJNA282925	SAMN03580843	SRX1016238	Illumina HiSeq 2000	male	gonad	*Protopterus annectens*
SRR2028000	SRP057960	PRJNA282925	SAMN03580840	SRX1016235	Illumina HiSeq 2000	male	liver	*Protopterus annectens*
SRR2027980	SRP057960	PRJNA282925	SAMN03580844	SRX1016241	Illumina HiSeq 2000	female	gonad	*Protopterus annectens*
SRR2027979	SRP057960	PRJNA282925	SAMN03580844	SRX1016240	Illumina HiSeq 2000	female	gonad	*Protopterus annectens*
SRR2027978	SRP057960	PRJNA282925	SAMN03580844	SRX1016239	Illumina HiSeq 2000	female	gonad	*Protopterus annectens*
SRR2027914	SRP057960	PRJNA282925	SAMN03580838	SRX1016233	Illumina HiSeq 2000	male	brain	*Protopterus annectens*
SRR1685666	SRP044127	PRJNA254214	SAMN02902907	SRX790602	Illumina HiSeq 2000	n.d.	liver	*Protopterus annectens*
SRR5114770	SRP095206	PRJNA357627	SAMN06146196	SRX2426781	Illumina HiSeq 1500	n.d.	developing jaw/ mandible	*Protopterus aethiopicus*
SRR5997828	SRP116672	PRJNA398732	SAMN07519109	SRX3153334	Illumina HiSeq 2000	n.d.	viscera mixture	*Protopterus aethiopicus*
SRR7240708	SRP044127	PRJNA473927	SAMN09288591	SRX4146489	Illumina NextSeq 500	female	ovary	*Protopterus aethiopicus*
SRR4897316	SRP092419	PRJNA352091	SAMN05966305	SRX2318355	Illumina HiSeq 2500	female	ovary	*Gallus gallus*
SRR5412257	SRP102989	PRJNA381064	SAMN06679865	SRX2704305	Illumina HiSeq 2500	female	ovary	*Gallus gallus*
SRR5412258	SRP102989	PRJNA381064	SAMN06679864	SRX2704306	Illumina HiSeq 2500	female	ovary	*Gallus gallus*
SRR6756988	SRP133195	PRJNA434773	SAMN08574267	SRX3729595	Illumina HiSeq 4000	female	liver	*Gallus gallus*
SRR6756992	SRP133195	PRJNA434773	SAMN08574268	SRX3729591	Illumina HiSeq 4000	female	liver	*Gallus gallus*
SRR6844900	SRP135813	PRJNA438519	SAMN08719874	SRX3800311	HiSeq X Ten	n.d.	liver	*Gallus gallus*
SRR5412243	SRP102989	PRJNA381064	SAMN06679879	SRX2704291	Illumina HiSeq 2500	male	brain	*Gallus gallus*
SRR5412244	SRP102989	PRJNA381064	SAMN06679878	SRX2704292	Illumina HiSeq 2500	male	brain	*Gallus gallus*
SRR5412242	SRP102989	PRJNA381064	SAMN06679880	SRX2704290	Illumina HiSeq 2500	female	brain	*Gallus gallus*
ERR753794	ERP009492	PRJEB8414	SAMEA3235032	ERX697515	Illumina Genome Analyzer IIx	n.d.	egg 0 hours	*Gallus gallus*
ERR753795	ERP009492	PRJEB8414	SAMEA3235033	ERX697516	Illumina Genome Analyzer IIx	n.d.	egg 5 hours	*Gallus gallus*
ERR753791	ERP009492	PRJEB8414	SAMEA3235029	ERX697512	Illumina Genome Analyzer IIx	n.d.	egg 10 hours	*Gallus gallus*
ERR753792	ERP009492	PRJEB8414	SAMEA3235030	ERX697513	Illumina Genome Analyzer IIx	n.d.	egg 15 hours	*Gallus gallus*

ERR753793	ERP009492	PRJEB8414	SAMEA3235031	ERX697514	Illumina Genome Analyzer IIx	n.d.	egg 20 hours	*Gallus gallus*

Supplemental Information Table 2. List of globin sequences used in this study. The EMBL/GenBank accession numbers of the nucleotides are given, if available.

**Abbreviation**	**Species**	**common name**	**accession no.**
AcaGbY	*Anolis carolinensis*	anole lizard	NW_003341080
AcaMb	*Anolis carolinensis*	green anole	XM_003220932
AplGbE	*Anas platyrhynchos*	mallard	XM_005012200
CanMb	*Cryodraco antarcticus*	long-fingered icefish	U71056
CauMb1	*Carassius auratus*	goldfish	AM747267
CauMb2	*Carassius auratus*	goldfish	AM747268
CcaMb1	*Cyprinus carpio*	common carp	KC342292
CcaMb2	*Cyprinus carpio*	common carp	DQ338464
CcrMb	*Condylura cristata*	star-nosed mole	NM_001287785
CliGbE	*Columba livia*	common pigeon	XM_005507829
CmiGbY	*Callorhinchus milii*	elephant shark	NM_001292790
CmyGbE	*Chelonia mydas*	green sea turtle	XM_007064067
CpiCygb	*Chrysemys picta bellii*	Western painted turtle	XM_005297540
CpiGbE	*Chrysemys picta bellii*	Western painted turtle	XM_005293060
CpiGbX	*Chrysemys picta bellii*	Western painted turtle	XM_005293187
CpiGbY	*Chrysemys picta bellii*	Western painted turtle	XM_005306194
CpiHbaA	*Chrysemys picta bellii*	Western painted turtle	XM_005306158
CpiHbaD	*Chrysemys picta bellii*	Western painted turtle	XM_005306157
CpiHbB1	*Chrysemys picta bellii*	Western painted turtle	XM_005290010
CpiHbB2	*Chrysemys picta bellii*	Western painted turtle	XM_005290009
CpiHbG	*Chrysemys picta bellii*	Western painted turtle	XM_005290008
CpiHbZ	*Chrysemys picta bellii*	Western painted turtle	XM_005306148
CpiMb	*Chrysemys picta bellii*	Western painted turtle	XM_005300826

CpiNgb	*Chrysemys picta bellii*	Western painted turtle	XM_005301643
CraMb	*Chionodraco rastrospinosus*	ocellated icefish	U70871
CycrMb	*Cystophora cristata*	hooded seal	KC524751
DreCygb1	*Danio rerio*	zebrafish	BC165894
DreCygb2	*Danio rerio*	zebrafish	AJ635229
DreGbX	*Danio rerio*	zebrafish	AJ635194
DreHbAa	*Danio rerio*	zebrafish	AY325264
DreHbAe	*Danio rerio*	zebrafish	BC164447
DreHbAx	*Danio rerio*	zebrafish	AL915033
DreHbBa	*Danio rerio*	zebrafish	BC164283
DreHbBe	*Danio rerio*	zebrafish	NM_001097585
DreMb	*Danio rerio*	zebrafish	AY337025
DreNgb	*Danio rerio*	zebrafish	BC059416
EpeMb	*Euthynnus pelamis*	skipjack tuna	AF291837
FalGbE	*Ficedula albicollis*	collared flycatcher	XM_005039642
FpeGbE	*Falco peregrinus*	peregrine falcon	XM_005237389
GfoGbE	*Geospiza fortis*	medium ground-finch	XM_005421887
GgaCygb	*Gallus gallus*	chicken	NM_001008789
GgaGbE	*Gallus gallus*	chicken	NM_001008786
GgaHbA	*Gallus gallus*	chicken	NM_001004376
GgaHbAD	*Gallus gallus*	chicken	CR338842
GgaHbG	*Gallus gallus*	chicken	M73995
GgaMb	*Gallus gallus*	chicken	XM_003202347
GgaNgb	*Gallus gallus*	chicken	NM_001031551
GgiMb	*Gobionotothen gibberifron*	humped rockcod	U71057
HsaCYGB	*Homo sapiens*	man	AJ315162
HsaHBA	*Homo sapiens*	man	AF105974

HsaHBB	*Homo sapiens*	man	NM_000518
HsaHBD	*Homo sapiens*	man	NM_000519
HsaHBE	*Homo sapiens*	man	NM_005330
HsaHBG	*Homo sapiens*	man	NM_000559
HsaHBZ	*Homo sapiens*	man	M24173
HsaMB	*Homo sapiens*	man	NM_203377
HsaNGB	*Homo sapiens*	man	AB463927
IpuGbX	*Ictalurus punctatus*	channel catfish	CK416201
LchCygb	*Latimeria chalumnae*	coelacanth	XM_005993205
LchGbE	*Latimeria chalumnae*	coelacanth	XM_006011781
LchGbX1	*Latimeria chalumnae*	coelacanth	XM_005987904
LchGbX2	*Latimeria chalumnae*	coelacanth	XM_006012915
LchGbY	*Latimeria chalumnae*	coelacanth	XM_005990799
LchHbA1	*Latimeria chalumnae*	coelacanth	XM_006011045
LchHbA2	*Latimeria chalumnae*	coelacanth	XM_006011046
LchHbB1	*Latimeria chalumnae*	coelacanth	XM_006011047
LchHbB2	*Latimeria chalumnae*	coelacanth	XM_006011048
LchMb	*Latimeria chalumna*	coelacanth	XM_006011146
LchNgb	*Latimeria chalumnae*	coelacanth	XM_005986619
LpaGbE1	*Lepidosiren paradoxa*	South American lungfish	LR030441
LpaGbE2a	*Lepidosiren paradoxa*	South American lungfish	LR030442
LpaGbE2b	*Lepidosiren paradoxa*	South American lungfish	LR030443
LpaGbE2c	*Lepidosiren paradoxa*	South American lungfish	LR030444
LpaGbE2d	*Lepidosiren paradoxa*	South American lungfish	LR030445
LpaGbE2e	*Lepidosiren paradoxa*	South American lungfish	LR030446
LplaHbA	*Lampetra zanandreai*	Po brook lamprey	Z24746
LplaHbB	*Lampetra zanandreai*	Po brook lamprey	Z24747

MangMb	*Mirounga angustirostris*	Northern elephant seal	KC524754
MbiMb	*Mesoplodon bidens*	Sowerby's beaked whale	KC524765
MgaGbE	*Meleagris gallopavo*	turkey	XM_003202331
MglaHb2	*Myxine glutinosa*	Atlantic hagfish	AF157494
MmuMb	*Mus musculus*	mouse	AK137456
MniMb	*Makaira nigricans*	Atlantic blue marlin	AF291833
MunGbE	*Melopsittacus undulatus*	budgerigar	XM_005150295
NcoMb	*Notothenia coriiceps*	black rockcod	NM_001303294
OanGbY	*Ornithorhynchus anatinus*	platypus	genome
OanHbW	*Ornithorhynchus anatinus*	platypus	genome
OanMb	*Ornithorhynchus anatinus*	platypus	XM_001513063
OanNgb	*Ornithorhynchus anatinus*	platypus	XM_001508367
OlaCygb1	*Oryzias latipes*	medaka	NM_001104767
OlaCygb2	*Oryzias latipes*	medaka	NM_001104768
OlaHbA	*Oryzias latipes*	medaka	BAC20295
OlaHbB	*Oryzias latipes*	medaka	AB080120
OlaMb	*Oryzias latipes*	medaka	BJ883657
OlaNgb	*Oryzias latipes*	medaka	DK002855
PaeGbE1a	*Protopterus aethiopicus*	marbled lungfish	LR030452
PaeGbE1b	*Protopterus aethiopicus*	marbled lungfish	LR030453
PaeGbE1c	*Protopterus aethiopicus*	marbled lungfish	LR030454
PaeGbE2a	*Protopterus aethiopicus*	marbled lungfish	LR030455
PaeGbE2b	*Protopterus aethiopicus*	marbled lungfish	LR030456
PaeGbE2c	*Protopterus aethiopicus*	marbled lungfish	LR030457
PaeGbE2d	*Protopterus aethiopicus*	marbled lungfish	LR030458
PanGbE1a	*Protopterus annectens*	West African lungfish	LR030447
PanGbE1b	*Protopterus annectens*	West African lungfish	LR030448

PanGbE2a	*Protopterus annectens*	West African lungfish	LR030449
PanGbE2b	*Protopterus annectens*	West African lungfish	LR030450
PanGbE2c	*Protopterus annectens*	West African lungfish	LR030451
PanGbX	*Protopterus annectens*	West African lungfish	TBA
PanGbY	*Protopterus annectens*	West African lungfish	TBA
PanHba1	*Protopterus annectens*	West African lungfish	TBA
PanHba2	*Protopterus annectens*	West African lungfish	TBA
PanHba3	*Protopterus annectens*	West African lungfish	TBA
PanHba4	*Protopterus annectens*	West African lungfish	TBA
PanHbb1	*Protopterus annectens*	West African lungfish	TBA
PanHbb2	*Protopterus annectens*	West African lungfish	TBA
PanHbb3	*Protopterus annectens*	West African lungfish	TBA
PanHbb4	*Protopterus annectens*	West African lungfish	TBA
PanHbb5	*Protopterus annectens*	West African lungfish	TBA
PanMb1	*Protopterus annectens*	West African lungfish	LT604990
PanMb2	*Protopterus annectens*	West African lungfish	LT604991
PanMb3	*Protopterus annectens*	West African lungfish	LT604992
PanMb4	*Protopterus annectens*	West African lungfish	LT604993
PanMb5	*Protopterus annectens*	West African lungfish	LT604994
PanMb6a	*Protopterus annectens*	West African lungfish	LT604995
PanMb6b	*Protopterus annectens*	West African lungfish	LT604996
PanMb7	*Protopterus annectens*	West African lungfish	LT604997
PcaMb	*Physeter catodon*	sperm whale	NM_001290722
PgeMb	*Pseudochaenichthys georgianus*	South Georgia icefish	U71055
PhuGbE	*Pseudopodoces humilis*	Tibetan ground-tit	XM_014256596
PmaaHb1	*Petromyzon marinus*	sea lamprey	P09967

PmaaHb10	*Petromyzon marinus*	sea lamprey	FD718926
PmaaHb11	*Petromyzon marinus*	sea lamprey	ENSPMAG00000001592
PmaaHb12	*Petromyzon marinus*	sea lamprey	genome
PmaaHb13	*Petromyzon marinus*	sea lamprey	genome
PmaaHb14	*Petromyzon marinus*	sea lamprey	genome
PmaaHb2a	*Petromyzon marinus*	sea lamprey	Q9I9I3
PmaaHb3	*Petromyzon marinus*	sea lamprey	P09968
PmaaHb5a	*Petromyzon marinus*	sea lamprey	P02208
PmaaHb5b	*Petromyzon marinus*	sea lamprey	ENSPMAG00000005354
PmaaHb6	*Petromyzon marinus*	sea lamprey	EG333697
PmaaHb7	*Petromyzon marinus*	sea lamprey	EE278870
PmaaHb8	*Petromyzon marinus*	sea lamprey	ENSPMAG00000005367
PmaaHb9	*Petromyzon marinus*	sea lamprey	ENSPMAG00000008540
PmaaMb1	*Petromyzon marinus*	sea lamprey	ENSPMAG00000006056
PmaaMb2	*Petromyzon marinus*	sea lamprey	EG021442
PmaCygb	*Petromyzon marinus*	sea lamprey	genome
PmaGbX1	*Petromyzon marinus*	sea lamprey	ENSPMAG00000007241
PsiCygb	*Pelodiscus sinensis*	Chinese soft-shelled turtle	XM_006136486
PsiGbE	*Pelodiscus sinensis*	Chinese soft-shelled turtle	XM_006127809
PsiGbX	*Pelodiscus sinensis*	Chinese soft-shelled turtle	XM_006131430
PsiGbY	*Pelodiscus sinensis*	Chinese soft-shelled turtle	XM_006124603
PsiHbaA	*Pelodiscus sinensis*	Chinese soft-shelled turtle	ENSPSIG00000011874
PsiHbaD	*Pelodiscus sinensis*	Chinese soft-shelled turtle	ENSPSIG00000012157
PsiHbB	*Pelodiscus sinensis*	Chinese soft-shelled turtle	XM_006130979
PsiHbG	*Pelodiscus sinensis*	Chinese soft-shelled turtle	XM_006130980
PsiHbZ	*Pelodiscus sinensis*	Chinese soft-shelled turtle	XM_006124534
PsiMb	*Pelodiscus sinensis*	Chinese soft-shelled turtle	XM_006137956

PsiNgb	*Pelodiscus sinensis*	Chinese soft-shelled turtle	XM_006117796
RnoCygb	*Rattus norvegicus*	rat	NM_130744
RnoMb	*Rattus norvegicus*	Norway rat	AF197916
SchMb	*Sarda chiliensis*	Pacific bonito	AF291834
SjaMb	*Scomber japonicus*	chub mackerel	AF291835
TalaMb	*Thunnus alalunga*	albacore	AF291832
TalbMb	*Thunnus albacares*	yellowfin tuna	AF291838
TguCygb	*Taeniopygia guttata*	zebra finch	XM_002195407
TguGbE	*Taeniopygia guttata*	zebra finch	XM_002196350
TguHbA	*Taeniopygia guttata*	zebra finch	DQ216719
TguHbAD	*Taeniopygia guttata*	zebra finch	DQ213486
TguHbE	*Taeniopygia guttata*	zebra finch	NM_001245112
TguMb	*Taeniopygia guttata*	zebra finch	XM_002199380
TguNgb	*Taeniopygia guttata*	zebra finch	XM_003962460
TniCygb1	*Tetraodon nigroviridis*	green spotted puffer	AJ635230
TniGbX	*Tetraodon nigroviridis*	green spotted puffer	AJ635193
TniMb	*Tetraodon nigroviridis*	spotted green pufferfish	CR660178
TniNgb	*Tetraodon nigroviridis*	green spotted puffer	AJ315609
TruCygb1	*Takifugu rubripes*	torafugu	XM_003964492
TruCygb2	*Takifugu rubripes*	torafugu	AJ635231
TruMb	*Takifugu rubripes*	torafugu	XM_003976046
TruNgb	*Takifugu rubripes*	torafugu	genome
TthMb	*Thunnus thynnus orientalis*	Pacific bluefin tuna	AF291836
XlaGbY	*Xenopus laevis*	African clawed frog	NM_001095686
XlaHbA1	*Xenopus laevis*	African clawed frog	X02796
XlaHbAT5	*Xenopus laevis*	African clawed frog	X02798
XtrCygb	*Xenopus tropicalis*	Western clawed frog	AJ635232

XtrGbX	*Xenopus tropicalis*	Western clawed frog	NM_001011196
XtrGbY	*Xenopus tropicalis*	Western clawed frog	XM_002941194
XtrHbA1	*Xenopus tropicalis*	Western clawed frog	BC088005
XtrHbB1	*Xenopus tropicalis*	Western clawed frog	NM_203528
XtrHbB2	*Xenopus tropicalis*	Western clawed frog	NM_001016495
XtrNgb	*Xenopus tropicalis*	Western clawed frog	NM_001030351
ZalGbE	*Zonotrichia albicollis*	white-throated sparrow	XM_005488706

Supplemental Information Table 3. RPKM of *GbY*, *GbX*, *Mb* and *GbE* genes in the transcriptomes from different lungfish (*L. paradoxa*) tissues used for expression estimation by RNA-seq.

	SRR8167645	SRR3632086	SRR3632084	SRR3632085	SRR3632080	SRR8167644	SRR8167646	SRR3632079	SRR2895254	SRR7515656
	**Brain**	**Muscle**	**Heart**	**Gut**	**Liver**	**Lung**	**Buffy**	**Caudal fin**	**Blastema**	**Ovary**
LpaGbY	2.63	0.93	1.11	1.16	0.00	0.88	0.18	0.23	0.75	0.84
LpaGbX	0.27	0.00	0.79	0.37	0.45	0.56	0.37	0.00	0.57	0.27
LpaMb2	2.09	404.68	1412.10	17.99	21.92	5.69	3.99	166.20	30.26	2.03
LpaMb3a	419.21	10.15	27.73	0.00	1.47	0.30	0.09	24.21	310.19	0.10
LpaMb3b	442.74	104.72	48.89	4.29	0.00	0.19	0.35	299.39	1826.12	0.14
LpaMb5	2.70	42.23	503.26	41.82	73.22	0.56	1.90	15.00	29.38	3.64
LpaMb7	0.13	0.33	25.12	0.37	14.95	0.00	0.00	0.00	0.02	0.00
LpaGbE1	0.00	0.00	0.00	0.00	0.00	0.00	0.73	0.00	0.44	623.12
LpaGbE2a	0.00	0.00	0.00	0.00	0.00	0.51	3.70	0.00	2.04	4153.06
LpaGbE2b	0.00	0.00	0.00	0.00	0.00	0.00	1.13	0.00	1.18	1498.12
LpaGbE2c	0.00	0.00	0.00	0.00	0.00	0.25	3.49	0.00	2.01	5061.83
LpaGbE2d	0.00	0.00	0.00	0.00	0.00	0.00	5.07	0.00	3.05	6364.72
LpaGbE2e	0.00	0.00	0.00	0.00	0.00	0.00	2.40	0.00	1.32	2241.18

Supplemental Information Table 4. RPKM of *Mb*, *GbE* and *Adgb* genes in the transcriptomes from different lungfish (*P. annectens*) tissues used for expression estimation by RNA-seq.

	SRR2027978	SRR2027979	SRR2027980	SRR2028027	SRR2028017	SRR2028020	SRR2027914	SRR2028021	SRR2028000
	**female gonad**	**female gonad**	**female gonad**	**immature male gonad**	**male gonad**	**female brain**	**male brain**	**female liver**	**male liver**
PanMb1	1.51	3.58	0.67	0.18	9.78	53.59	54.25	0.10	0.00
PanMb2	1.27	0.21	0.39	2.15	16.46	2378.25	2106.90	34.89	51.16
PanMb3	0.10	0.06	0.00	0.15	0.64	1039.06	891.26	0.05	1.56
PanMb4	0.00	0.15	0.03	0.00	0.00	4.47	2.80	4.15	2.88
PanMb5	0.00	0.00	0.00	0.00	0.11	282.55	298.36	0.13	0.43
PanMb6a	0.00	0.00	0.00	0.03	0.00	0.24	0.25	0.00	0.00
PanMb6b	0.00	0.00	0.00	0.03	0.00	0.06	0.16	0.00	0.00
PanMb7	0.00	0.00	0.06	0.00	0.00	1.26	0.97	0.05	0.15
PanGbE1a	18509.96	15819.15	15249.05	8.83	0.66	0.33	0.12	0.80	1.80
PanGbE1b	8990.94	8922.64	9225.27	4.49	0.16	0.03	0.00	0.24	0.48
PanGbE2a	6927.00	7200.72	5242.37	3.09	0.00	0.00	0.15	0.16	0.84
PanGbE2b	2658.76	3124.96	2035.23	1.27	0.03	0.00	0.00	0.08	0.45
PanGbE5	187.95	156.17	163.98	0.00	0.00	0.00	0.00	0.03	0.12
PanAdgb	0.15	0.05	0.00	0.00	0.21	0.16	0.14	0.00	0.00

Supplemental Information Table 5. RPKM of *GbY*, *GbX*, *Mb*, *GbE* and *Hb* genes in the transcriptomes from different lungfish (*P. aethiopicus*) tissues used for expression estimation by RNA-seq.

	SRR7240708	SRR5114770	SRR5997828
	**Ovary**	**Jawbone**	**Viscera**
PaeGbY	0.10	6.79	0.20
PaeGbX	0.10	1.04	0.29
PaeMb1	0.00	4.54	0.00
PaeMb2	0.00	603.20	2.43
PaeMb3	0.04	7.11	0.00
PaeGbE1a	9138.16	0.00	0.00
PaeGbE1b	3126.12	0.05	0.63
PaeGbE1c	9759.73	0.00	0.00
PaeGbE2a	24330.54	0.02	0.00
PaeGbE2b	6036.05	0.00	0.00
PaeGbE2c	234.41	0.02	0.05
PaeGbE2d	1036.76	0.00	0.16
PaeHba	0.00	0.00	0.00
PaeHbb3	15.97	283.66	671.21
PaeHbb4	0.00	0.00	0.00
PaeHbb5	50.73	619.42	1132.24

Supplemental Information Table 6. RPKM of *GbE, Mb* and *β-actin* genes in the transcriptomes from different chicken (*Gallus gallus*) tissues used for expression estimation by RNA-seq.

	SRR5412257 **ovary female 1**	SRR5412258 **ovary female 2**	SRR4897316 **ovary female 3**	SRR6756988 **liver female 10 weeks**	SRR6756992 **liver female 10 weeks**	SRR6844900 **liver**	SRR5412243 **brain male 1**	SRR5412244 **brain male 2**	SRR5412242 **brain female 2**	ERR753794 **egg 0 hours**	ERR753795 **egg 5 hours**	ERR753791 **egg 10 hours**	ERR753792 **egg 15 hours**	ERR753793 **egg 20 hours**
GgaGbE	0.06	0.00	0.09	0.13	0.06	0.16	0.08	0.07	0.06	0.46	0.60	0.00	0.19	0.55
GgaMb	0.72	1.09	1.92	0.00	0.04	0.00	0.00	1.73	0.43	0.27	0.10	0.10	0.00	0.18
Gga β-actin	727.04	1156.21	2447.47	184.04	137.77	733.95	490.71	453.66	514.02	2803.80	2493.15	2186.35	3643.92	3904.20
